# Comparative Proteomics Reveals Evidence of Enhanced EPA Trafficking in a Mutant Strain of *Nannochloropsis oculata*

**DOI:** 10.3389/fbioe.2022.838445

**Published:** 2022-05-12

**Authors:** Wan Aizuddin Wan Razali, Caroline A. Evans, Jagroop Pandhal

**Affiliations:** ^1^ Department of Chemical and Biological Engineering, University of Sheffield, Sheffield, United Kingdom; ^2^ Faculty of Fisheries and Food Science, Universiti Malaysia Terengganu, Terengganu, Malaysia

**Keywords:** Nannochloropsis, eicosapentaenoic acid (EPA), ethyl methanesulfonate (EMS) random mutagenesis, cerulenin, galvestine-1, label-free quantitative (LFQ) proteomic analysis

## Abstract

The marine microalga *Nannochloropsis oculata* is a bioproducer of eicosapentaenoic acid (EPA), a fatty acid. EPA is incorporated into monogalactosyldiacylglycerol within *N. oculata* thylakoid membranes, and there is a biotechnological need to remodel EPA synthesis to maximize production and simplify downstream processing. In this study, random mutagenesis and chemical inhibitor-based selection method were devised to increase EPA production and accessibility for improved extraction. Ethyl methanesulfonate was used as the mutagen with selective pressure achieved by using two enzyme inhibitors of lipid metabolism: cerulenin and galvestine-1. Fatty acid methyl ester analysis of a selected fast-growing mutant strain had a higher percentage of EPA (37.5% of total fatty acids) than the wild-type strain (22.2% total fatty acids), with the highest EPA quantity recorded at 68.5 mg/g dry cell weight, while wild-type cells had 48.6 mg/g dry cell weight. Label-free quantitative proteomics for differential protein expression analysis revealed that the wild-type and mutant strains might have alternative channeling pathways for EPA synthesis. The mutant strain showed potentially improved photosynthetic efficiency, thus synthesizing a higher quantity of membrane lipids and EPA. The EPA synthesis pathways could also have deviated in the mutant, where fatty acid desaturase type 2 (13.7-fold upregulated) and lipid droplet surface protein (LDSP) (34.8-fold upregulated) were expressed significantly higher than in the wild-type strain. This study increases the understanding of EPA trafficking in *N. oculata*, leading to further strategies that can be implemented to enhance EPA synthesis in marine microalgae.

## Introduction

Eicosapentaenoic acid (EPA) is an omega-3 long-chain polyunsaturated fatty acid (LC-PUFA) found in fish oils and well documented to provide multiple benefits for human health (Shi et al., 2021). However, the use of fish oils as a source of EPA has many issues, including declining fish stocks, contamination by heavy metals and organic pollutants, unpleasant smells, and unsuitability for vegetarian markets (Kaye et al., 2015; Shi et al., 2021). Therefore, there is a drive to obtain omega-3 (ω-3) directly from their primary source, microalgae. Microalgae are well-known sources of various high-value bioactive compounds, including LC-PUFA, carotenoids, proteins, polyphenols, phytosterols, hormones, and vitamins (Levasseur et al., 2020). Due to their metabolic capacity and relatively simple structures, microalgae are considered the most efficient “plants” on Earth in capturing sunlight energy (Vecchi et al., 2020), growing up to five to ten times faster than land plants (Adamczyk et al., 2016) and enhancing biological carbon fixation by utilizing carbon dioxide in the atmosphere (Kumar et al., 2010). A few microalgae species have been identified to synthesize relatively high amounts of LC-PUFA, although efforts to increase titers are increasingly sought to improve economic viability.

*Nannochloropsis* species are relatively small (2–4 µm diameter) unicellular microalgae that contain ovoid- or cup-shape chloroplasts (Iwai et al., 2015). Reports demonstrate species with a relatively high concentration of ω-3 EPA of up to 40 mg/g dry cell weight (DCW) (Chini Zittelli et al., 1999; Kent et al., 2015). In an *N. oceanica* strain, 30% of total fatty acids (TFA) were quantified as ω-3 EPA (Kaye et al., 2015), while an *N. oculata* strain produced the highest reported percentage at 40% EPA of TFA (Renaud et al., 1991). However, in order to increase EPA production in microalgae, it is important to understand its synthesis from a metabolic and spatial perspective.

Fatty acid (FA) synthesis occurs in chloroplasts, producing up to 18 carbon chain lengths, whereafter EPA is reportedly elongated in the endoplasmic reticulum (ER) prior to being imported back into the chloroplasts for incorporation into the thylakoid membrane polar lipid, monogalactosyldiacylglycerol (MGDG) (Sayanova et al., 2017). The initial synthesis of FAs is catalyzed by fatty acid synthase (FAS) and acetyl CoA carboxylase (Chaturvedi and Fujita, 2006). Ketoacyl-acyl carrier protein (ACP) synthase (KAS) is responsible for the elongation of medium-chain FAs up to C16:0 in the chloroplasts (Nofiani et al., 2019). The elongation of C18:0 to EPA in *Phaeodactylum* consists of two routes, while one route is suggested for *N. gaditana* (Dolch et al., 2017). The main suggested route for *N. gaditana* is *via* the ω-6 pathway (Janssen et al., 2020). The process consists of step-wise conversion from C16:0 to C18:0, C18:1 ^Δ9^, C18:2 ^Δ9,12^ (linoleic acid), C18:3 ^Δ6,9,12^ (γ-linolenic acid), C20:3 ^Δ8,11,14^ (dihomo-γ-linolenic acid), C20:4 ^Δ5,8,11,14^ (arachidonic acid), and C20:5 ^Δ5,8,11,14,17^ (EPA) *via* the actions of Δ0-elongase (ELO), stroma stearoyl-ACP Δ9-desaturase (SAD), or an ER fatty acid desaturase (ERΔ9FAD), Δ12FAD, ERΔ6FAD, Δ6-ELO, ERΔ5FAD, and ERω3FAD, respectively (Dolch et al., 2017).

A study on *N. oceanica* found that during the exponential growth phase, MGDG, phosphatidylcholine (PC), and phosphatidylglycerols (PG) are the main membrane polar lipids (Han et al., 2017). However, in the thylakoid membranes, MGDG contributes approximately 40–50% to these membrane lipids. A study in the same strain found that 60% of MGDG is enriched EPA (Junpeng et al., 2020). In a related strain, *N. gaditana*, it was found that EPA resides in membrane polar lipids during the exponential phase but translocates to neutral lipids, triacylglycerols (TAG), toward the end of batch growth (Janssen et al., 2019). An attempt to discover the relationship between EPA and membrane polar lipids in *N. gaditana* demonstrated that MGDG production relies on EPA supplied from the ER to the chloroplast (Dolch et al., 2017). Hence, EPA synthesis and translocation is a highly regulated and complex process. This means that cell engineering approaches to increase EPA content in microalgae have resulted in limited success. Gene overexpression studies in *N. oceanica* included targeting Δ12 desaturase (Kaye et al., 2015), Δ6 desaturase (Yang F. et al., 2019), Δ6 elongase (Shi et al., 2021), and combined Δ5, Δ9, and 12Δ desaturase (Poliner et al., 2018). Most of these genetically engineered strains showed a slight increase in the EPA content compared to the wild-type strain, although overexpression of Δ6 desaturase even reduced the EPA percentage of TFA in total lipids compared to the wild type (Yang F. et al., 2019).

When highly regulated metabolic pathways are involved or genetic tool kits for engineering specific strains are not available, random mutagenesis with selection or screening is an attractive option to generate desirable phenotypes. Gamma-ray (Park et al., 2021), UV ray (Bougaran et al., 2012), ^137^Cs–γ nuclear radiation (Lu et al., 2020), atmospheric and room temperature plasma (ARTP) (Zhang et al., 2014), heavy-ion irradiation-mediated mutagenesis (Song et al., 2018), and chemicals (Chaturvedi et al., 2004; Wu et al., 2019) are mutagens that have successfully been used to generate genetic diversity in microalgae. The challenge then becomes the implementation of successful selection strategies. Enzyme inhibitors have shown increasing promise as a strategic selection method following random mutagenesis (Chaturvedi et al., 2004; Chaturvedi and Fujita, 2006; Li et al., 2015; Fu et al., 2016). This is because enzyme inhibitors can limit the function of a single targeted enzyme without influencing the operation of other enzymes (Kukorelli et al., 2013; Arora et al., 2020).

FAS inhibitors have been used previously to select microbial strains with re-wired metabolism that have enhanced the accumulation of specific FAs. For example, cerulenin, a FAS inhibitor, and an oxidant triphenyl tetrazolium chloride (TTC) were used in combination to screen *Mortierella alpina* mutant cells to select strains with higher arachidonic acid (ARA) content, an omega-6 component that has the same carbon length as EPA (Li et al., 2015). TTC is an oxidant that can be ingested and oxidized to a red molecule by living cells. Cerulenin changed the FA composition by affecting FA degradation in a *Colwellia psychrerythraea* strain, where C16:1 was reduced by 12.6% of TFA, and DHA increased by 7.8% of TFA (Wan et al., 2016). Mutants with a standard growth rate in the presence of cerulenin were identified as having higher FAS activity than wild-type species (Li et al., 2015). Cerulenin was also reported to interfere with the lipid metabolism, which caused the increase in ARA in *Mortierella alpina* from 39 to 45.6% of TFA (Li et al., 2015). Cerulenin is also effective in inhibiting β-ketoacyl-ACP synthase (KAS I, KAS II, and KAS III) (Meng et al., 2018). Cerulenin was also reported as the first treatment followed by N-methyl-N-nitro-nitrosoguanidine (NTG) mutagenesis in *Shewanella electrodiphila*, where the EPA increased from 20 to 30 mg/g DCW (Zhang and Burgess, 2017). However, equivalents are not known for selecting long-chain FA changes in microalgae species. Another inhibitor previously tested to screen for *N. oculata* mutants with elevated lipid content was acetyl-CoA carboxylase (ACCase) inhibitor Quizalofop. The quantity of EPA in the mutant was not measured; however, the level of ARA in the mutant was considerably higher than the wild-type strain (Moha-León et al., 2019)*.* Considering the EPA content in MGDG; MGDG synthase inhibitors could also be used as selective agents. MGDG synthase inhibitors include citraconic anhydride, N-ethylmaleimide, ortho-phenanthroline, S-nitroso-N-acetyl penicillamine, and galvestine-1 (Coves et al., 1988; Marechal et al., 1995; Boudière et al., 2012). Although galvestine-1 was shown to effectively inhibit MGDG synthase, reducing MGDG quantity in *Arabidopsis thaliana* (Botté et al., 2011), no previous research has been conducted to investigate the effect of galvestine-1 on MGDG synthesis in microalgae.

In this study, *N. oculata* was selected as it can synthesize relatively high levels of EPA content compared to other microalgae species (Adarme-Vega et al., 2012), and previous studies have successfully applied random mutagenesis in this strain (Chaturvedi et al., 2004; Chaturvedi and Fujita, 2006). Moreover, *N. oculata* is one of the most widely used microalgae in aquaculture hatcheries demonstrating industrial robustness (Babuskin et al., 2014). This work reports a random mutagenesis approach by chemical mutagen ethyl methanesulfonate (EMS), combined with the combined use of two specific enzyme inhibitors of lipid metabolism, cerulenin, and galvestine-1, for the first time, for screening the mutants. The initial aim was to develop an improved *N. oculata* strain that is able to produce enhanced EPA levels compared to wild-type cells without comprising growth rates and subsequently apply cross-species quantitative proteomics to generate specific hypotheses on how metabolism has been re-wired to generate the phenotype.

## Materials and Methods

### Algal Strain and Culture Conditions

*N. oculata* (849/1) was provided by the Culture Center of Algae and Protozoa (CCAP, Scotland) and was cultured in modified f/2 medium composed of the following: 33.5 g/L artificial seawater salt (Ultramarine Synthetic Sea Salt, Waterlife, United Kingdom), 75 mg/L NaNO_3_, 4.35 mg/L NaH_2_PO_4_.2H_2_O, enriched with trace elements (4.16 mg/L Na_2_EDTA, 3.16 mg/L FeCl_3_.6H_2_O, 0.01 mg/L CuSO_4_.5H_2_O, 0.022 mg/L ZnSO_4._7H_2_O, 0.01 mg/L CoCl_2_.6H_2_O, 0.18 mg/L MnCl_2_.4H_2_O, and 0.006 mg/L Na_2_MoO_4_.2H_2_O) and vitamins (0.1 mg/L thiamine HCl (B_1_), 0.005 mg/L cyanocobalamin (B_12_), and 0.0005 mg/L biotin). The stock culture was maintained in a 500 ml conical flask and bubbled with 0.22 µm filtered air for aeration and mixing. The incubation temperature was 20°C, and the light intensity was 100 to 110 μmol m^−2^ s^−1^ range for 12-h light/dark cycles. The stock culture was refreshed every week to maintain the culture in the exponential growth phase. All the chemicals used in this study were purchased from Sigma–Aldrich, unless otherwise stated.

### Mutagenesis and Selection of EPA-Overproducing Mutant Strains

Cells in the early exponential phase (7 × 10^6^ cells/ml) were refreshed with sterile f/2 medium and centrifuged at 3,488 × *g* for 5 min, and EMS was added to make a final concentration of 100, 200, and 300 mM. The cells were mutagenized for 60 min, washed three times with sterile f/2 medium, and allowed to grow for seven days before initiating selection. The sub-lethal chemical concentrations were determined by measuring optical density (OD) at 595 nm using a GENios Tecan plate reader (TECAN, Germany) (Supplementary Figures S1A, S1B). Equal cell numbers (2 × 10^7^ cells/ml) were spread uniformly on f/2 medium plates (1.5% w/v) containing 50 µM of cerulenin. After three weeks of incubation at 20°C, the number of resistant colonies from each plate was counted. The countable plate was selected, and each colony was inoculated in a 3 ml f/2 medium containing 50 µM of cerulenin in 24-well plates placed under light (130 μmol m^−2^ s^−1^). The absorbance was measured at 595 nm on the plate reader for ten days. The mutant colonies with a higher optical density at 595 nm than wild-type *N. oculata* were selected and cultured in f/2 media containing a higher concentration of cerulenin, 60 µM. The three fastest-growing mutants were selected for the next stage using an MGDG synthase inhibitor, galvestine-1 (Botté et al., 2011; Boudière et al., 2012). The mutants were cultured in f/2 media containing 10 µM of galvestine-1, and the fastest-growing mutant was selected for further studies. The growth rate (µ/day) was calculated as follows:

$$\mu =\frac{\ln\left(Wf/Wi\right)}{\Delta t},$$

where *Wf* and *Wi* were the final and initial OD at 595 nm, respectively, and Δ*t* was the cultivation time in the day (Chiu et al., 2009). The mutant with the fastest growth and EPA content was selected for further studies.

### Photobioreactor Setup for Selecting Two-Time Points for Label-Free Quantification Proteomics Experiments

**FIGURE 1:**
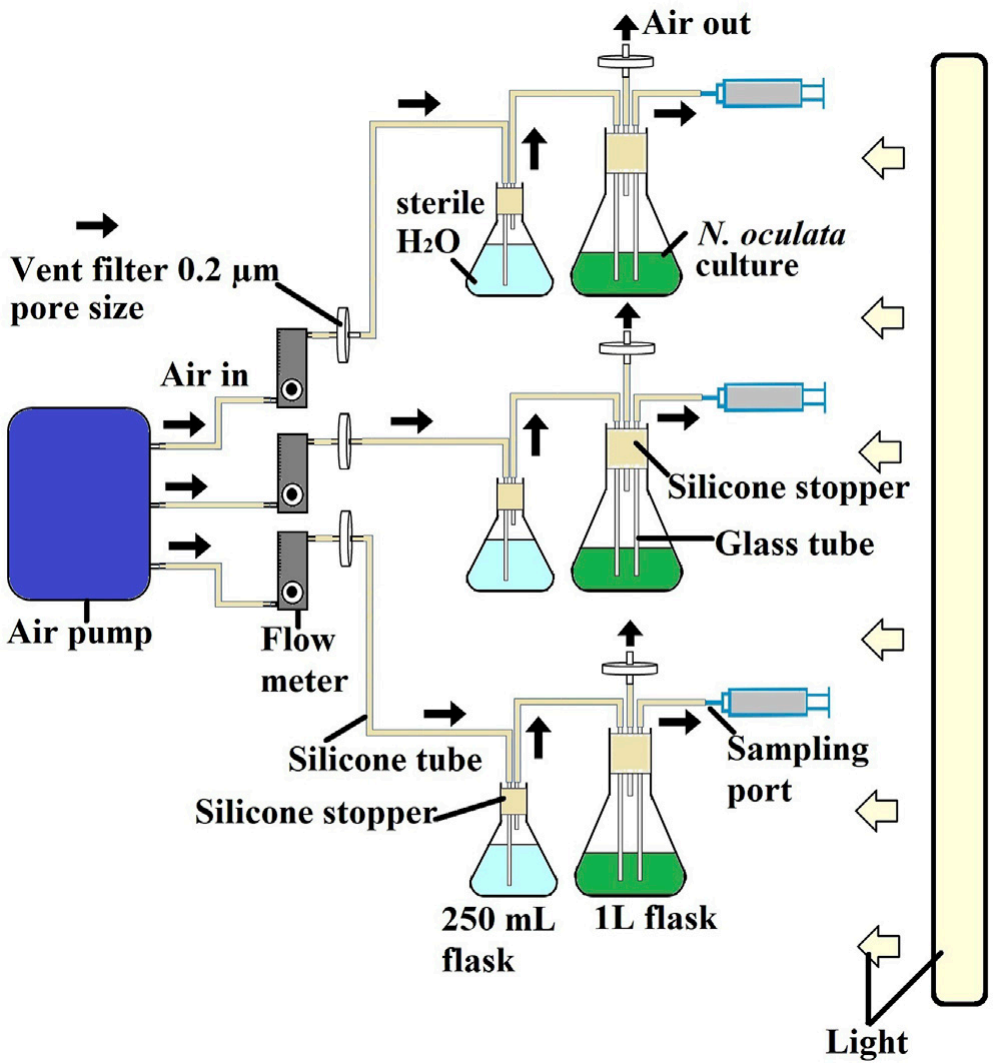
Schematic diagram of experimental cultivation system for selecting two-time points for LFQ proteomics experiments. All experiments were conducted at room temperature at 20°C and illuminated under 130 μmol μmol m^−2^ s^−1^ under a 12-h/12-h (day/night) cycle. The cultures were subjected to continuous filtered aeration and bubbled at 2 L/min.

*N. oculata* cultures were set up in triplicates using a 1-L flask photobioreactor system (Figure 1). The starting optical density at 595 nm was 0.3. All flasks were maintained at a temperature of 20 °C and illuminated under 130 μmol μmol m^−2^ s^−1^ under a 12-h/12-h (day/night) cycle. Cultures were subjected to continuous filtered aeration and bubbling at 2 L/minute. The algal culture was aerated and mixed in the same way as the pre-culture. The optical density at 595 nm, and pH was monitored using a portable pH meter LAQUA B-712 (Horiba, Moulton Park, United Kingdom) every day throughout the experiments over a 12-day period. The samples were taken on days 3, 5, 7, 9, and 12 for wild-type *N. oculata* and days 2, 5, 7, 9, and 12 for the selected mutant *N. oculata*. The sample was taken on day 2, considering that the selected mutant grew faster than the wild-type *N. oculata.* Then 5 ml culture was taken for each analysis of DCW, proteins and chlorophylls, lipids, and EPA. A sample volume of 50 ml was taken for LFQ proteomics analysis. The samples were collected in three biological replicates for all analyses. All the sampling was done during the dark period, 2 h before the light period started. Harvested cells pellets (centrifuge at 4,415 × *g* for 5 min) were washed with phosphate-buffered saline and centrifuged (11,337 × *g* for 2 min) prior to storage at −20°C, while proteomic samples were kept at −80°C until further analysis.

### Analytical Methods

Cell pellets were freeze-dried for 24 h by using a freeze drier (LyoQuest, Telstar, United Kingdom), and the DCW was measured using a microbalance (CPA2P, Sartorius, OH, United States).

Chlorophylls and proteins were quantified by using the spectrophotometric method in triplicate (Chen and Vaidyanathan, 2013). In brief, cell pellets were lysed by glass bead-beating using a cell disruptor (DISRUPTOR GENIE^®^, United States). The samples were saponified by heating at 100°C for 30 min (Digital Drybath, Thermo Fisher Scientific, United Kingdom). An aliquot was used for protein assay, and the remaining sample was mixed with chloroform and methanol (ratio 2:1, v/v), vortexed (2 min), centrifuged (12,000 × *g*, 2 min), and the top aqueous phase was used for chlorophyll assays.

Methods for quantification of nitrate (Collos et al., 1999) and phosphate (Strickland and Parsons, 1972) in an f/2 medium were adapted from previous studies. The supernatants from the harvested samples were kept after filtration through a 0.22 µm syringe filter (Millex, United Kingdom). The concentrations of nitrate and phosphate were determined for each sampling day by measuring the absorbance values at 220 and 885 nm, respectively.

### Determination of Fatty Acid Methyl Ester

The method for determining fatty acid methyl ester (FAME) was adapted from a previous study with slight modifications (Griffiths et al., 2010). In brief, 300 µl toluene was added to the 2 ml Eppendorf tube containing wet microalga biomass. The Eppendorf tube then vortexed for 2 min and continued by adding 300 µl of sodium methoxide. The mixture was then transferred into the 2 ml glass vial and incubated at 80°C for 20 min. After that, the vials were kept a while at room temperature for cooling. 300 µl boron trifluoride was added to the vial and incubated again at 80°C for 20 min. In the meantime, 300 µl HPLC-grade water and 600 µl hexane were added to other prepared empty 2 ml Eppendorf tubes. The mixture in the vial was transferred to the prepared Eppendorf tube containing water and hexane and then centrifuged at 7,916 × *g* for 10 min. Then, 750 µl organic phase (upper hexane-toluene layer) was transferred to a new labeled Eppendorf tube. The extract was then dried using inert nitrogen gas and stored at −20°C until further analysis.

A measure of 80 µl of toluene was added to the extracted sample and vortexed to ensure that all the extracts were well-mixed. The mixture was then centrifuged at 11,337 × *g* for 2 min. A quantity of 35 µl FAME was transferred into a GC vial and was identified and quantified using a Thermo Finnigan TRACE 1300 GC-FID System (Thermo Fisher Scientific, United Kingdom) onto a TR-FAME capillary column (25 m × 0.32 mm × 0.25 µm). Then 1 µl of Supelco 37 Component FAME Mix standard was injected as a reference and 1 µl of sample volume was injected in split injection mode at 250°C. The split flow was 75 ml/min. The GC was operated at a constant flow of 1.5 ml/min helium. The temperature program was started at 150°C for 1 min, followed by temperature ramping at 10°C/min to a final temperature of 250°C, and held constant at 250°C for 1 min. The total analysis time was 15 min, and the standard 37 FAME was injected for all 24 samples to ensure that the system was working correctly.

The peak identities were ascertained for data interpretation and analysis using external Supelco 37 Component FAME Mix standard, C16, C18, and C20:5 standards. The peak areas were integrated using a chromatography data system, Chromeleon 7 software (Thermo Fisher Scientific, United Kingdom). Based on the known amount value of 37 FAME components, C16, C18, and C20:5 standards, a ratio was established between the area and the amount. The amount of unknown components in the microalgal extract was then determined by their peak areas and calculated in mg/g DCW.

The quantification of FAME within TAG and polar lipids was adapted from a previous study with modifications (Janssen et al., 2019). In brief, total lipids were extracted from wet microalgae biomass using a standard Folch method (Axelsson and Gentili, 2014). C17:0 PC and C17:0 triheptadecanoin were added to the samples prior to the extraction of total lipids. Then 2 ml of methanol was added to the samples and homogenized for 1 min using a homogenizer Ultra-Turrax^®^ T 25 (Ultra-Turrax, Germany), followed by the addition of 4 ml of chloroform and further homogenization for 2 min. The total lipid solution was filtered through a 0.22-µm filter (SLS, United Kingdom). The cell debris were rinsed with 2 ml fresh solvent (chloroform and methanol, ratio 2:1, v/v) and combined with the previous filtrate. 2 ml of potassium chloride solution (8.8 g/L) was added, and the mixture vortexed for 1 min. The top solvent layer was discarded, and the bottom solvent was evaporated using a centrifugal evaporator (Jouan, United States). The total lipid extract was dissolved in chloroform and spotted onto a thin liquid chromatography (TLC) plate along with TAG and polar lipid standard. The mobile phase used was iso-hexane, ether, and formic acid (80:20:2, v/v/v) to separate the TAG and polar lipids. The TAG and polar lipid fractions were removed by scraping the silica into test tubes, followed by re-extraction using iso-hexane and ether (1:1, v/v) and chloroform, methanol, and distilled water (5:5:1, v/v/v), respectively. A total of 1 ml of toluene and 2 ml of 1% sulfuric acid in methanol were added for transesterification, and the samples were incubated at 50°C for 16 h. Then 5 µl FAME sample was identified and quantified using a GG, Agilent 6890 model (Agilent Technologies, United States), onto a CP-Wax (52 CB) GC column (30 m × 0.25 mm × 0.15 µm). In total, 1 µl of FAME standard (Nu-Chek Prep, United States) was injected as a reference, and 1 µl of sample volume was injected in split injection mode at 230°C. The GC was operated at a constant flow of 1 ml/min hydrogen. The temperature program was started at 170°C for 3 min, followed by temperature ramping at 4°C/min to a final temperature of 220°C and held constant at 220°C for 10 min. Peak areas were integrated using a chromatography data system, Agilent Chemstation software (Agilent Technologies, United States). The EPA of TFA in TAG and polar lipids were determined by their peak areas and quantified against the added internal standard.

### Statistical Analysis for Growth Profiles

Statistical differences for all growth profiles data, percentages, and quantification of EPA were performed by Student’s *t*-test, with three replicates (*n* = 3). Array 1 was for wild-type *N. oculata,* and array 2 was for the selected mutant *N. oculata*; 2-tails and type 1 were set using Microsoft^®^ Excel^®^ for Microsoft 365 MSO (Washington, United States). The data were considered significant when the *p*-value was at least <0.05.

### Protein Extraction and Quantification

In total, 50 ml microalga samples were harvested at early exponential phase on day 2 and late exponential phase on day 12 for the selected mutant *N. oculata*, and at early exponential phase on day 3 and late exponential phase on day 12 for wild-type *N. oculata* in triplicates *via* centrifugation at 4,000 *g* for 15 min at 4°C. The supernatants were discarded, and the samples were kept at −80°C until further use. Crude proteins were extracted as described previously (Posch, 2014). A measure of 1 ml of lysis buffer (2% sodium dodecyl sulfate (SDS), 40 mM Tris base, and 60 mM dithiothreitol (DTT)) and 10 µL Halt™ protease inhibitor cocktail (Thermo Fisher Scientific, United Kingdom) were added to the samples pellets and put on ice for thawing. 500 µl glass beads having sizes 425–600 µm were added to the sample tubes. The samples were vortexed for 20 cycles (30 s vortexed and then 30 s cooled on ice). Lysed samples were centrifuged at 18,000 × *g* for 5 min at 4°C. The samples were kept on ice for 30 min until the foam subsided. The supernatants (crude protein) were transferred to 1.5-ml protein LoBind Eppendorf tubes and stored at −80°C.

Two sets of samples were purified from lipids, pigments, and other contaminants by using a protein 2D clean-up kit (GE Healthcare, United States) by following the manufacturer’s protocols. The 2D cleaned-up protein pellets were resuspended in 100 µl urea buffer (8 M urea, 100 mM Tris–HCl (pH 8.5), and 5 mM DTT) for 1D SDS-PAGE and in-solution digestion, respectively. The samples were incubated in a sonication bath for 5 min to dissolve protein into the urea buffer. Then, the proteins samples were quantified using a NanoDrop™ 2000 (Thermo Fisher Scientific, United Kingdom) spectrophotometer. The spectrophotometer setting was set as 1 mg/ml equals to 1 optical density reading at 280 nm. BSA was used to create a standard curve with urea buffer (1, 0.8, 0.6, 0.4, 0.2, 0.1 mg/ml) (Supplementary Figures S2A, S2B).

### In-Solution Digestion

A measure of 50 µg protein was transferred to a sterile protein LoBind Eppendorf tube. Protein samples were diluted to 10 µl with urea buffer and incubated at 37°C for 30 min. Then 1 µl 100 mM iodoacetamide was added and incubated in the dark for 30 min at 20°C to S-alkylate the protein samples. A measure of 2 µg Trypsin/Lys-C mix (Promega, United States) was added to the protein solutions and incubated for 3 h at 37°C; 75 µl (50 mM Tris–HCl (pH 8.5), 10 mM CaCl_2_) was added to the protein solution and incubated overnight (16–20 h) at 37°C for trypsin digestion (Hitchcock et al., 2016); 5 µg of protein samples were run via 1D SDS-PAGE to confirm that the protein was digested; 4.8 µl (5% of the total protein solution) of 10% TFA was added to the protein solution to stop the digestion process. Pierce^®^ C18 spin columns (Thermo Fisher Scientific, United Kingdom) were used for desalting the samples by following the manufacturer’s protocols; and 60 µl purified protein samples were collected from the spin columns. The samples were dried using a vacuum evaporator (Concentrator plus, Eppendorf, Germany) and stored at −80°C for further mass spectrometry analysis.

### LC-MS/MS for Proteomics

LC-MS/MS was performed and analyzed by nano-flow liquid chromatography (U3000 RSLCnano, Thermo Fisher Scientific, United Kingdom) coupled to a hybrid quadrupole-orbitrap mass spectrometer (Q Exactive HF, Thermo Fisher Scientific, United Kingdom). Peptides were separated on an Easy-Spray C18 column (75 μm × 50 cm) using a 2-step gradient from 3% solvent A (0.1% formic acid in water) to 10% B over 5 min and then to 50% solvent B (0.1% formic acid in 80% acetonitrile) over 180 min at 300 nl min-1, 40°C. The mass spectrometer was programmed for data-dependent acquisition with 10 product ion scans (resolution 30,000, automatic gain control 1e5, maximum injection time 60 ms, isolation window 1.2 Th, normalized collision energy 27, and intensity threshold 3.3e4) per full MS scan (resolution 120,000, automatic gain control 1e6, maximum injection time 60 ms) with a 20-s exclusion time. Each sample was run in triplicate.

### Protein Identification and Generation of Label-Free Quantification Quantitative Proteomic Data

Mass spectrometry data in *.raw format were processed using MaxQuant v. 1.6.17 integrated with the MaxLFQ algorithm. Proteins were identified by searching the MS data files against an in-house constructed *Nannochloropsis* proteome database. The proteome database is a combination of *Nannochloropsis* strains downloaded from NCBI (December 2020), UniProt (December 2020), and extracted from the MSPnr100 database (Tran et al., 2016). The total number of protein sequences in the combined *Nannochloropsis* proteome database was 16,270 proteins. A 1% FDR was applied, and default settings were applied. Search parameters specified tryptic cleavage, carbamidomethyl-Cys (fixed modification), Met oxidation, and protein N-terminal acetylation (variable modifications) with a maximum of two missed cleavages. In total, seven amino acids were set at the minimum peptide length.

### Statistical Analysis of Label-Free Quantification Quantitative Proteomic Data

Statistical analyses were performed using LFQ-Analyst (https://bioinformatics.erc.monash.edu/apps/LFQ-Analyst/), whereby the LFQ intensity values were used for protein quantification. The missing values were replaced by values drawn from a normal distribution of 1.8 standard deviations and a width of 0.3 for each sample (Perseus-type). Differential expression analysis was performed using protein-wise linear models combined with empirical Bayesian statistics using the Bioconductor package limma. The Benjamini–Hochberg method of FDR correction was used. The adjusted *p*-value cutoff was set at 0.05, and the log2 fold change cutoff was set at 1.

## Results and Discussion

### Growth Profiles of Wild-Type and M1 Mutant *N. oculata*

**FIGURE 2:**
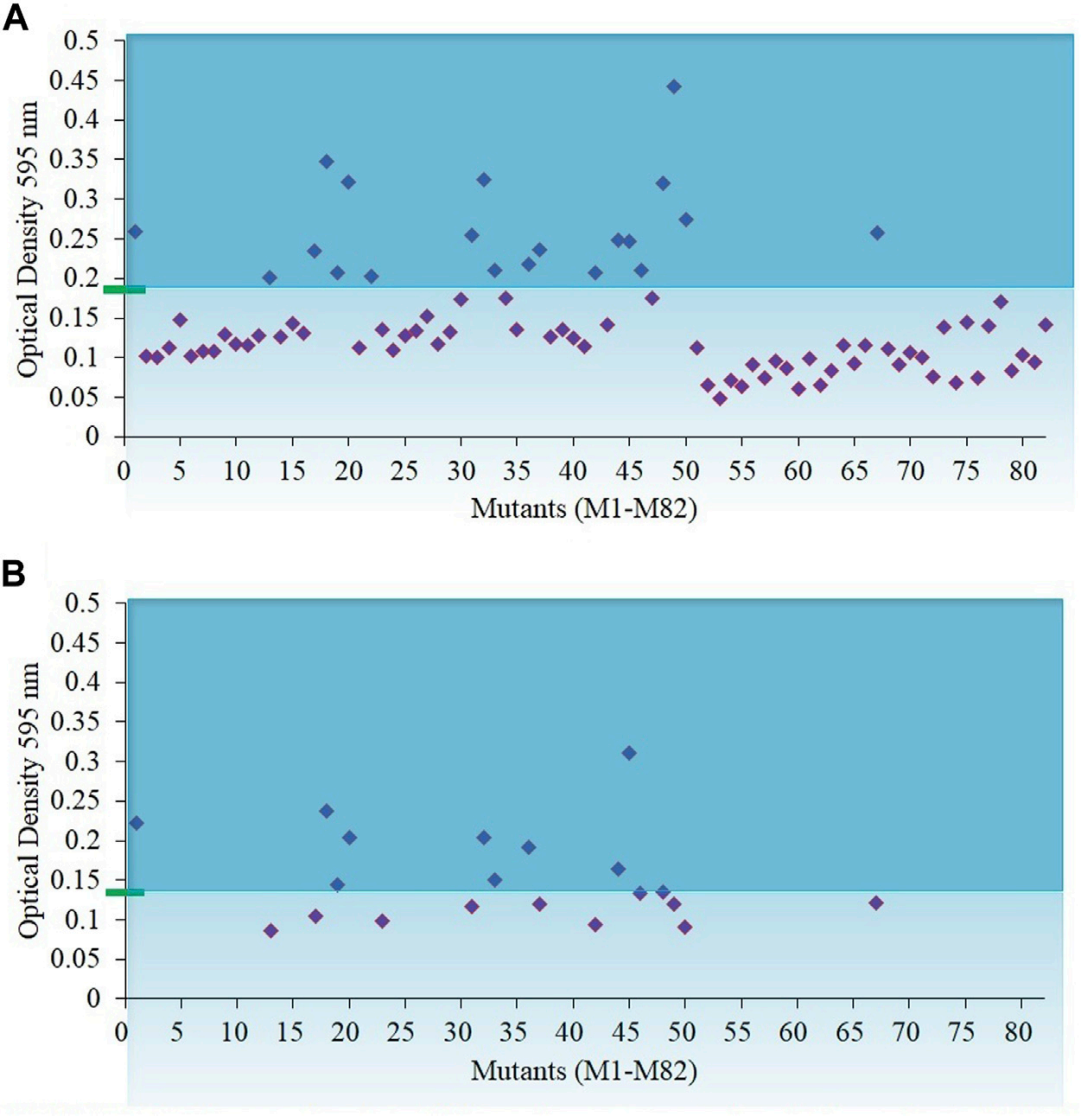
Biomass density of *N. oculata* mutants after eight days of growth in f/2 medium containing cerulenin. The initial optical density of 595 nm was 0.15 at day 0 **(A)** Optical density of 82 *N. oculata* mutants in 50 µM cerulenin. **(B)** Optical density of 20 *N. oculata* mutants containing 60 µM cerulenin. The cultures were incubated at 130 μmol m^−2^s^−1^, 20°C, under a 12-h/12-h (light/dark) cycle. The green rectangle and blue line represent the biomass density of the wild-type *N. oculata*.

**FIGURE 3:**
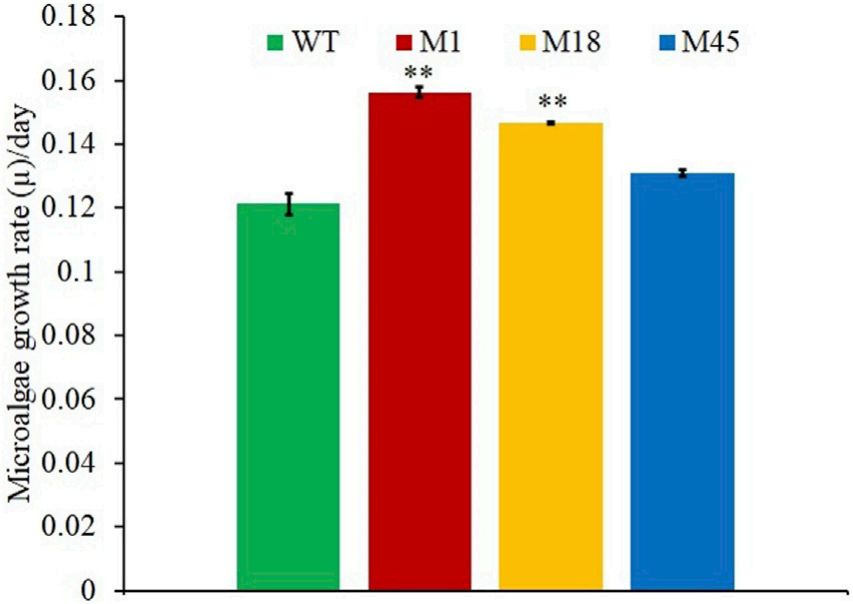
Growth rate per day comparisons of mutants M1, M18, M45, and wild-type *N. oculata*, incubated at 130 μmol m^−2^s^−1^, 20°C, under a 12-h/12-h (light/dark) cycle and 160 RPM shaking for ten days. Mean ± standard deviation is shown (*n* = 3) and *t*-tests determine the statistical significance (*p* < 0.05 [*]; *p* < 0.01 [**]; and *p* < 0.001 [***]) of the M1 mutant strain compared to the wild-type strain.

Mutants of wild-type *N. oculata* were randomly generated using EMS, and desirable phenotypes were first screened using the FAS inhibitor, cerulenin, with the hypothesis that traits such as alternative mechanisms or increased synthesis of EPA and TAG would be selected. A total of 82 colonies were counted on f/2 medium agar plates with 200 mM EMS and 50 µM cerulenin after three weeks of incubation (Supplementary Figure S3B); 100 and 300 mM EMS concentrations generated too many or no mutants, respectively (Supplementary Figures S3A, S3C). Finally, 200 mM EMS was selected as the concentration to generate mutants. All 82 colonies from the 200 mM EMS agar plate were isolated and grown in 24-well plates in f/2 media containing 50 µM cerulenin and cultivated under 130 μmol m^−2^ s^−1^ illumination (12-h light/dark cycle) at 20°C. After ten days, 20 mutants were recorded with a higher OD (595 nm) than the wild-type *N. oculata* cells (Figure 2A). These 20 mutants were subsequently cultivated in f/2 media containing a higher cerulenin concentration, 60 μM, and three mutants (labeled M1, M18, and M45) that reached the highest OD (595 nm) were further selected for the next stage of screening using galvestine-1 (Figure 2B). The selected three mutants were cultured for ten days in an f/2 medium containing 10 µM galvestine-1, the sub-lethal concentration for wild-type *N. oculata*. M1 and M18 mutants’ growth rate per day were statistically significantly higher than wild-type cells, with a *p*-value less than 0.01 (Figure 3). The FA profile of M1, M18, and M45 mutant strains showed elevated levels of EPA compared to the wild-type strain (Supplementary Figures S4, S5). M1 mutant recorded the highest EPA (33.6%) of TFA in total lipids compared to other mutants and the wild-type strain. Hence, M1 mutant *N. oculata* was selected for further growth and FAME analysis before LFQ proteomics analysis.

**FIGURE 4:**
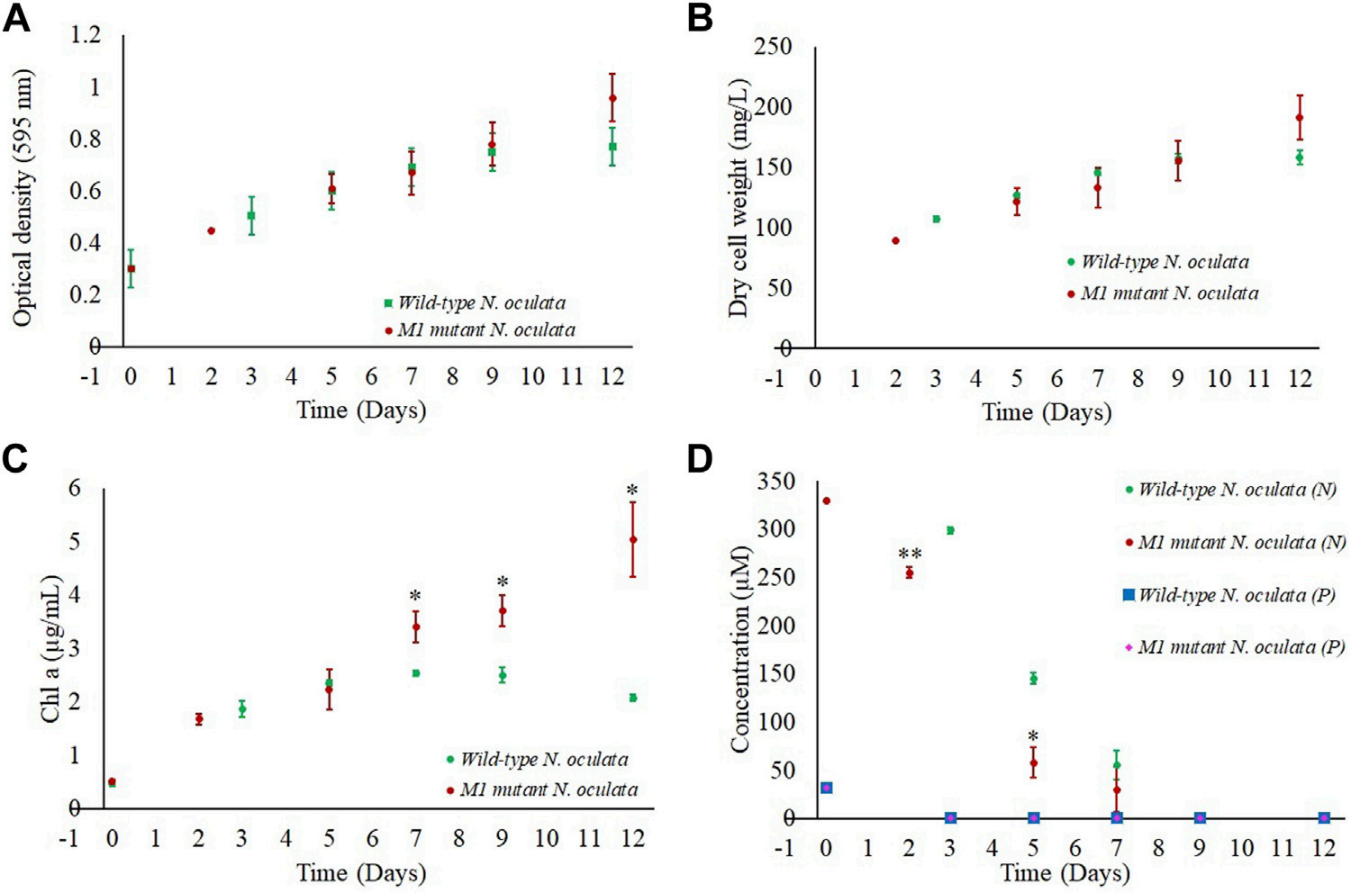
Growth profiles for wild-type and M1 mutant *N. oculata* cultivated in 1-L flasks under 150 μmol m^−2^ s^−1^, 20°C, and aerated bubbling for mixing and carbon source for 12 days. **(A)** Growth curves illustrated by optical density at 595 nm and **(B)** DCW. Comparison of wild-type and M1 mutant *N. oculata* over 12 days of culturing for **(C)** chlorophyll concentration and **(D)** phosphate (P) and nitrate (N) uptake profiles. Mean ± standard deviation is shown (*n* = 3) and *t*-tests determine the statistical significance (*p* < 0.05 [*]; *p* < 0.01 [**]; and *p* < 0.001 [***]) of the M1 mutant strain compared to the wild-type strain.

Wild-type and M1 mutant *N. oculata* cells were cultivated in 1-L flasks in triplicate at 150 μmol m^−2^ s^−1^ under a 12-h/12-h (day/night) cycle for 12 days. The growth performance, nutrient uptake, and changes of FAME profiles were monitored in order to select two-time points where EPA and TAG synthesis were at an optimum level and where differential protein expression analysis would provide insight into novel cellular adaptations. Even though the M1 mutant showed a higher growth rate than wild-type cells during screening in 24-well plates, and despite a higher final DCW in the mutant strain (day 12), no statistical significance was observed (Figures 4A,B). However, chlorophyll *a* in the M1 mutant was statistically significantly higher than wild-type cells from day 7 onward (Figure 4C), suggesting more efficient light-harvesting and photosynthetic capability than the wild-type.

The pH was recorded around 7–8.5 for both the wild-type and M1 mutant cultures*,* suggesting that the aeration was sufficient to control the pH at the optimum conditions. A pH of 8.5 was previously reported to be optimal for *Nannochloropsis* sp. growth (Khatoon et al., 2014).

Nitrate and phosphate concentrations in the f/2 medium were also monitored during growth. Although these macronutrients are essential for microalgae growth (Hu and Gao, 2006), their uptake rates could affect the FA profiles (Rasdi and Qin, 2015). Nitrate was rapidly consumed until day 5 and was below detection limits by day 9 of culturing (Figure 4D). The M1 mutant displayed a faster nitrate uptake, with the nitrate concentration decreasing in the media from 330 to 255 μM during two days of culturing, whereas the nitrate in the wild-type *N. oculata* flasks reduced from 330 to 299 μM within three days. Nitrate limited condition in the f/2 medium after day 9 could trigger the synthesis of neutral lipids, as observed in several oleaginous microalgae (Burch and Franz, 2016; Tran et al., 2016; Remmers et al., 2017).

Similarly, phosphate concentrations reduced to below detectable limits during growth within 2 and 3 days for M1 mutant and wild-type cells, respectively (Figure 4D). Overall, the nitrate and phosphate results suggested that the wild-type and M1 mutant strains had completed their batch growth over nine days of culturing.

### Fatty Acid Methyl Ester Profiles

**FIGURE 5:**
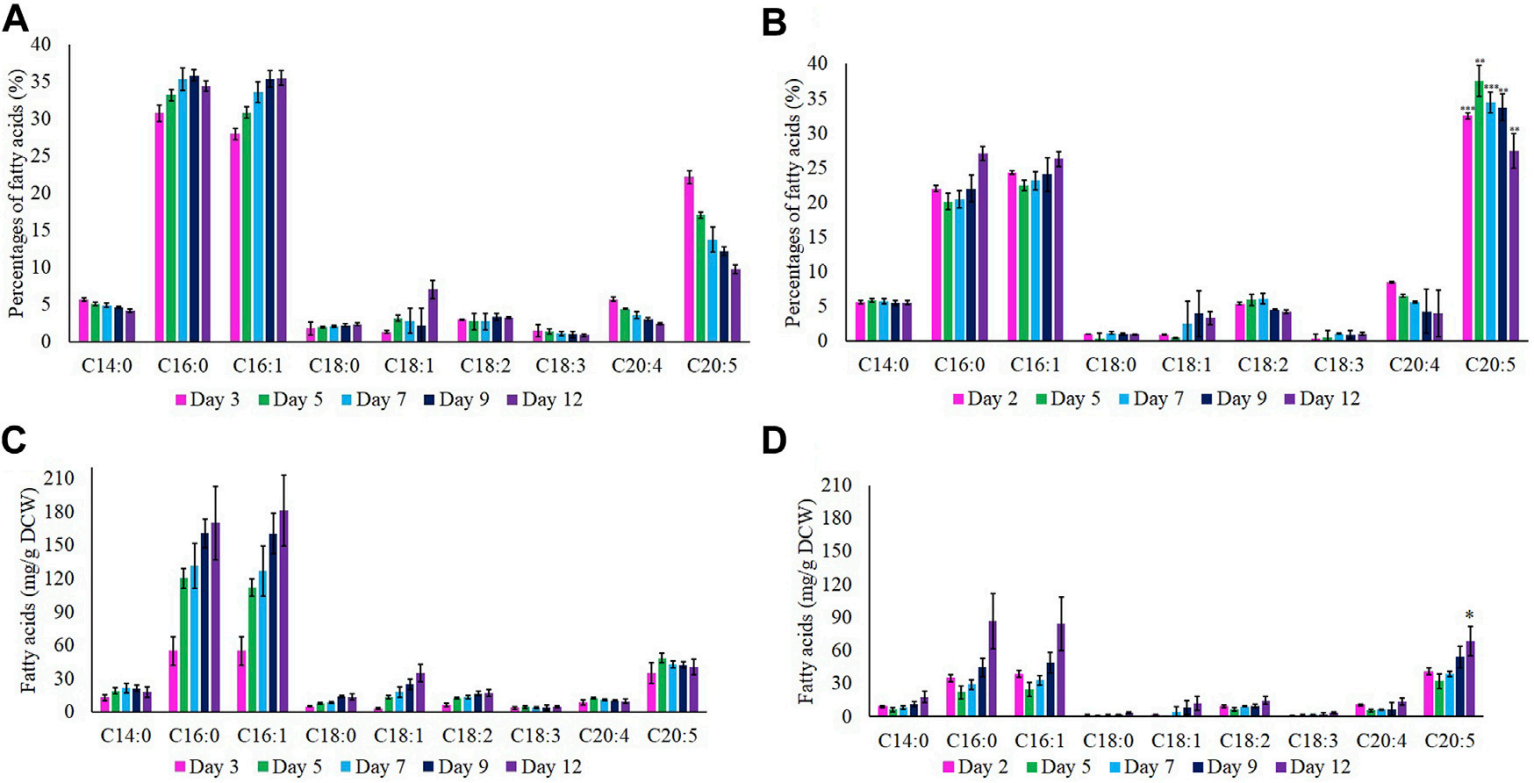
Percentages (%) of fatty acids at day 12. **(A)** Wild-type and **(B)** M1 mutant *N. oculata*. Quantification (mg/g) of fatty acids at day 12. **(C)** Wild-type and **(D)** M1 mutant *N. oculata*. Mean ± standard deviation is shown (n = 3) and t-tests determine the statistical significance (*p* < 0.05 [*]; *p* < 0.01 [**]; *p* < 0.001 [***]) for EPA content in the M1 mutant strain compared to the wild-type strain.

The compositions of FAME in wild-type and M1 mutant *N. oculata* cells were measured by GC-FID analysis. In terms of percentage composition (Figures 5A,B), the major FAME observed in both wild-type and M1 mutant strains were C16:0 (palmitic acid), C16:1 (palmitoleic acid), and C20:5 (EPA). The other FAMEs identified were C14:0 (myristic acid), C18:0 (stearic acid), C18:1 (oleic acid), C18:2 (linoleic acid), C18:3 (α/γ-linolenic-acid), and C20:4 (arachidonic acid/eicosatetraenoic acid). At the early exponential phase, the percentage of EPA was highest at 37.5 and 22.2% for M1 mutant and wild-type *N. oculata*, respectively (Figures 5A,B). This percentage of EPA in M1 mutant *N. oculata* was considerably higher than quantified at the mid-exponential phase in a previous *N. oceanica* study (Poliner et al., 2018). The higher percentage of EPA of TFA in total lipids at the early exponential phase in both strains suggested that EPA synthesis prefers favorable growth conditions. A previous study showed the chlorophyll *a* was directly proportional to MGDG quantity, where day 8 (exponential phase) had a higher chlorophyll *a* and MGDG quantity than day 12 in *N. salina* (Koh et al., 2019).

Total FAME amounts (mg/g DCW) were calculated by referring to the standard FAME intensity, as shown in Figures 5C,D. The EPA concentration increased gradually after day 2 and reached the highest (68.5 mg/g DCW) on day 12 for M1 mutant *N. oculata*. In contrast, the wild-type strain showed the highest EPA of 48.6 mg/g DCW on day 5. The EPA quantity recorded in M1 mutant was considerably higher than those in previous studies. Shi et al. (2021) recorded EPA at 40 and 45 mg/g DCW on day 4 for a wild-type *N. oceanica* strain and a Δ6 elongase overexpression strain, respectively. Yang et al. (2019a) described a Δ6 desaturase overexpression *N. oceanica* strain with 62.35 mg/g DCW on day 10, which decreased to around 50 mg/g DCW on day 13, while the wild-type strain had approximately 40 mg/g DCW on day 10, which increased to around 60 mg/g DCW on day 13.

An increase in neutral lipids (C16:0 and C16:1) was observed during batch growth in both strains and, as predicted, were inversely proportional to the nutrients level in the f/2 medium. The percentage of neutral lipids gradually increased for both wild-type and M1 mutant strain and reached the highest (35 and 27%, respectively) on day 12. Nutrient limitation is known to induce TAG accumulation as a stress response, and a previous study showed that C16:0 reached the maximum of around 40% of TFA under these conditions (Wei and Huang, 2017). In order to further investigate the increase in EPA content, two time-points were chosen based on the FAs profiles between TAG and EPA, EPA percentages, and absolute EPA quantifications. The two most significant time points were when the EPA content was measured at the highest and the lowest level compared to C16:0 and C16:1 components. Hence, the early exponential phase (days 2 and 3) and late exponential phase (day 12) were chosen as two-time points for LFQ proteomics analysis.

### LFQ Proteomic Overview

LFQ proteomics was conducted at two-time points, early and late exponential phase, in order to investigate differential protein expression patterns that could contribute to higher EPA synthesis in our M1 mutant *N. oculata* strain. Days 2 and 3 were selected for the early exponential phase in M1 mutant and wild-type *N. oculata*, respectively, while day 12 was selected for the late exponential phase.

MS/MS scans for LFQ experiments for wild-type and M1 mutant *N. oculata* in early exponential phase and end of exponential phase samples are shown in Supplementary Table S1. The UniProt proteome database (December 2020) contains only 219 proteins for *N. oculata*, although there are 15,363 proteins for *Nannochloropsis gaditana*. Hence, for this study, a *Nannochloropsis* genus proteome database was created by combining sequences from UniProt, NCBI, and MSPnr100 (Tran et al., 2016; Wang and Jia, 2020). The combined proteome database of the *Nannochloropsis* genus consisted of 16,270 protein sequences, and our identification process relied on matching identical peptide sequences.

**FIGURE 6:**
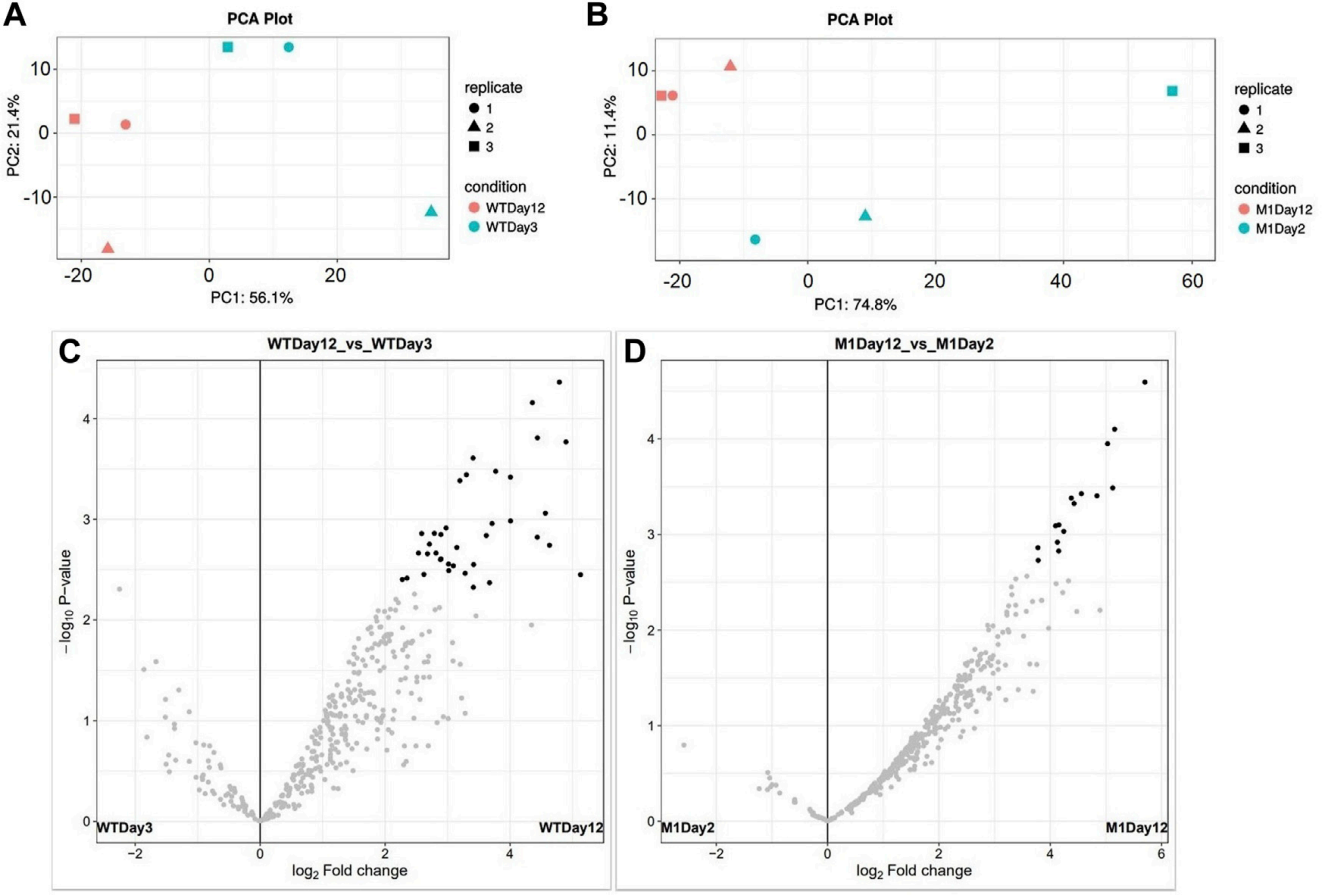
PCA plots show 12 samples clustered by biological replicates: **(A)** Wild-type *N. oculata* samples day 3 (light blue) and day 12 (pink) and **(B)** M1 mutant *N. oculata* samples day 2 (light blue) and day 12 (pink). Volcano plots show the significant protein distributions in wild-type **(C)** and M1 mutant **(D)** *N. oculata*.

A PCA plot for sample clustering and volcano plots displaying significantly differentially quantified proteins are shown in Figure 6. The number of protein groups with at least two peptides was 422 and 434 for wild-type and M1 mutant *N. oculata*, respectively. The numbers of significant proteins (> 2-fold changes and *p*-value < 0.05) were 123 and 103 for the wild-type and M1 mutant strain, respectively (Supplementary Tables S2, S3). Differential protein analyses will be discussed in relation to growth and stress, photosynthetic systems, FA, TAG and EPA synthesis, and membrane remodeling. The data were analyzed by comparing the early versus late exponential phase for the wild-type strain and subsequently the same switch in the growth phase in the mutant strain, following a discussion on how these differed.

### Photosynthetic System

Photosynthesis is the process where microalgae convert and store solar energy as energy-rich organic molecules as a source of energy for microalgae cell growth. Cell growth is associated with cell division and complex biochemical processes, including cell cycle machinery, cytoskeletal elements, chromosomes, and membranes (Kagıalı et al., 2017). In this study, cell division protein was a 10.4-fold increased in abundance in the M1 mutant strain from early to late exponential phase, while 7-fold upregulated in the wild-type strain. The higher cell division protein level in the M1 mutant could be related to faster cell division in the M1 mutant than in the wild-type strain. During autotrophic growth, microalgae cells utilize light energy harvested by chlorophyll molecules and convert carbon dioxide and water to carbohydrates and oxygen. Photosynthetic (rates) are different across microalgae species and culture conditions (Costache et al., 2013). The photosynthetic mechanism is organized in organelles, thylakoids, and stroma in chloroplasts. Different species of microalgae have specific preferences for the chlorophyll-binding group. For example, Chl *a/b*-binding proteins found in Viridiplantae (LHCA/LHCB), fucoxanthin Chl *a/c*-binding protein (FCP or LHCF) in diatoms, and LHCR in red algae (Carbonera et al., 2014). *N. oculata* has only chlorophyll *a* as a primary photosynthetic pigment and one plastid (Szabó et al., 2014). Maximizing the light absorption of light-harvesting antennae is a sustainable way to increase the growth rate and biomass production of microalgae cells (Szabó et al., 2014). Photosynthetic proteins in the M1 mutant were mostly upregulated from the early to late exponential phase, including photosystem I iron–sulfur center (22.3-fold increase), photosystem I subunit III (15.7-fold increase), chlorophyll A-B-binding protein (13.6-fold increase), photosystem II 12 kDa extrinsic protein (12.9-fold increase), photosystem II 11 kDa protein (12.4-fold increase), photosystem II CP43 reaction center protein (5.6-fold increase), chloroplast light-harvesting protein isoform 4 (5.5-fold increase), photosystem I reaction center subunit IV (5.2-fold increase), and photosystem I reaction center subunit XI (4.6-fold increase). The only photosynthetic protein found to increase significantly in the wild-type strain during the period of exponential phase was photosystem I reaction center subunit IV (3-fold increase). The results suggest that a faster photosynthetic efficiency was achieved in the M1 mutant. This increase in photosynthetic proteins led to the hypothesis that more NADPH could be available for reductive synthesis processes in the M1 mutant, hence contributing to the high efficiency of EPA synthesis. This is because the availability of NADPH has previously been shown to increase the reaction velocity of NADPH-requiring enzymes involved in FA synthesis such as acetyl-CoA carboxylase (ACCase) and ATP citrate lyase (ATP: CL) (Mühlroth et al., 2013). In *N. salina*, the increase in NADPH has recently been linked to higher FA synthesis (Jeon et al., 2021).

Rieske (2f3-2s) region protein and ferredoxin are common proteins involved in electron transfer chains in the mitochondria and chloroplast for NADPH generation (Fukuyama, 2004; Kameda et al., 2011). Rieske (2f3-2s) region protein was 52-fold upregulated, and ferredoxin was 29.7-fold upregulated in the M1 mutant strain, from early exponential to late exponential phase. However, Rieske (2f3-2s) region protein and ferredoxin were not significantly regulated in wild-type strain over the same growth phase.

### FA, TAG, and EPA Synthesis

**FIGURE 7:**
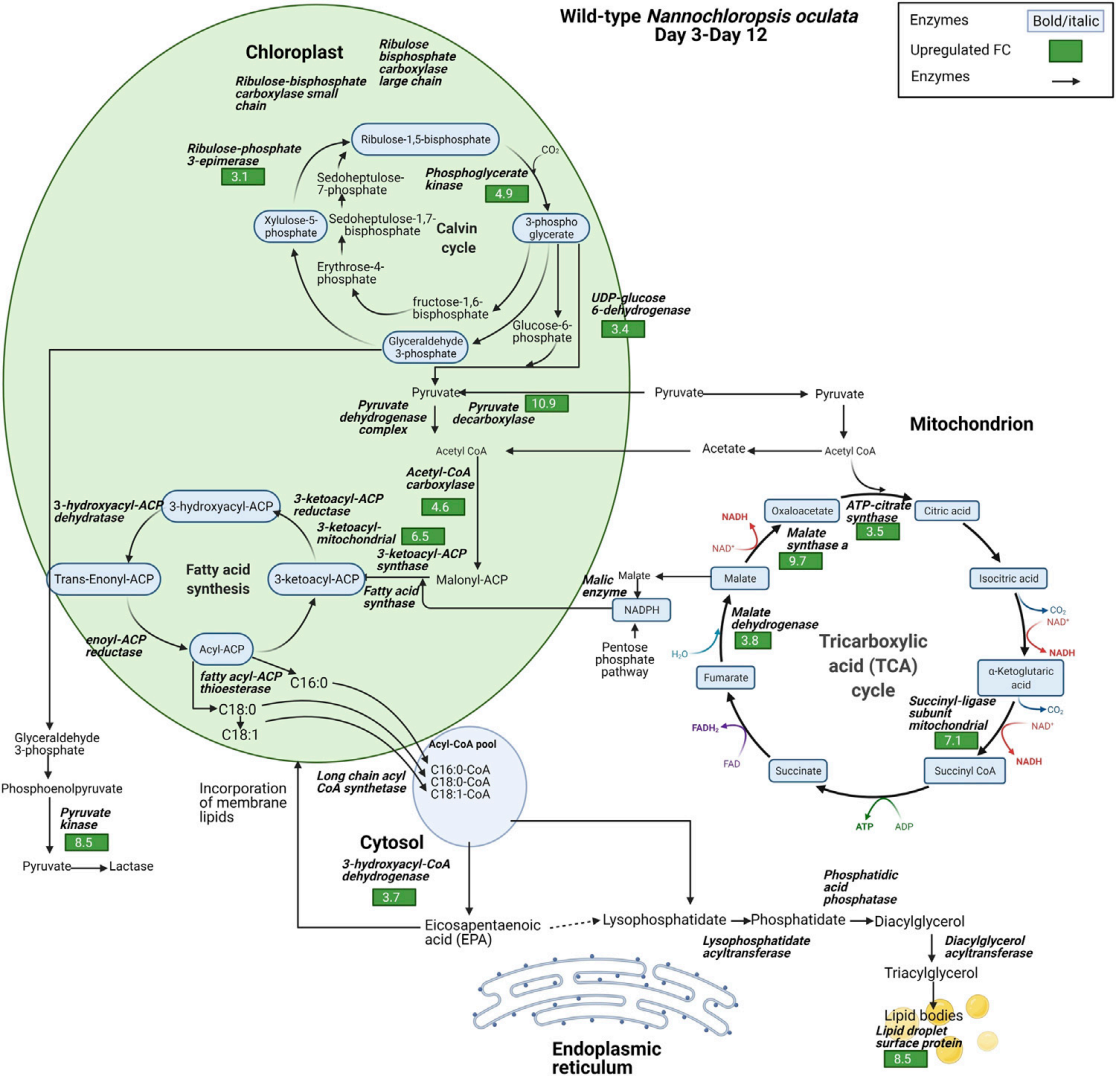
Diagram of enzyme regulations from day 3 to day 12 for carbon fixation toward TAG biosynthesis pathways for wild-type *N. oculata*. The diagram shows the pathways and their relation to fatty acid synthesis pathways. Significant quantified proteins are shown in the green and red boxes for upregulated and downregulated proteins, respectively.

**FIGURE 8:**
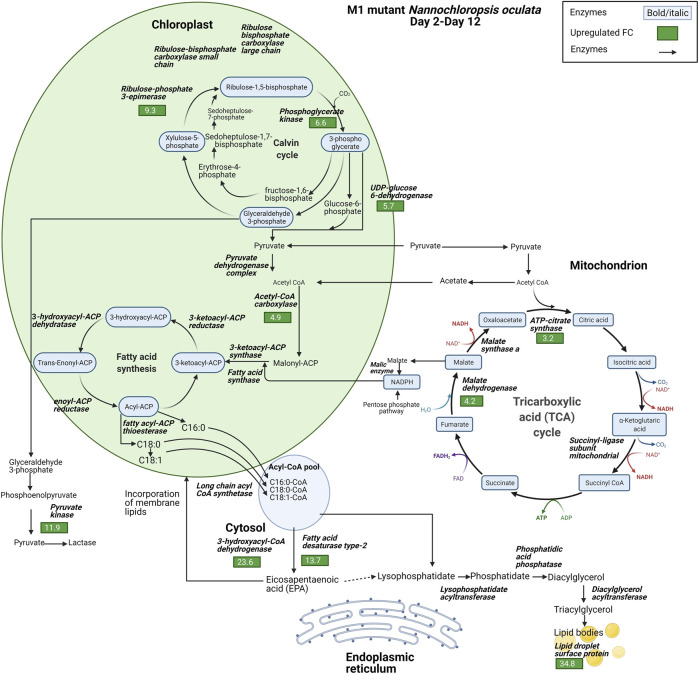
Diagram of enzyme regulations from day 2 to day 12 for carbon fixation toward TAG biosynthesis pathways for M1 mutant *N. oculata*. The diagram shows the pathways and their relation to fatty acid synthesis pathways. Significant quantified proteins are shown in the green and red boxes for upregulated and downregulated proteins, respectively.

The proteomic data highlighted the differences in the relative expression of key enzymes associated with the synthesis of FAs, TAG, and EPA. Lipid droplet surface protein (LDSP) was identified as one of the most differentially expressed proteins in both wild-type and M1 mutant *N. oculata*. LDSP are novel proteins associated with lipid droplets in *Nannochloropsis* sp., previously linked to the TAG storage compartment (Vieler et al., 2012). Moreover, lipid droplet structure is highly dynamic and involved in various cellular processes, such as regulation of energy homeostasis, remodeling of membranes and signaling (Zienkiewicz and Zienkiewicz, 2020). LDSP was 8.5-fold upregulated from day 3 to day 12 in wild-type *N. oculata* (Figure 7). However, LDPS was 34.8-fold upregulated in M1 mutant *N. oculata* from day 2 to day 12 (Figure 8). The increase in LDPS in wild-type cells was expected where the C16:0 quantity (mg/g DCW) had a 2.7-fold increase at the late exponential phase. This level of change was seen previously in *N. oculata* cells (Tran et al., 2016), where LDSP was 2.4-fold upregulated after 11 days of cultivation. However, the much larger fold change LDSP in the M1 mutant strain implies a major alteration in cell regulation that leads to an increase in FAs.

In contrast, acyl-coenzyme A dehydrogenase (ACAD) had a 35-fold increase in the wild-type strain compared to a smaller 9.2-fold increase in the M1 mutant. ACAD is responsible for FA beta-oxidation in mitochondria (Tan and Lee, 2016), and therefore a reduced rate of FA degradation in the mutant relative to the wild-type appears probable. Acetyl CoA carboxylase (ACCase) converts acetyl-CoA to malonyl-CoA and serves as a carbon donor for FA chain extension in the plastid (Li et al., 2014). 3-hydroxyacyl-CoA dehydrogenase (HCDH) is involved in FA metabolism and catalyzes the reduction of 3-hydroxyacyl-CoA to 3-oxoacyl-CoA. Short- and medium-chain HCDH reside in the mitochondrial matrix, while long-chain HCDH is part of the membrane-associated multifunction protein in the mitochondria and peroxisome (Xu et al., 2014). HCDH had a 23.6-fold increase in the M1 mutant, significantly higher than the increase observed in wild-type *N. oculata* (3.7-fold). Although several enzymes are involved in FA elongation, for example, 3-oxoacyl-reductase, 3-hydroacyl-CoA dehydratase and enoyl-CoA reductase (process palmitic acid (C16:0) to stearic acid (C18:0)) (Kapase et al., 2018), these proteins were not detected here.

Fatty acid desaturase type 2 (FAD2) was 13.7-fold upregulated in the M1 mutant strain, whereas it was not significantly regulated in the wild-type strain. FAD2 enzyme is part of integral membrane protein in ER, responsible for the biological switch from oleic acid (C18:1) to linoleic acid (C18:2) (Dar et al., 2017). This finding suggests that FAD2 protein may be actively involved in EPA synthesis in the ER. On the contrary, 3-ketoacyl-mitochondrial was 6.5-fold upregulated in wild-type and not differentially expressed in the M1 mutant. With reference to the UniProt database and gene ontology functions, 3-ketoacyl-CoA thiolase and 3-ketoacyl-mitochondrial share a similar function that enables acetyl-CoA C-acyltransferase activity. 3-ketoacyl-CoA thiolase enzyme is involved in FA beta-oxidation, whereas acetyl-CoA is catalyzed in the chloroplast for FA synthesis (Osumi et al., 1992; Kechasov et al., 2020). Another protein with a potential role is 3-ketoacyl-ACP synthase (KAS), an important enzyme involved in FA elongation in plastids (Chaturvedi and Fujita, 2006; Morales-Sánchez et al., 2016).

Three possible spatial routes have been previously suggested for EPA synthesis (Mühlroth et al., 2013): 1) chloroplast → acetyl-CoA → ER → membrane lipids, 2) chloroplast → mitochondrion → acetyl-CoA → ER → membrane lipids, and 3) chloroplast → citrate → acetyl-CoA → ER → membrane lipids. Due to the nature of proteomics data with missing proteins, it is difficult to confirm which route is likely for *N. oculata* wild-type and M1 mutant cells. However, based on differential protein expression, it is possible that wild-type *N. oculata* undertook the first or second route when 3-ketoacyl-mitochondrial was 6.5-fold upregulated in the chloroplast–mitochondria pathway. In the M1 mutant, EPA synthesis may be maximized outside the chloroplast; in the cytosol and ER. The EPA may be incorporated into membrane lipids during the exponential phase. The highest EPA was quantified on day 12 with 68.5 mg/g DCW, suggesting that EPA could be translocated from membrane lipids to TAG at the end exponential phase period.

### Membrane Lipid Remodeling

Lipidomic analyses have shown increased accumulation of neutral lipids during nitrogen-deprived conditions, in addition to a decrease in the level of membrane lipids (Han et al., 2017; Liang et al., 2019). This implies that cellular responses responsible for TAG accumulation are related to the modification of membrane lipids. Under nitrogen constraint, MGDG was the predominant membrane lipid component that was reduced in *N. oceanica*, which was proposed as a protective mechanism to prevent the degradation of the thylakoid and chloroplast envelope membranes (Han et al., 2017). The membrane lipid composition was also remodeled under phosphate starvation in the same strain, where phospholipids were replaced by betaine lipids (Murakami et al., 2020). Our methodology of combined mutagenesis and selection with galvestine-1 also remodeled membrane lipids in *N. oculata*. A high level of LDSP expression in M1 mutant *N. oculata* cells suggests that the membrane lipids were modified and converted to TAG at significantly higher levels, and hence EPA that is usually enriched in membrane lipids were transported to TAG. Both biomass growth and FA synthesis compete for the same substrates, acetyl-CoA and NADPH; hence substrate availability is a rate-limiting step in balancing growth rate and FA accumulation (Tan and Lee, 2016). Acetyl-CoA conversion is derived from the glycolytic process, and pyruvate kinase (PK) and enolase are identified as primary photosynthate (Tran et al., 2016). Carbohydrates in the form of pyruvate are converted to acetyl-CoA to supply the cell with energy and reduced carbon (Mühlroth et al., 2013). Acetyl-CoA also is a key metabolite in both the TCA cycle in the mitochondrion and FA synthesis in the chloroplast (Mühlroth et al., 2013). Phosphoglucomutase is involved in chrysolaminarin synthesis and functions as a critical node in sharing the carbon precursors between carbohydrate and lipid metabolism (Yang Y.-F. et al., 2019) and had an 11.5-fold increase in the M1 mutant cells, whereas it was only 4-fold upregulated in the wild-type strain. A higher carbohydrate metabolic process in M1 mutant might suggest a higher quantity of membrane lipids is present in M1 mutant cells, which could be used to facilitate higher EPA quantities, than in the wild-type strain.

Identifying protein changes that contribute to membrane lipid composition remains challenging because a relatively small number of membrane proteins have been sequenced and studied in algae (Garibay-Hernández et al., 2017). MGDG and DGDG are the major lipids of the photosynthetic membrane, where MGDG is synthesized in the chloroplast (Dolch et al., 2017), and DGDG is synthesized in both chloroplast and ER (Cecchin et al., 2020). The formation of membrane polar lipids is intrinsically linked to photosynthesis, for example, there is evidence to suggest that the light-harvesting complex may stabilize the MGDG component in thylakoids membranes (Han et al., 2017). A greater membrane lipid content in M1 mutant could be due to more efficient light-harvesting within photosynthesis. As a result, during the early exponential phase growth, more EPA might be produced. When the mutant cells approached the end of the exponential phase, the lower MGDG led to more EPA being translocated outside the membrane lipid, resulting in a greater overall EPA level. The cell wall architecture of *N. oculata* is primarily composed of polysaccharides, with 68% as glucose subunits (Scholz et al., 2014). Through glycolysis, glucose sourced from the cell wall can be oxidized to generate energy that can be diverted to FA synthesis. In a recent study, during nitrogen starvation, the *N. oceanica* cell wall altered from two layers with a thickness of 32.9 nm to a one-layer cell wall with a thickness of 37.8 nm (Roncaglia et al., 2021). This implied that the cell wall degradation might also contribute to FA synthesis.

### Cellular Location of EPA

**FIGURE 9:**
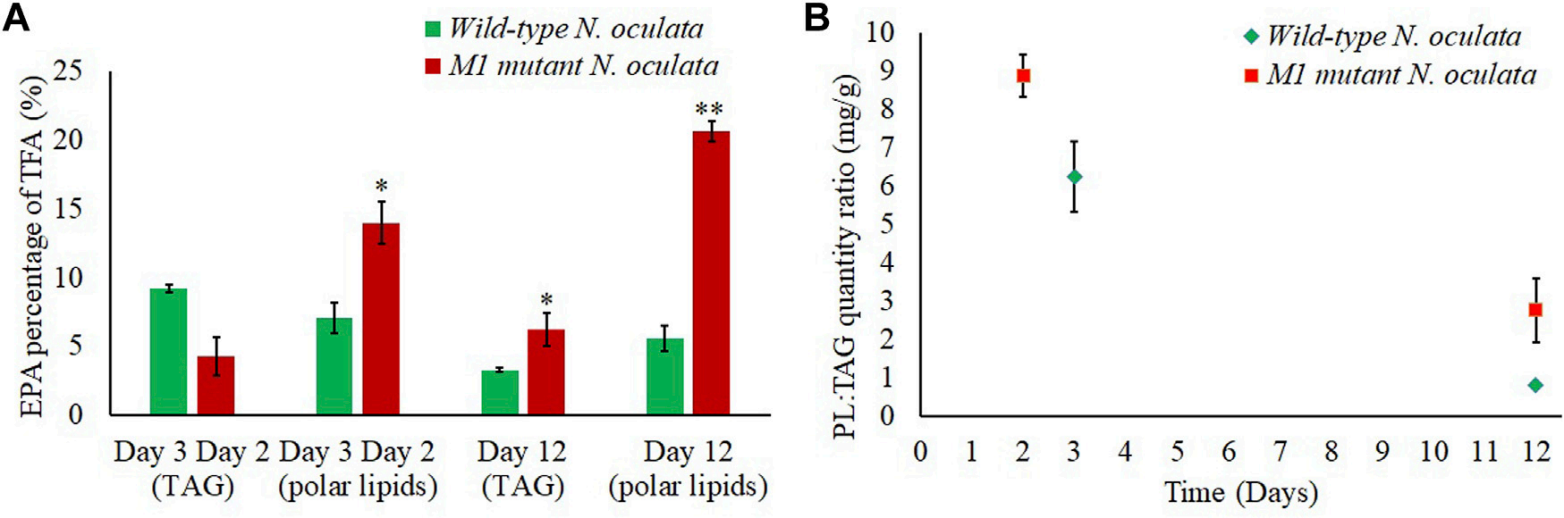
Fatty acid content. **(A)** EPA percentages (%) of TFA in polar lipids and TAG at day 2 (M1 mutant), day 3 (wild-type), and day 12 (wild-type and M1 mutant) *N. oculata*. **(B)** Polar lipid (PL):TAG quantity ratio (mg/g) at day 2 (M1 mutant), day 3 (wild-type), and day 12 (wild-type and M1 mutant) *N. oculata*. Mean ± standard deviation is shown (n = 3) and t-tests determine the statistical significance (*p* < 0.05 [*]; *p* < 0.01 [**]; *p* < 0.001 [***]) for EPA content in the M1 mutant strain compared to the wild-type strain.

The absolute quantity and percentage composition of FAME were also investigated within the TAG and polar lipid cellular components, as the proteomic data implied the EPA in M1 mutant *N. oculata* could be translocated outside the chloroplast. The ratio of polar membrane lipids to TAG (Figure 9B) decreased on day 12, indicating that TAG was accumulated in both wild-type and M1 mutant strains at the end of 12 days of culturing. In the M1 mutant, the EPA percentage in TAG was 1.9-fold higher than the wild-type strain (Figure 9A). This provided additional evidence for the enhanced translocation of EPA to TAG in the M1 mutant strain and could be linked to the elevated abundance of LDSP in this strain. The EPA percentage in M1 mutant was 2-fold and 3.7-fold increased at the early and late exponential phase, respectively, compared to the wild-type strain. The enhanced EPA quantity in the M1 mutant is likely linked to the relatively high synthesis of polar lipids, particularly MGDG. A high MGDG content was preserved until the late exponential phase and could be due to the abundance of polar lipids synthesized in the M1 mutant compared to the wild-type strain. Hence, the EPA could be partially translocated to TAG at day 12 in M1 mutant, while in the wild-type strain, the EPA and MGDG could be converted to TAG, resulting in a lower EPA. In the FAs pathways, membrane lipids were previously observed to translocate to TAG, especially under limited nutrient conditions (Janssen et al., 2020). Hence, the EPA could be translocated with membrane lipids to TAG with a structural modification to saturated FA. On the contrary, the EPA could be directly translocated to TAG from the ER (Ma et al., 2016); however, this mechanism is less studied in the literature.

## Conclusion

A novel strategy to increase EPA productivity in *N. oculata* was devised using a combination of EMS-induced random mutagenesis and screening with FAS chemical inhibitors, which have not been applied together previously. LFQ proteomic analysis was conducted and highlighted metabolic pathways that could contribute to enhance EPA synthesis and alternative translocation routes between a selected mutant strain and the wild-type strain. Overall, the developed method could be used as an alternative to genetic engineering methods for increasing EPA production, although cell engineering targets were highlighted for further improvement studies. Increasing EPA productivity in industrially relevant microalgal strains increases the sustainable manufacturing of this LC-PUFA.

## Data Availability

The original contributions presented in the study are included in the article/Supplementary Material, further inquiries can be directed to the corresponding author.
