# Supplementary material for: Comparative Proteomics Reveals Evidence of Enhanced EPA Trafficking in a Mutant Strain of Nannochloropsis oculata
Source: Front Bioeng Biotechnol. 2022 May 12;10:838445. doi: 10.3389/fbioe.2022.838445 (PMC9134194; doi:10.3389/fbioe.2022.838445)
Supplement: Supplementary file 1 [file DataSheet1.pdf]

Supplementary Material

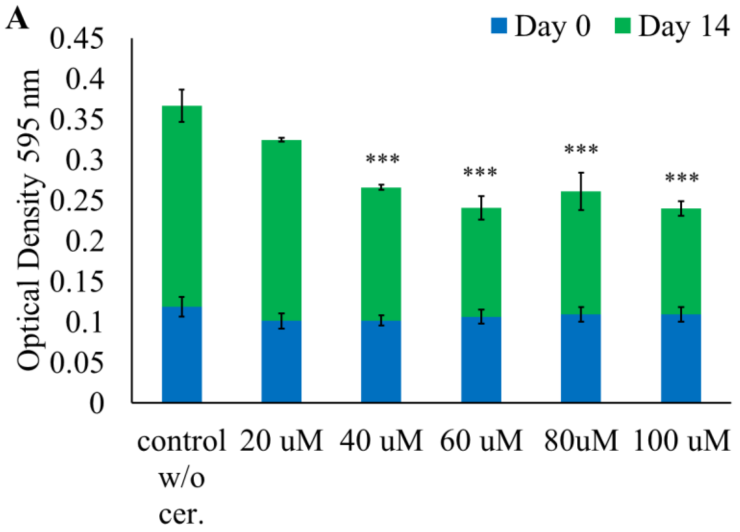

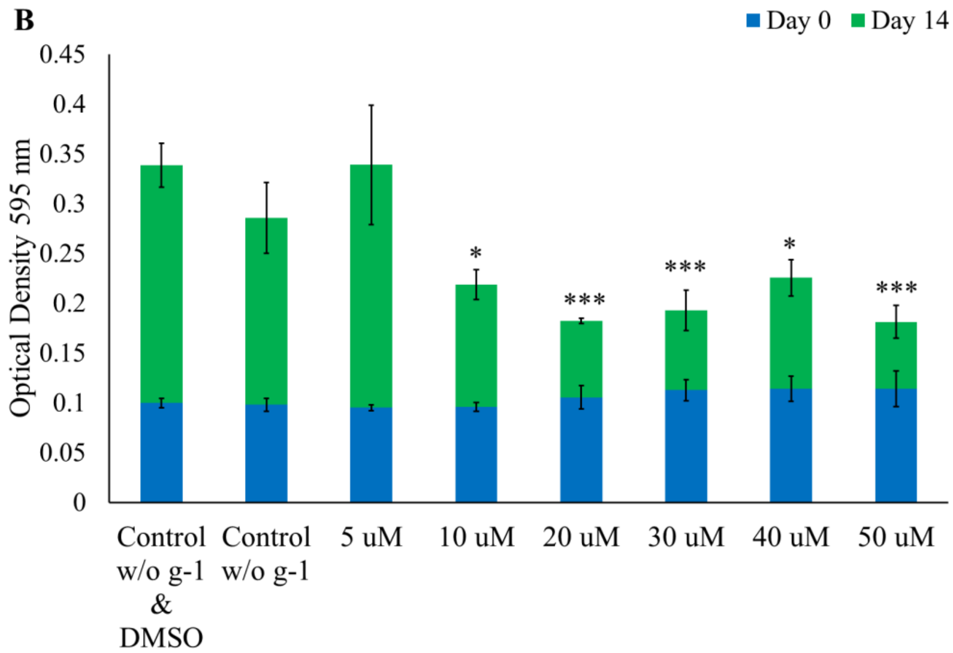

Supplementary Figure 1: (A) Growth of wild-type *N. oculata* in the presence of different concentrations of fatty acid synthase inhibitor, cerulenin. **(B)** Growth of wild-type *N. oculata* in the presence of different concentrations of MGDG synthase inhibitor, galvestine-1. Optical densities were measured at 595 nm by a plate reader (TECAN, Germany). Vertical bars are the standard deviation of the means. Mean ± standard deviation is shown (n = 6) and t-tests determine statistical significance ($p < 0.05$ [*]; $p < 0.01$ [**]; $p < 0.001$ [***]).

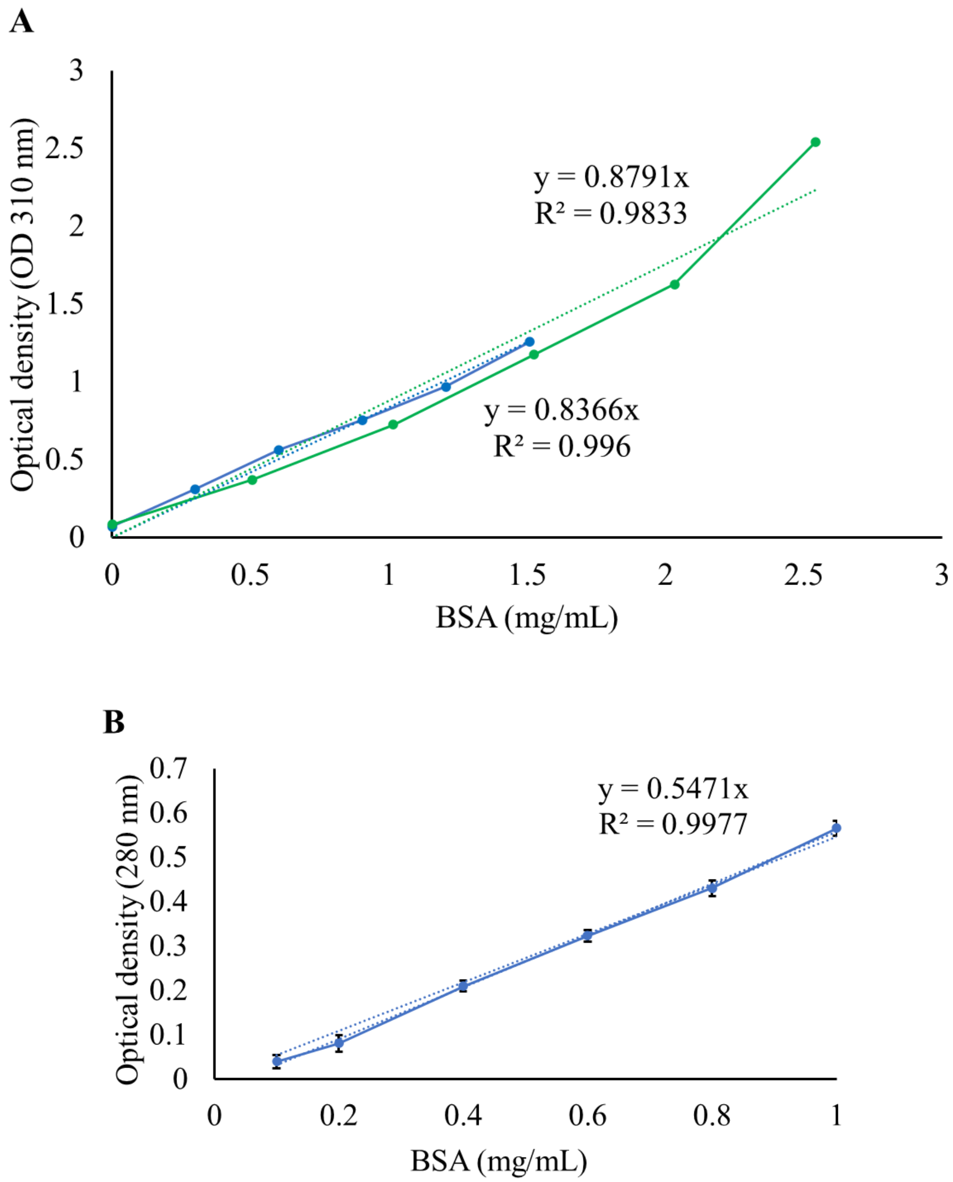

Supplementary Figure 2: (A) Bovine serum albumin (BSA) standard curve ranged from 0 to 2.6 mg/mL using the microbiuret method (Collos et al., 1999) and optical density was measured at 310 nm using a quartz cuvette. **(B)** Bovine serum albumin (BSA) standard curve ranged from 0 to 1.0 mg/mL using the Nanodrop 2000 method (Thermo Fisher Scientific, United Kingdom).

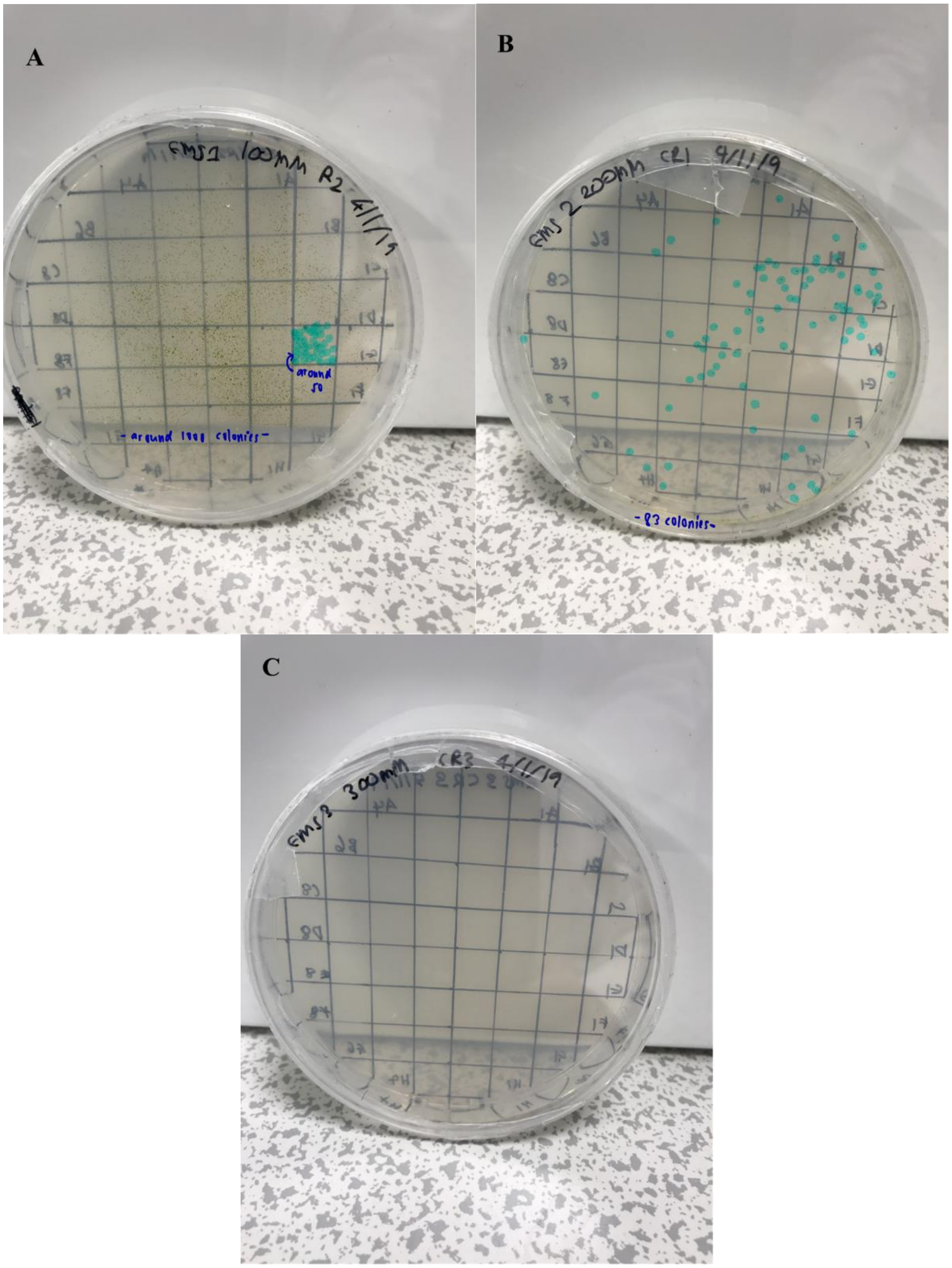

Supplementary Figure 3: *N. oculata* mutant colonies of **(A)** 100 mM, **(B)** 200 mM, and **(C)** 300 mM EMS were grown on the f/2 medium agar containing 50 µM cerulenin after 3 weeks of incubation at 130 µmol $m^{-2}s^{-1}$, 20 °C, and 12h:12h (light: dark) cycle. Approximately more than 1000, 82, and no colonies were presented on the plate **(A)**, **(B)**, and **(C)**, respectively, after 3 weeks of incubation.

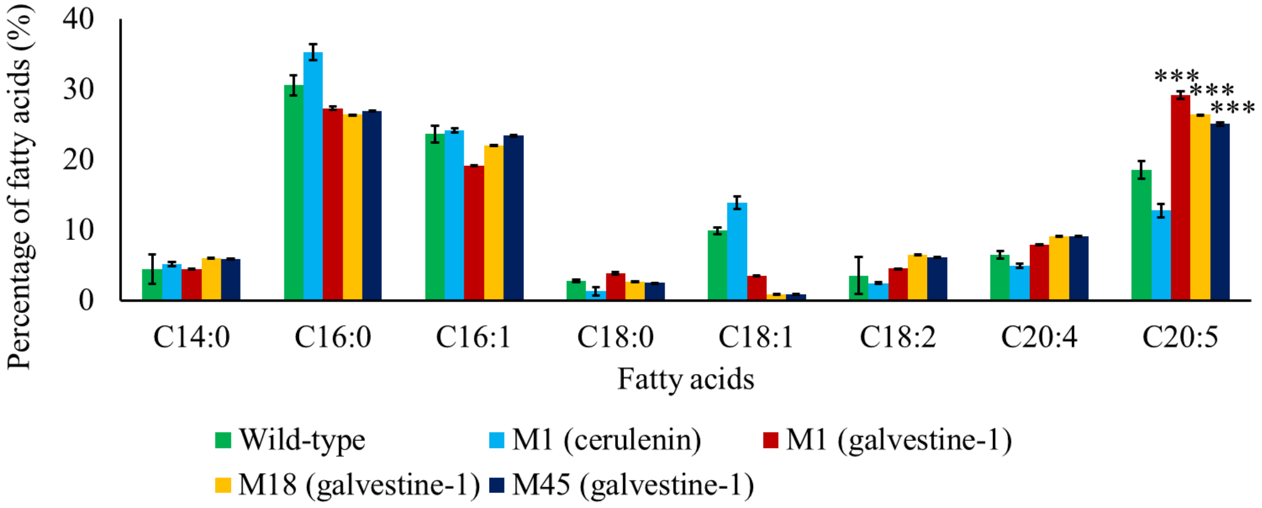

Supplementary Figure 4: Comparative analysis of percentage (%) changes in the fatty acid composition of TFA in wild-type, M1 mutant (treated with 60 µM cerulenin), M1, M18, and M45 (treated with 10 µM galvestine-1) *N. oculata* at exponential growth phase cells having optical densities of 0.8, measured at 595 nm. Mean ± standard deviation is shown (n = 3) and t-tests determine statistical significance ($p < 0.05$ [*]; $p < 0.01$ [**]; $p < 0.001$ [***]).

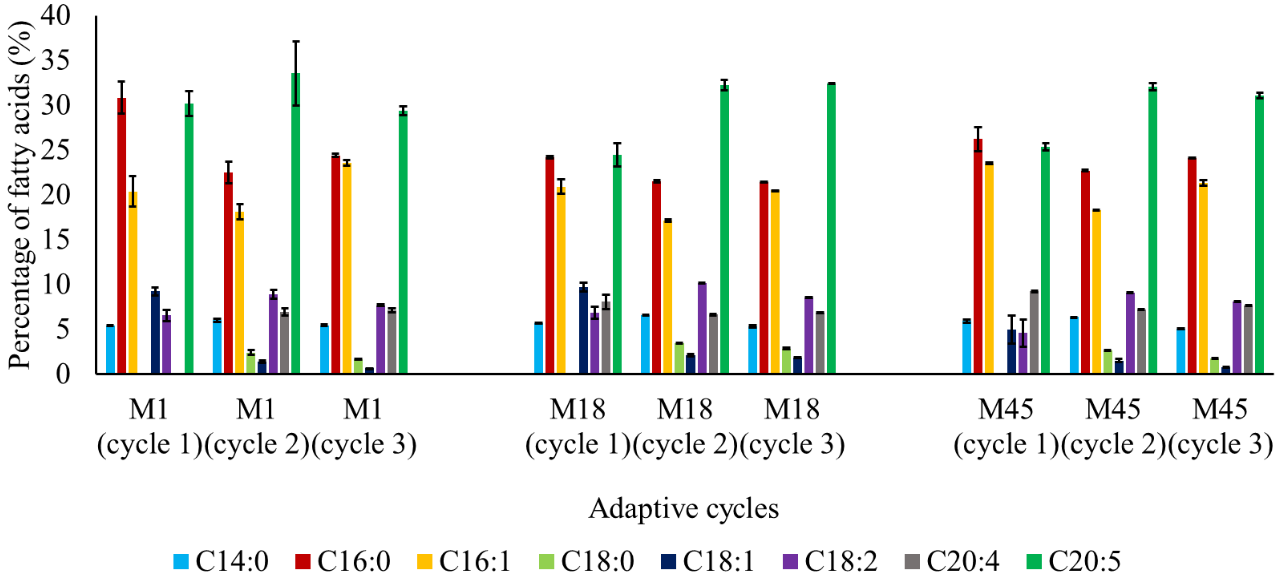

Supplementary Figure 5: Percentage of primary fatty acids of TFA comparison for M1, M18, and M45 *N. oculata* mutants over 3 cycles adaptation in f/2 medium containing 10 µM galvestine-1. The experiments were carried out in sterile cell culture flasks (Nunc™, Thermo Fisher Scientific, United Kingdom) at 130 µmol $m^{-2}s^{-1}$, 20 °C, and 12 h:12 h (light: dark) cycle, 160 RPM shaking for 24 days (8 days for 1 cycle).

Supplementary Table 1. MS/MS scans for triplicates samples for wild-type and M1 mutant *N. oculata* at two-time points early exponential phase 2 and 3, and end of exponential phase day 12. Descriptions: wild-type (WT), M1 mutant (M1), Day 2 (D2), Day 3 (D3), Day 12 (D12), Flask 1 (F1), Flask 2 (F2), Flask 3 (F3).

Samples	WTD3F1	WTD3F2	WTD3F3	WTD12F1	WTD12F2	WTD12F3
MS/MS scans	45958	38178	53645	54967	47963	63726
Samples	M1D2F1	M1D2F2	M1D2F3	M1D12F1	M1D12F2	M1D12F3
MS/MS scans	41802	31463	19002	43942	37755	45303

Supplementary Table 2. Label-free quantification (LFQ) analyst results for Wild-type *N. oculata* Day 3 vs Day 12 samples.

Gene Name	*Protein IDs*	*WTDay12_vs_WTDay3_log2 fold change*	*WTDay12_vs_WTDay3_p.val*	*WTDay12_vs_WTDay3_p.adj*	*significant*	*WTDay12_vs_WTDay3_significant*	*imputed*	*num_NAs*	*Protein names*
AIU44072.1	AIU44072.1	2.35	0.00384	0.0437	TRUE	TRUE	TRUE	4	methylmalonyl CoA mutase
Arf1	tr|W7TL63|Arf1	3.02	0.00324	0.0416	TRUE	TRUE	FALSE	0	Adp-ribosylation factor
CAT	tr|W7T2K9|CAT	3.41	0.00474	0.0496	TRUE	TRUE	TRUE	2	Catalase
DKC1	tr|K8Z676|DKC1	3.3	0.000361	0.0178	TRUE	TRUE	TRUE	3	H/ACA ribonucleoprotein complex subunit 4
GCSL.1	tr|W7U9J4|GCSL	4.89	0.000171	0.0165	TRUE	TRUE	TRUE	3	Dihydrolipoyl dehydrogenase
gi|553180870|ref|XP_005853474.1|	gi|553180870|ref|XP_005853474.1|	2.79	0.00138	0.0324	TRUE	TRUE	TRUE	1	6-phosphofructo-2-kinase / fructose-2,6-bisphosphatase
GSR	tr|W7U997|GSR	3.2	0.000414	0.0178	TRUE	TRUE	TRUE	3	Glutathione reductase
Naga_100002g147	tr|W7TNH0|Naga_100002g147	3.15	0.00191	0.0351	TRUE	TRUE	TRUE	1	Pyruvate carboxylase
Naga_100002g172	tr|W7TNX9|Naga_100002g172	5.13	0.00355	0.0416	TRUE	TRUE	TRUE	2	Acyl-dehydrogenase

Naga_100007g50	tr|W7U8D0|Naga_100007g50	3.71	0.0011	0.0324	TRUE	TRUE	TRUE	2	Protein kinase, ATP binding site
Naga_100010g85	tr|W7TMD6|Naga_100010g85	4.63	0.00181	0.035	TRUE	TRUE	TRUE	2	Mitochondrial phosphate transporter
Naga_100013g96	tr|W7UCP4|Naga_100013g96	3.28	0.00344	0.0416	TRUE	TRUE	TRUE	1	Malate synthase a
Naga_100014g18	tr|W7TRI5|Naga_100014g18	2.28	0.00396	0.0438	TRUE	TRUE	FALSE	0	Phosphoglycerate kinase
Naga_100016g72	tr|W7TXQ6|Naga_100016g72	2.53	0.00217	0.0355	TRUE	TRUE	FALSE	0	Peptidyl-prolyl cis-trans isomerase
Naga_100023g44	tr|W7TQJ5|Naga_100023g44	2.98	0.00122	0.0324	TRUE	TRUE	TRUE	4	Chaperonin containing tcp1 theta subunit
Naga_100024g37	tr|W7U814|Naga_100024g37	4.56	0.00087	0.0324	TRUE	TRUE	TRUE	2	Succinate dehydrogenase flavoprotein subunit
Naga_100024g60	tr|W7TSA3|Naga_100024g60	2.62	0.00352	0.0416	TRUE	TRUE	TRUE	2	Rab11 family gtpase
Naga_100025g12	tr|W7TRS4|Naga_100025g12	3.77	0.000334	0.0178	TRUE	TRUE	TRUE	3	Isocitrate lyase
Naga_100025g3	tr|W7U0R9|Naga_100025g3	2.89	0.00248	0.0374	TRUE	TRUE	TRUE	3	Importin subunit alpha

Naga_100042g43	tr|W7U8W3|Naga_100042g43	2.71	0.00177	0.035	TRUE	TRUE	FALSE	0	Nadp-dependent glyceraldehyde-3-phosphate dehydrogenase
Naga_100048g7	tr|W7TZM5|Naga_100048g7	4.44	0.000155	0.0165	TRUE	TRUE	TRUE	3	Aldehyde dehydrogenase
Naga_100049g35	tr|W7TDK1|Naga_100049g35	4.44	0.00151	0.0324	TRUE	TRUE	TRUE	1	Alcohol dehydrogenase
Naga_100051g28	tr|W7TM97|Naga_100051g28	2.58	0.00138	0.0324	TRUE	TRUE	TRUE	3	Cytochrome c1 heme protein
Naga_100084g10	tr|W7U0X5|Naga_100084g10	2.81	0.00216	0.0355	TRUE	TRUE	TRUE	2	Luminal binding protein
Naga_100106g2	tr|W7TUQ1|Naga_100106g2	4.01	0.000381	0.0178	TRUE	TRUE	TRUE	3	40S ribosomal protein S4
Naga_100122g12	tr|W7TWJ4|Naga_100122g12	3.41	0.000247	0.0178	TRUE	TRUE	TRUE	2	Cupin, RmlC-type
Naga_100177g17	tr|W7U719|Naga_100177g17	3.67	0.00426	0.0458	TRUE	TRUE	FALSE	0	Uncharacterized protein
Naga_100197g3	tr|W7TLB7|Naga_100197g3	3.09	0.0029	0.0387	TRUE	TRUE	TRUE	1	Pyruvate kinase
Naga_100199g4	tr|W7TNY2|Naga_100199g4	3.62	0.00145	0.0324	TRUE	TRUE	TRUE	3	Epimerase 4-reductase

Naga_100597g2	tr|W7T6X8|Naga_100597g2	2.89	0.00142	0.0324	TRUE	TRUE	TRUE	3	Lactoylglutathione lyase
Naga_100744g1	gi|553190853|ref|XP_005855276.1|	4.01	0.00104	0.0324	TRUE	TRUE	TRUE	1	wos2 protein
NGATSA_3001800	tr|I2CPF4|NGATSA_3001800	4.36	6.95E-05	0.0135	TRUE	TRUE	TRUE	3	Rnp domain-containing protein
NGATSA_3003400	tr|I2CNZ7|NGATSA_3003400	2.68	0.0022	0.0355	TRUE	TRUE	TRUE	3	Glutamate synthase (NADPH/NADH) small chain
NGATSA_3005800	tr|I2CQR9|NGATSA_3005800	3.01	0.00278	0.0387	TRUE	TRUE	TRUE	3	GMP synthase (Glutamine-hydrolysing)
PRX2	tr|W7TZN7|PRX2	4.79	4.35E-05	0.0135	TRUE	TRUE	TRUE	2	Thioredoxin-dependent peroxide reductase
SHMT2	tr|W7TN09|SHMT2	3.42	0.00282	0.0387	TRUE	TRUE	TRUE	2	Serine hydroxymethyltransferase
tr|I2CPE6|NGATSA_2003000	tr|I2CPE6|NGATSA_2003000	2.89	0.00251	0.0374	TRUE	TRUE	TRUE	1	Uncharacterized protein
AAB94637.1	AAB94637.1	2.13	0.0967	0.208	FALSE	FALSE	FALSE	0	violaxanthin/chlorophyll a binding protein precursor
AAT	tr|W7U3D6|AAT	2.15	0.0232	0.102	FALSE	FALSE	TRUE	4	Aspartate aminotransferase
AFB75402.1	AFB75402.1	3.08	0.0168	0.087	FALSE	FALSE	FALSE	0	lipid droplet surface protein

AFJ68664.1	AFJ68664.1	2.25	0.0394	0.136	FALSE	FALSE	TRUE	3	endo-b- -glucanase, partial
AFJ69274.1	AFJ69274.1	-1.03	0.364	0.49	FALSE	FALSE	TRUE	3	Ran-binding protein 1, partial
AFJ69311.1	AFJ69311.1	1.13	0.197	0.325	FALSE	FALSE	FALSE	0	rRNA 2-O-methyltransferase fibrillarin, partial
ANT70525.1	ANT70525.1	1.22	0.164	0.292	FALSE	FALSE	TRUE	1	violaxanthin de-epoxidase
AP1B1	tr|K8YNR1|AP1B1	2.09	0.00783	0.0663	FALSE	FALSE	TRUE	2	AP complex subunit beta
ASS	tr|W7U626|ASS	2.34	0.253	0.389	FALSE	FALSE	TRUE	2	Argininosuccinate synthase
atp1	tr|T1R8F9|atp1	0.961	0.13	0.25	FALSE	FALSE	FALSE	0	ATP synthase subunit alpha
atp8	tr|T1R7J2|atp8	0.841	0.364	0.49	FALSE	FALSE	FALSE	0	ATP synthase F0 subunit 8
atpA	tr|T1RIM9|atpA	-0.485	0.448	0.577	FALSE	FALSE	FALSE	0	ATP synthase subunit alpha, chloroplastic
atpB	tr|T1RJ81|rpl13	-0.511	0.532	0.634	FALSE	FALSE	TRUE	4	50S ribosomal protein L13, chloroplastic
atpB.1	tr|T1RHE4|atpB	-0.132	0.806	0.859	FALSE	FALSE	FALSE	0	ATP synthase subunit beta, chloroplastic
atpB.2	tr|T1RJP7|psaA	0.519	0.369	0.493	FALSE	FALSE	FALSE	0	Photosystem I P700 chlorophyll a apoprotein A1
atpD	tr|T1RJB4|atpD	-0.407	0.497	0.609	FALSE	FALSE	FALSE	0	ATP synthase CF1 delta chain

atpE	tr|T1RJM9|atpE	-0.911	0.345	0.47	FALSE	FALSE	FALSE	0	ATP synthase epsilon chain, chloroplastic
ATPEF1O	tr|K8YVX1|ATPEF1O	1.35	0.0703	0.184	FALSE	FALSE	FALSE	0	H+-transporting ATPase oligomycin sensitivity conferral protein
atpF	tr|T1RIU1|atpF	-0.902	0.174	0.302	FALSE	FALSE	FALSE	0	CF0 subunit I of ATP synthase
atpG	tr|T1RIS0|atpG	-0.808	0.178	0.303	FALSE	FALSE	FALSE	0	F0F1 ATP synthase subunit B
cbbX	tr|W7TPW8|cbbX	2.03	0.196	0.325	FALSE	FALSE	TRUE	1	Rubisco expression protein
cbbX.1	tr|T1RJ59|cbbX	0.42	0.474	0.594	FALSE	FALSE	FALSE	0	Putative rubisco expression protein
CCT3	tr|W7TWK9|CCT3	0.987	0.219	0.353	FALSE	FALSE	TRUE	4	T-complex protein 1 subunit gamma
clpC	tr|T1RJA6|clpC-I	0.621	0.456	0.585	FALSE	FALSE	FALSE	0	ATP-dependent Clp protease ATPase subunit
clpC-II	tr|T1RJR9|clpC-II	1.94	0.0951	0.206	FALSE	FALSE	TRUE	1	ATP-dependent Clp protease
clpC-II .1	tr|T1RJA1|clpC-II	-1.67	0.0259	0.105	FALSE	FALSE	TRUE	2	ATP-dependent Clp protease
clpP	tr|W7TMY3|clpP	1.12	0.0716	0.186	FALSE	FALSE	FALSE	0	ATP-dependent Clp protease proteolytic subunit
CPS	tr|W7U5E4|CPS	2.32	0.0379	0.132	FALSE	FALSE	FALSE	0	Carbamoyl-phosphate synthase

CYN	tr|W7TQJ8|CYN	3.2	0.0276	0.108	FALSE	FALSE	TRUE	1	Peptidyl-prolyl cis-trans isomerase
DAPAT	tr|W7TK41|DAPAT	0.0615	0.94	0.96	FALSE	FALSE	TRUE	1	Ll-diaminopimelate aminotransferase
dbj	gi|224809175|dbj|BAH28795.1|	0.279	0.663	0.748	FALSE	FALSE	FALSE	0	glyceraldehyde-3-phosphate dehydrogenase
dnaK	tr|T1RHM8|dnaK	0.578	0.282	0.411	FALSE	FALSE	FALSE	0	Chaperone protein DnaK
EF2	tr|W7UBY1|EF2	1.15	0.0868	0.206	FALSE	FALSE	FALSE	0	Elongation factor 2
FBA1	tr|W7TFC8|FBA1	-0.462	0.481	0.596	FALSE	FALSE	FALSE	0	Fructose-bisphosphate aldolase
FBP	tr|W7SYZ9|FBP	0.369	0.713	0.78	FALSE	FALSE	FALSE	0	Fructose--bisphosphatase
ftsH	tr|T1RHJ1|ftsH	0.55	0.346	0.47	FALSE	FALSE	FALSE	0	ATP-dependent zinc metalloprotease FtsH
FTSZ	tr|W7TYP0|FTSZ	1.13	0.216	0.35	FALSE	FALSE	TRUE	3	Cell division protein
G6PDH	tr|W7TZ08|G6PDH	1.11	0.0703	0.184	FALSE	FALSE	TRUE	4	Glucose-6-phosphate 1-dehydrogenase
GapC1	tr|W7T2R0|GapC1	1.38	0.259	0.396	FALSE	FALSE	FALSE	0	Glyceraldehyde-3-phosphate dehydrogenase
GCSL	tr|W7TT47|GCSL	-0.454	0.565	0.661	FALSE	FALSE	FALSE	0	Dihydrolipoamide dehydrogenase

GDH1	tr|W7TAN4|GDH1	1.12	0.232	0.369	FALSE	FALSE	TRUE	1	Glutamate dehydrogenase
gi|553175936|ref|XP_005852345.1|	gi|553175936|ref|XP_005852345.1|	1.57	0.034	0.123	FALSE	FALSE	FALSE	0	actin beta/gamma 1
gi|553180480|ref|XP_005853411.1|	gi|553180480|ref|XP_005853411.1|	0.828	0.505	0.614	FALSE	FALSE	TRUE	1	large subunit ribosomal protein L27Ae
gi|553182506|ref|XP_005853740.1|	gi|553182506|ref|XP_005853740.1|	2.59	0.014	0.0852	FALSE	FALSE	TRUE	4	rab18 -family small gtpase
gi|553183167|ref|XP_005853851.1|	gi|553183167|ref|XP_005853851.1|	1.38	0.12	0.239	FALSE	FALSE	TRUE	1	small nuclear ribonucleoprotein D1
gi|553184237|ref|XP_005854056.1|	gi|553184237|ref|XP_005854056.1|	0.638	0.271	0.406	FALSE	FALSE	FALSE	0	triosephosphate isomerase
gi|553185252|ref|XP_005854222.1|	gi|553185252|ref|XP_005854222.1|	1.59	0.0439	0.148	FALSE	FALSE	FALSE	0	glutamate decarboxylase
gi|578896496|gb|AHI17198.1|	gi|578896496|gb|AHI17198.1|	-0.405	0.514	0.621	FALSE	FALSE	FALSE	0	acetyl-CoA carboxylase
gi|578896498|gb|AHI17199.1|	gi|578896498|gb|AHI17199.1|	1.64	0.104	0.216	FALSE	FALSE	FALSE	0	acetyl-CoA carboxylase
gi|585099093|gb|EWM19969.1|	gi|585099093|gb|EWM19969.1|	1.08	0.108	0.221	FALSE	FALSE	FALSE	0	helicase at 25e
gi|585102433|gb|EWM22023.1|	gi|585102433|gb|EWM22023.1|	1.1	0.0692	0.183	FALSE	FALSE	FALSE	0	heat shock protein 101

gi|585111458|gb|EWM28970.1|	gi|585111458|gb|EWM28970.1|	2.05	0.0139	0.0852	FALSE	FALSE	TRUE	1	pyruvate dehydrogenase
gi|76780670|emb|CAH58676.1|	gi|76780670|emb|CAH58676.1|	-1.46	0.22	0.353	FALSE	FALSE	TRUE	1	proton-translocating inorganic pyrophosphatase
GOX	tr|W7UBQ6|GOX	1.3	0.0577	0.169	FALSE	FALSE	FALSE	0	Peroxisomal glycolate oxidase
GST	tr|W7TMQ1|GST	2.17	0.00623	0.0602	FALSE	FALSE	FALSE	0	Glutathione s-transferase
hemB	tr|W7TUE6|hemB	-0.237	0.738	0.8	FALSE	FALSE	FALSE	0	Delta-aminolevulinic acid dehydratase
Hsp	tr|W7TS47|Hsp	1.49	0.314	0.44	FALSE	FALSE	TRUE	2	Heat shock protein hsp90
HSP	tr|W7T7N9|HSP	-0.281	0.619	0.717	FALSE	FALSE	FALSE	0	Luminal binding protein
Hsp.1	tr|W7TNF9|Hsp	0.249	0.699	0.771	FALSE	FALSE	FALSE	0	Heat shock protein 90
HSP1	tr|W7TLX6|HSP1	-0.829	0.407	0.533	FALSE	FALSE	FALSE	0	Heat shock protein 90
ilvB	tr|T1RI34|ilvB	1.7	0.0924	0.206	FALSE	FALSE	TRUE	2	Acetolactate synthase large subunit
inorganic	tr|W7TN19|inorganic	-0.634	0.602	0.7	FALSE	FALSE	FALSE	0	H+-translocating pyrophosphatase family
LACS	tr|G9BBC7|LACS	1.63	0.0609	0.171	FALSE	FALSE	FALSE	0	Long-chain acyl-coenzyme A synthetase

LHC26	tr|W7UAI7|LHC26	0.683	0.279	0.409	FALSE	FALSE	FALSE	0	Light-harvesting protein
LHCP28	tr|W7TZB5|LHCP28	0.341	0.558	0.655	FALSE	FALSE	FALSE	0	Light-harvesting protein
LHCP5	tr|W7TCK1|LHCP5	0.45	0.493	0.606	FALSE	FALSE	FALSE	0	Chloroplast light harvesting protein isoform 4
Lhcv3	tr|W7TRI0|Lhcv3	-1.51	0.27	0.406	FALSE	FALSE	TRUE	1	Light-harvesting protein
Light-harvesting protein	tr|W7TZI5|Naga_100021g54	1.12	0.154	0.28	FALSE	FALSE	TRUE	3	Armadillo-like helical
MCAT	tr|S5VRZ9|MCAT	-0.229	0.698	0.771	FALSE	FALSE	FALSE	0	Malonyl-:acp transacylase
mcfO	tr|W7TZV3|mcfO	1.9	0.0176	0.0871	FALSE	FALSE	TRUE	4	Ef-hand domain-containing protein
nad11	tr|A0A023PJZ1|nad11	1.47	0.19	0.319	FALSE	FALSE	FALSE	0	NADH dehydrogenase subunit 11
nad7	tr|T1R883|nad7	0.0629	0.956	0.966	FALSE	FALSE	TRUE	2	NADH dehydrogenase subunit 7
Naga_100001g128	tr|W7TKJ5|Naga_100001g128	0.21	0.852	0.896	FALSE	FALSE	FALSE	0	Transketolase
Naga_100001g147	tr|W7TKL4|Naga_100001g147	-0.259	0.68	0.763	FALSE	FALSE	FALSE	0	Cytochrome c oxidase subunit vb
Naga_100001g189	tr|W7U259|Naga_100001g189	1.57	0.0153	0.087	FALSE	FALSE	FALSE	0	Nucleoside diphosphate kinase

Naga_100001g41	tr|W7U263|Naga_100001g41	1.31	0.0578	0.169	FALSE	FALSE	FALSE	0	Heat shock protein 70
Naga_100001g58	tr|W7U208|Naga_100001g58	3.22	0.0595	0.171	FALSE	FALSE	TRUE	1	Glyceraldehyde-3-phosphate dehydrogenase
Naga_100002g102	tr|W7TYW0|Naga_100002g102	1.53	0.0899	0.206	FALSE	FALSE	TRUE	2	Cystathionine gamma-lyase
Naga_100002g173	tr|W7TRD5|Naga_100002g173	2.31	0.178	0.303	FALSE	FALSE	TRUE	1	3-oxoacyl-[acyl-carrier-protein] synthase
Naga_100002g3	tr|W7TZ10|Naga_100002g3	0.288	0.864	0.897	FALSE	FALSE	TRUE	2	Mpv17/PMP22
Naga_100002g55	tr|W7TYV8|Naga_100002g55	0.192	0.845	0.893	FALSE	FALSE	TRUE	1	Vacuolar (H+)-ATPase G subunit
Naga_100003g103	tr|W7UBF9|Naga_100003g103	0.827	0.236	0.374	FALSE	FALSE	FALSE	0	Vacuolar h+-atpase a subunit
Naga_100003g133	tr|W7UBV8|Naga_100003g133	0.905	0.227	0.362	FALSE	FALSE	TRUE	1	Inosine-5-monophosphate dehydrogenase
Naga_100003g157	tr|W7TVY6|Naga_100003g157	1.49	0.0664	0.178	FALSE	FALSE	FALSE	0	Glucose-6-phosphate isomerase
Naga_100003g173	tr|W7U360|Naga_100003g173	0.901	0.251	0.388	FALSE	FALSE	FALSE	0	Atp-dependent metalloprotease

Naga_100003g177	tr|W7TVZ5|Naga_100003g177	2.47	0.00553	0.0549	FALSE	FALSE	FALSE	0	Heat shock protein 70
Naga_100003g67	tr|W7TSY3|Naga_100003g67	2.17	0.00798	0.0663	FALSE	FALSE	FALSE	0	Aconitate mitochondrial
Naga_100003g69	tr|W7UC18|Naga_100003g69	0.955	0.29	0.418	FALSE	FALSE	TRUE	3	Phosphoribosylformylglycinamidine synthase
Naga_100003g73	tr|W7UC45|Naga_100003g73	0.829	0.202	0.332	FALSE	FALSE	TRUE	4	Subunit of proteaseome activator complex
Naga_100003g83	tr|W7UC09|Naga_100003g83	-1.51	0.0614	0.171	FALSE	FALSE	TRUE	1	Porphobilinogen deaminase
Naga_100004g111	tr|W7U649|Naga_100004g111	-1.14	0.0816	0.204	FALSE	FALSE	FALSE	0	Thioredoxin f
Naga_100004g135	tr|W7TM71|Naga_100004g135	1.11	0.0585	0.169	FALSE	FALSE	FALSE	0	Ferredoxin
Naga_100004g79	tr|W7TQ45|Naga_100004g79	1.94	0.0932	0.206	FALSE	FALSE	TRUE	3	Glutaredoxin 2
Naga_100004g84	tr|W7TXD8|Naga_100004g84	1.31	0.136	0.257	FALSE	FALSE	TRUE	4	Gtp-binding protein sar1
Naga_100005g129	tr|W7TXW1|Naga_100005g129	2.51	0.0369	0.13	FALSE	FALSE	TRUE	4	Aminopeptidase-like 1

Naga_100005g139	tr|W7THB6|Naga_100005g139	1.87	0.0103	0.0764	FALSE	FALSE	FALSE	0	Vacuolar transporter chaperone 4
Naga_100005g16	tr|W7TPH9|Naga_100005g16	3.28	0.0844	0.206	FALSE	FALSE	TRUE	4	Peptidyl-prolyl cis-trans isomerase
Naga_100005g25	tr|W7TYA6|Naga_100005g25	0.555	0.744	0.804	FALSE	FALSE	TRUE	2	Photosystem ii 11 kd protein
Naga_100005g46	tr|W7TEP7|Naga_100005g46	0.761	0.507	0.615	FALSE	FALSE	FALSE	0	Histone h2b
Naga_100005g52	tr|W7TPN7|Naga_100005g52	1.51	0.0492	0.161	FALSE	FALSE	FALSE	0	Adp atp
Naga_100005g68	tr|W7TYC1|Naga_100005g68	-0.928	0.363	0.49	FALSE	FALSE	TRUE	1	Short-chain dehydrogenase reductase acting with nad or nadp as acceptor
Naga_100005g83	tr|W7TXT5|Naga_100005g83	1.79	0.0519	0.163	FALSE	FALSE	TRUE	1	ATP-dependent Clp protease proteolytic subunit
Naga_100006g108	tr|W7U9C3|Naga_100006g108	2.28	0.012	0.0812	FALSE	FALSE	TRUE	4	Dihydrolipoamide s-acetyltransferase
Naga_100006g25	tr|W7U9F9|Naga_100006g25	2.49	0.00752	0.0663	FALSE	FALSE	TRUE	4	Beta-lactamase/transpeptidase-like protein
Naga_100006g64	tr|W7U194|Naga_100006g64	1.51	0.0187	0.0905	FALSE	FALSE	FALSE	0	Delta-1-pyrroline-5-carboxylate synthetase

Naga_100006g89	tr|W7U9H6|Naga_100006g89	1.11	0.114	0.232	FALSE	FALSE	FALSE	0	Heat shock protein 90
Naga_100007g70	tr|W7U8K2|Naga_100007g70	2.21	0.00675	0.0637	FALSE	FALSE	FALSE	0	Clathrin heavy chain
Naga_100008g4	tr|W7TPQ0|Naga_100008g4	3.01	0.0952	0.206	FALSE	FALSE	TRUE	2	Atp synthase gamma
Naga_100009g12	tr|W7TN92|Naga_100009g12	3.09	0.0255	0.105	FALSE	FALSE	TRUE	1	6-phosphogluconate dehydrogenase, decarboxylating
Naga_100009g44	tr|W7U6Y7|Naga_100009g44	3.45	0.0091	0.0714	FALSE	FALSE	TRUE	4	Pyruvate decarboxylase
Naga_100009g63	tr|W7U6Y6|Naga_100009g63	0.015	0.98	0.987	FALSE	FALSE	FALSE	0	Eukaryotic translation initiation factor 5A
Naga_100009g67	tr|W7TR29|Naga_100009g67	1.06	0.0898	0.206	FALSE	FALSE	FALSE	0	Enolase
Naga_100009g84	tr|W7U6S0|Naga_100009g84	-0.243	0.774	0.83	FALSE	FALSE	FALSE	0	60s acidic ribosomal protein p0
Naga_100010g11	gi|553190821|ref|XP_005855269.1|	1.88	0.0256	0.105	FALSE	FALSE	TRUE	2	phosphoribosylaminoimidazolecarboxamide formyltransferase / IMP cyclohydrolase

Naga_100010g22	tr|W7TJF5|Naga_100010g22	-0.346	0.752	0.81	FALSE	FALSE	TRUE	2	Nucleoredoxin
Naga_100010g88	tr|W7U2S1|Naga_100010g88	1.71	0.191	0.319	FALSE	FALSE	TRUE	1	Ribosomal protein s16
Naga_100011g26	tr|W7TPX6|Naga_100011g26	1.36	0.144	0.268	FALSE	FALSE	TRUE	4	Phosphoglycerate mutase
Naga_100011g29	tr|W7TG29|Naga_100011g29	0.32	0.642	0.738	FALSE	FALSE	TRUE	2	Tic22-like protein
Naga_100011g39	tr|W7TG62|Naga_100011g39	1.93	0.104	0.216	FALSE	FALSE	TRUE	1	H-or na-translocating f-v-type and a-type atpase (F-atpase) superfamily
Naga_100011g54	tr|W7TPT5|Naga_100011g54	2.01	0.0172	0.087	FALSE	FALSE	TRUE	3	5-methyltetrahydropteroyltriglutamate--homocysteine s-methyltransferase
Naga_100012g48	tr|W7TMK0|Naga_100012g48	1.88	0.123	0.24	FALSE	FALSE	TRUE	3	Splicing factor u2af large subunit
Naga_100012g76	tr|W7TDH0|Naga_100012g76	2.68	0.026	0.105	FALSE	FALSE	TRUE	2	40s ribosomal protein
Naga_100013g72	tr|W7U3Y9|Naga_100013g72	0.581	0.282	0.411	FALSE	FALSE	FALSE	0	Nicotinamide nucleotide transhydrogenase

Naga_100013g92	tr|W7TWV3|Naga_100013g92	1.53	0.0631	0.173	FALSE	FALSE	TRUE	1	Cytochrome p450 hydroxylase
Naga_100014g51	tr|W7U0I2|Naga_100014g51	-1.13	0.253	0.389	FALSE	FALSE	FALSE	0	Ribosomal protein l12
Naga_100014g55	tr|W7TRK7|Naga_100014g55	1.28	0.0849	0.206	FALSE	FALSE	TRUE	2	Coatomer subunit alpha
Naga_100014g57	tr|W7TGL0|Naga_100014g57	1.93	0.0117	0.0812	FALSE	FALSE	TRUE	3	Uncharacterized protein
Naga_100014g7	tr|W7TGE1|Naga_100014g7	0.449	0.557	0.655	FALSE	FALSE	FALSE	0	Cupin 4 family protein
Naga_100015g11	tr|W7U4C6|Naga_100015g11	1.79	0.0235	0.102	FALSE	FALSE	FALSE	0	Citrate synthase
Naga_100016g24	tr|W7TG91|Naga_100016g24	0.876	0.126	0.244	FALSE	FALSE	FALSE	0	ATP synthase subunit beta
Naga_100016g59	tr|W7TNT0|Naga_100016g59	0.863	0.202	0.332	FALSE	FALSE	FALSE	0	ATPase, F0 complex, subunit B, mitochondrial
Naga_100017g18	tr|W7U1W1|Naga_100017g18	-0.289	0.709	0.777	FALSE	FALSE	FALSE	0	60s ribosomal protein l11
Naga_100017g20	tr|W7U1X5|Naga_100017g20	1.98	0.0295	0.112	FALSE	FALSE	FALSE	0	Transaldolase

Naga_100017g24	tr|W7U1V7|Naga_100017g24	2.44	0.0162	0.087	FALSE	FALSE	TRUE	1	Stress-inducible protein sti1
Naga_100019g53	tr|W7T4L1|Naga_100019g53	-0.922	0.383	0.508	FALSE	FALSE	FALSE	0	Histone H2A
Naga_100019g64	tr|W7T3J7|Naga_100019g64	1.13	0.0845	0.206	FALSE	FALSE	FALSE	0	Elongation factor 1-alpha
Naga_100020g11	tr|W7TLA3|Naga_100020g11	1.28	0.0862	0.206	FALSE	FALSE	FALSE	0	9STRA Prohibitin
Naga_100020g47	tr|W7TD69|Naga_100020g47	1.72	0.0197	0.0922	FALSE	FALSE	FALSE	0	Myo-inositol 2-dehydrogenase
Naga_100020g64	tr|W7TUE3|Naga_100020g64	2.51	0.0411	0.139	FALSE	FALSE	TRUE	1	Urease accessory protein ureg
Naga_100021g47	tr|W7TQL4|Naga_100021g47	1.54	0.167	0.296	FALSE	FALSE	TRUE	4	Cytochrome b-c1 complex subunit Rieske, mitochondrial
Naga_100021g53	tr|W7TQM4|Naga_100021g53	0.394	0.663	0.748	FALSE	FALSE	TRUE	3	Atp-dependent hsl protease atp-binding subunit hslu
Naga_100021g68	tr|W7TI88|Naga_100021g68	0.97	0.317	0.443	FALSE	FALSE	TRUE	3	Phosphoadenosine phosphosulfate reductase
Naga_100021g70	tr|W7TFK4|Naga_100021g70	2.42	0.0534	0.163	FALSE	FALSE	TRUE	1	Alpha tubulin 1

Naga_100021g72	tr|W7TYX5|Naga_100021g72	2.69	0.178	0.303	FALSE	FALSE	TRUE	2	Glycine cleavage system regulatory protein
Naga_100022g39	tr|W7TUA2|Naga_100022g39	2.07	0.0161	0.087	FALSE	FALSE	FALSE	0	Enolase
Naga_100022g53	tr|W7U1U2|Naga_100022g53	1.42	0.0262	0.105	FALSE	FALSE	FALSE	0	Isocitrate dehydrogenase [NADP]
Naga_100023g26	tr|W7TGK8|Naga_100023g26	1.97	0.0887	0.206	FALSE	FALSE	TRUE	1	Proteasome subunit beta type
Naga_100024g24	tr|W7U8G3|Naga_100024g24	1.79	0.0191	0.0911	FALSE	FALSE	FALSE	0	Atp-citrate synthase
Naga_100024g61	tr|W7TSC4|Naga_100024g61	2.37	0.0167	0.087	FALSE	FALSE	TRUE	3	Inositol-3-phosphate synthase
Naga_100025g47	tr|W7U0R4|Naga_100025g47	-0.974	0.486	0.601	FALSE	FALSE	FALSE	0	Ubiquitin ribosomal protein s27ae fusion protein
Naga_100026g22	tr|W7TW72|Naga_100026g22	2.33	0.0181	0.0886	FALSE	FALSE	TRUE	4	60s ribosomal protein l24
Naga_100026g40	tr|W7TWA9|Naga_100026g40	1.17	0.088	0.206	FALSE	FALSE	FALSE	0	14-3-3-like protein
Naga_100027g2	tr|W7TKH6|Naga_100027g2	1.99	0.00822	0.0663	FALSE	FALSE	TRUE	3	T-complex protein 1 subunit epsilon

Naga_100028g42	tr|K8YRK7|NGA_0361402	4.34	0.0112	0.0812	FALSE	FALSE	TRUE	2	Uncharacterized protein
Naga_100029g32	tr|W7TSX0|Naga_100029g32	1.16	0.056	0.168	FALSE	FALSE	FALSE	0	Plastidic atp adp transporter
Naga_100030g11	tr|W7TTN5|Naga_100030g11	1.74	0.0524	0.163	FALSE	FALSE	FALSE	0	Hybrid cluster protein
Naga_100030g5	tr|W7TTN9|Naga_100030g5	2.11	0.132	0.253	FALSE	FALSE	FALSE	0	Chlorophyll A-B binding protein
Naga_100031g28	tr|W7U0D4|Naga_100031g28	2.83	0.104	0.216	FALSE	FALSE	TRUE	1	Succinyl-ligase subunit mitochondrial
Naga_100031g39	tr|W7TS51|Naga_100031g39	-1.35	0.246	0.385	FALSE	FALSE	TRUE	1	40s ribosomal protein s3-3
Naga_100032g3	tr|W7TXZ6|Naga_100032g3	0.129	0.933	0.955	FALSE	FALSE	TRUE	3	40S ribosomal protein S3a
Naga_100032g30	tr|W7TY00|Naga_100032g30	0.538	0.679	0.763	FALSE	FALSE	TRUE	2	Coatomer subunit beta
Naga_100032g40	tr|W7THF5|Naga_100032g40	1.2	0.191	0.319	FALSE	FALSE	FALSE	0	Ribosomal protein L4 domain protein
Naga_100033g36	tr|W7TWV9|Naga_100033g36	0.188	0.9	0.924	FALSE	FALSE	TRUE	2	Peptidyl-prolyl cis-trans isomerase

Naga_100034g18	tr|W7TKT1|Naga_100034g18	0.702	0.446	0.577	FALSE	FALSE	FALSE	0	60s ribosomal protein l18a
Naga_100034g25	tr|W7U446|Naga_100034g25	2.62	0.0371	0.13	FALSE	FALSE	TRUE	1	Voltage-dependent anion-selective channel protein 2
Naga_100035g25	tr|W7UAU3|Naga_100035g25	2.36	0.023	0.102	FALSE	FALSE	TRUE	3	Homoserine kinase
Naga_100037g12	tr|W7TF53|Naga_100037g12	0.776	0.311	0.439	FALSE	FALSE	FALSE	0	3-oxoacyl-(Acyl-carrier-protein) reductase
Naga_100037g14	tr|W7THS5|Naga_100037g14	1.04	0.384	0.508	FALSE	FALSE	FALSE	0	Cyclophilin-like peptidyl-prolyl cis-trans isomerase
Naga_100038g29	tr|W7TUP5|Naga_100038g29	-0.879	0.53	0.634	FALSE	FALSE	TRUE	1	Atp synthase subunit delta
Naga_100038g3	tr|W7U315|Naga_100038g3	0.655	0.406	0.533	FALSE	FALSE	TRUE	3	Dead-box atp-dependent rna helicase
Naga_100038g9	tr|W7TMH8|Naga_100038g9	1.17	0.0635	0.173	FALSE	FALSE	FALSE	0	Udp-sulfoquinovose synthase
Naga_100040g37	tr|W7TUN8|Naga_100040g37	0.362	0.544	0.646	FALSE	FALSE	FALSE	0	Rieske (2fe-2s) region protein
Naga_100040g42	tr|W7TTU9|Naga_100040g42	1.65	0.0618	0.171	FALSE	FALSE	FALSE	0	Atp-dependent chaperone

Naga_100041g2	tr|W7TL25|Naga_100041g2	0.906	0.137	0.257	FALSE	FALSE	FALSE	0	Elongation factor 1
Naga_100041g31	tr|W7T232|Naga_100041g31	1.07	0.0918	0.206	FALSE	FALSE	FALSE	0	Adenosylhomocysteinase
Naga_100042g26	tr|W7U0U1|Naga_100042g26	1.23	0.0443	0.148	FALSE	FALSE	TRUE	2	Cad protein
Naga_100042g8	tr|W7TTB1|Naga_100042g8	2.71	0.013	0.0852	FALSE	FALSE	TRUE	2	3-ketoacyl-mitochondrial
Naga_100044g11	tr|W7U5I6|Naga_100044g11	1.75	0.0688	0.183	FALSE	FALSE	TRUE	1	40s ribosomal protein s15a
Naga_100044g19	tr|W7U5J3|Naga_100044g19	0.604	0.474	0.594	FALSE	FALSE	TRUE	4	Zz-type zinc finger-containing protein
Naga_100044g9	tr|W7TPS2|Naga_100044g9	2.42	0.0169	0.087	FALSE	FALSE	TRUE	3	Enhancer of rudimentary
Naga_100045g24	tr|W7TK84|Naga_100045g24	1.45	0.0527	0.163	FALSE	FALSE	FALSE	0	ATPase, AAA-type
Naga_100047g7	tr|W7TB36|Naga_100047g7	-0.762	0.5	0.611	FALSE	FALSE	TRUE	1	Succinyl-CoA ligase subunit beta
Naga_100050g29	tr|W7TR01|Naga_100050g29	0.173	0.84	0.891	FALSE	FALSE	FALSE	0	Histone H4

Naga_100050g33	tr|W7UA52|Naga_100050g33	0.121	0.882	0.91	FALSE	FALSE	FALSE	0	Histone H3
Naga_100050g34	tr|W7U9K9|Naga_100050g34	2.63	0.0262	0.105	FALSE	FALSE	FALSE	0	Histone H2A
Naga_100050g39	tr|W7U1E9|Naga_100050g39	1.77	0.0118	0.0812	FALSE	FALSE	FALSE	0	Uncharacterized protein
Naga_100051g22	tr|W7T495|Naga_100051g22	1.36	0.134	0.256	FALSE	FALSE	TRUE	4	Rab7 family gtpase
Naga_100051g29	tr|W7TM73|Naga_100051g29	1.2	0.0731	0.187	FALSE	FALSE	FALSE	0	Superoxide dismutase
Naga_100056g12	tr|W7TU57|Naga_100056g12	1.43	0.0609	0.171	FALSE	FALSE	FALSE	0	Phosphoenolpyruvate carboxykinase
Naga_100056g15	tr|W7TJ16|Naga_100056g15	0.424	0.433	0.564	FALSE	FALSE	FALSE	0	Light-harvesting protein
Naga_100059g26	tr|W7TVV4|Naga_100059g26	1.7	0.0886	0.206	FALSE	FALSE	FALSE	0	Guanine nucleotide binding protein
Naga_100061g16	tr|W7T2I2|Naga_100061g16	2.33	0.0173	0.087	FALSE	FALSE	TRUE	4	Carboxyl transferase
Naga_100061g24	tr|W7T2C3|Naga_100061g24	1.28	0.0871	0.206	FALSE	FALSE	TRUE	1	26s proteasome regulatory atpase rpt4

Naga_100061g9	tr|W7TKK2|Naga_100061g9	0.828	0.461	0.589	FALSE	FALSE	TRUE	1	40s ribosomal protein s23
Naga_100064g4	tr|W7TP37|Naga_100064g4	1.21	0.293	0.42	FALSE	FALSE	TRUE	1	Phosphoglycerate kinase
Naga_100065g10	tr|W7T5H8|Naga_100065g10	2.01	0.0141	0.0852	FALSE	FALSE	FALSE	0	Phosphoglucomutase
Naga_100065g23	tr|W7TE43|Naga_100065g23	0.823	0.313	0.44	FALSE	FALSE	FALSE	0	Leucyl aminopeptidase
Naga_100067g11	tr|W7TNV2|Naga_100067g11	2.93	0.0914	0.206	FALSE	FALSE	TRUE	1	Peptidyl-prolyl cis-trans isomerase
Naga_100067g28	tr|W7TFX4|Naga_100067g28	2.28	0.0198	0.0922	FALSE	FALSE	TRUE	3	Proteasome subunit alpha
Naga_100068g13	tr|W7TXC9|Naga_100068g13	0.887	0.299	0.426	FALSE	FALSE	TRUE	4	Heparanase-like protein 3
Naga_100069g1	tr|W7TKJ0|Naga_100069g1	1.16	0.261	0.398	FALSE	FALSE	TRUE	2	Phosphoglycerate mutase
Naga_100076g3	tr|W7TEW9|Naga_100076g3	-0.466	0.632	0.73	FALSE	FALSE	FALSE	0	Photosystem ii 12 kDa extrinsic protein
Naga_100078g15	tr|W7TS20|Naga_100078g15	0.818	0.435	0.565	FALSE	FALSE	FALSE	0	Elongation factor 3

Naga_100078g16	tr|W7TJN4|Naga_100078g16	0.621	0.694	0.771	FALSE	FALSE	TRUE	2	Elongation factor ef-3
Naga_100079g12	tr|W7TLT5|Naga_100079g12	1.61	0.0504	0.162	FALSE	FALSE	FALSE	0	Cysteine synthase
Naga_100081g17	tr|W7T2Q2|Naga_100081g17	1.3	0.0542	0.164	FALSE	FALSE	FALSE	0	Glyceraldehyde-3-phosphate dehydrogenase
Naga_100084g4	tr|W7TSQ4|Naga_100084g4	1.5	0.0218	0.0993	FALSE	FALSE	FALSE	0	S-adenosylmethionine synthase
Naga_100093g15	tr|W7TJM3|Naga_100093g15	0.668	0.239	0.376	FALSE	FALSE	FALSE	0	ClpB chaperone, Hsp100 family
Naga_100098g5	tr|W7TNK1|Naga_100098g5	2.8	0.00792	0.0663	FALSE	FALSE	TRUE	3	Cell division protein
Naga_100099g18	tr|W7U484|Naga_100099g18	0.289	0.651	0.745	FALSE	FALSE	FALSE	0	Ribosomal protein L15
Naga_100099g23	tr|W7TVG2|Naga_100099g23	-0.829	0.25	0.388	FALSE	FALSE	FALSE	0	Uncharacterized protein
Naga_100100g13	tr|W7TTE5|Naga_100100g13	1.73	0.0123	0.0818	FALSE	FALSE	FALSE	0	Gtp-binding nuclear protein ran
Naga_100102g18	tr|W7TRP0|Naga_100102g18	1.28	0.0912	0.206	FALSE	FALSE	FALSE	0	Eukaryotic initiation factor 4a

Naga_100102g2	tr|W7TRP6|Naga_100102g2	1.55	0.0238	0.102	FALSE	FALSE	FALSE	0	Heat shock protein 70
Naga_100103g9	tr|W7TLH5|Naga_100103g9	-0.582	0.682	0.763	FALSE	FALSE	TRUE	3	Ribosomal protein s20
Naga_100108g3	tr|W7TKA6|Naga_100108g3	0.0897	0.882	0.91	FALSE	FALSE	FALSE	0	40S ribosomal protein SA
Naga_100113g12	tr|W7TCB7|Naga_100113g12	1.18	0.466	0.592	FALSE	FALSE	TRUE	1	40s ribosomal protein s18
Naga_100113g20	tr|W7TUD0|Naga_100113g20	1.89	0.00809	0.0663	FALSE	FALSE	FALSE	0	Vacuolar h+ atpase b subunit
Naga_100114g2	tr|W7TR43|Naga_100114g2	0.355	0.559	0.655	FALSE	FALSE	FALSE	0	Photosystem II stability/assembly factor
Naga_100117g8	tr|W7TVJ9|Naga_100117g8	0.471	0.492	0.606	FALSE	FALSE	TRUE	2	2-oxoglutarate dehydrogenase e1 component
Naga_100118g22	tr|W7T749|Naga_100118g22	1.85	0.00936	0.0714	FALSE	FALSE	FALSE	0	Bacterioferritin comigratory protein
Naga_100119g3	tr|W7TQ47|Naga_100119g3	-0.428	0.699	0.771	FALSE	FALSE	TRUE	3	Fructose-bisphosphate aldolase
Naga_100120g1	tr|W7TPV3|Naga_100120g1	2.1	0.0138	0.0852	FALSE	FALSE	TRUE	1	Cell division protein

Naga_100122g10	tr|W7TFU2|Naga_100122g10	0.667	0.324	0.449	FALSE	FALSE	FALSE	0	Chaperonin
Naga_100124g18	tr|W7TU78|Naga_100124g18	2.14	0.0791	0.199	FALSE	FALSE	TRUE	1	Phosphoribosylpyrophosphate synthetase
Naga_100129g1	tr|W7UCU5|Naga_100129g1	2.05	0.0094	0.0714	FALSE	FALSE	FALSE	0	Poly binding protein 8
Naga_100145g17	tr|W7TJJ6|Naga_100145g17	1.98	0.0148	0.0869	FALSE	FALSE	TRUE	4	Proliferating cell nuclear antigen
Naga_100149g4	tr|W7U4E6|Naga_100149g4	2.56	0.0614	0.171	FALSE	FALSE	TRUE	3	Abc transporter g family member 7
Naga_100162g1	tr|W7TI24|Naga_100162g1	1.13	0.247	0.385	FALSE	FALSE	TRUE	1	Acidocalcisomal pyrophosphatase
Naga_100164g14	tr|W7TIS3|Naga_100164g14	1.88	0.152	0.277	FALSE	FALSE	TRUE	1	Peroxiredoxin 1
Naga_100171g15	tr|W7TC86|Naga_100171g15	-0.0216	0.983	0.987	FALSE	FALSE	TRUE	1	Protein transport protein sec61 subunit alpha
Naga_100171g8	tr|W7TWB2|Naga_100171g8	1.59	0.0568	0.169	FALSE	FALSE	FALSE	0	Chaperonin Cpn60/TCP-1
Naga_100175g2	tr|W7TED5|Naga_100175g2	1.25	0.206	0.336	FALSE	FALSE	TRUE	1	RNA binding protein

Naga_100186g2	tr|W7TXA7|Naga_100186g2	-1.45	0.321	0.447	FALSE	FALSE	TRUE	3	Nucleolar protein expressed
Naga_100186g6	tr|W7TXA2|Naga_100186g6	0.935	0.29	0.418	FALSE	FALSE	FALSE	0	Proteasome subunit beta type
Naga_100187g5	tr|W7TW63|Naga_100187g5	1.15	0.117	0.235	FALSE	FALSE	FALSE	0	Trimeric LpxA
Naga_100187g6	tr|W7TKZ3|Naga_100187g6	2.05	0.0146	0.0869	FALSE	FALSE	TRUE	3	The actin binding protein cofilin-like protein
Naga_100187g8	tr|W7TW67|Naga_100187g8	1.92	0.0318	0.118	FALSE	FALSE	FALSE	0	Transitional endoplasmic reticulum atpase
Naga_100189g5	tr|W7TSS0|Naga_100189g5	0.733	0.341	0.47	FALSE	FALSE	TRUE	2	Aspartate-semialdehyde dehydrogenase
Naga_100194g2	ANT70526.1	0.321	0.642	0.738	FALSE	FALSE	FALSE	0	zeaxanthin epoxidase 1
Naga_100207g6	tr|W7THD6|Naga_100207g6	-0.198	0.864	0.897	FALSE	FALSE	TRUE	2	Geranylgeranyl reductase
Naga_100208g4	tr|W7TPK9|Naga_100208g4	0.452	0.707	0.777	FALSE	FALSE	TRUE	2	Acetohydroxy acid isomeroreductase, catalytic
Naga_100210g4	tr|W7UBW7|Naga_100210g4	0.578	0.447	0.577	FALSE	FALSE	TRUE	3	Nadh dehydrogenase subunit 11
Naga_100228g7	tr|W7T0L9|Naga_100228g7	1.37	0.09	0.206	FALSE	FALSE	FALSE	0	RNA helicase,ATP-

									dependent, DEAD-box type
Naga_100244g5	tr|W7TNV1|Naga_100244g5	1.5	0.0331	0.121	FALSE	FALSE	FALSE	0	Uncharacterized protein
Naga_100257g1	tr|W7U445|Naga_100257g1	0.83	0.145	0.269	FALSE	FALSE	FALSE	0	Glyceraldehyde-3-phosphate dehydrogenase
Naga_100273g6	tr|W7TJZ9|Naga_100273g6	0.882	0.168	0.296	FALSE	FALSE	FALSE	0	Extrinsic protein in photosystem ii
Naga_100273g9	tr|W7TTD1|Naga_100273g9	-0.13	0.859	0.896	FALSE	FALSE	FALSE	0	Nucleoside diphosphate kinase
Naga_100298g8	tr|W7T5F4|Naga_100298g8	1.13	0.124	0.242	FALSE	FALSE	TRUE	2	T-complex protein 1 subunit alpha
Naga_100300g2	tr|W7T2V5|Naga_100300g2	1.68	0.0536	0.163	FALSE	FALSE	TRUE	1	Proteasome subunit alpha
Naga_100308g2	tr|W7TS11|Naga_100308g2	-0.637	0.274	0.406	FALSE	FALSE	FALSE	0	Ribosomal protein l5
Naga_100331g6	tr|W7T3A1|Naga_100331g6	-0.355	0.721	0.786	FALSE	FALSE	TRUE	2	Caltractin
Naga_100348g1	tr|W7TXQ4|Naga_100348g1	0.874	0.237	0.374	FALSE	FALSE	FALSE	0	Ribosomal protein S7, conserved site
Naga_100351g2	tr|W7UAH6|Naga_100351g2	0.361	0.696	0.771	FALSE	FALSE	TRUE	2	Glyoxalase domain-containing protein 4

Naga_100385g2	tr|W7TK08|Naga_100385g2	-0.417	0.468	0.592	FALSE	FALSE	FALSE	0	Acyl carrier protein
Naga_100385g5	tr|W7T0Z4|Naga_101026g1	1.37	0.136	0.257	FALSE	FALSE	TRUE	3	Hydrolase
Naga_100410g3	tr|W7TJY6|Naga_100410g3	0.618	0.276	0.406	FALSE	FALSE	FALSE	0	Phosphoglycerate kinase
Naga_100418g3	tr|W7TP04|Naga_100418g3	0.657	0.31	0.439	FALSE	FALSE	TRUE	4	Sumo-activating enzyme subunit 2
Naga_100419g4	tr|W7U2N9|Naga_100419g4	1.36	0.113	0.23	FALSE	FALSE	TRUE	2	Ornithine aminotransferase
Naga_100466g3	tr|W7TLR9|Naga_100466g3	2.34	0.0296	0.112	FALSE	FALSE	TRUE	2	Trifunctional enzyme subunit mitochondrial
Naga_100475g1	tr|W7TJS6|Naga_100475g1	2.05	0.123	0.24	FALSE	FALSE	TRUE	1	Uncharacterized protein
Naga_100529g1	tr|W7TQA6|Naga_100529g1	0.93	0.17	0.297	FALSE	FALSE	FALSE	0	Fructokinase
Naga_100594g3	tr|W7TSZ8|Naga_100594g3	0.218	0.893	0.919	FALSE	FALSE	TRUE	1	Branched-chain alpha-keto acid dehydrogenase subunit e2
Naga_100638g4	tr|W7T973|Naga_100638g4	0.665	0.525	0.633	FALSE	FALSE	TRUE	1	40s ribosomal protein s13

Naga_100638g 4.1	tr|W7T8M4|Naga_100430g3	1.02	0.1	0.213	FALSE	FALSE	FALSE	0	Translationally controlled tumor protein
Naga_100641g 3	tr|K8YQ29|NGA_0635410	0.685	0.27	0.406	FALSE	FALSE	FALSE	0	H+-transporting ATPase
Naga_100641g 3 .1	tr|W7T8I0|Naga_100641g3	0.241	0.66	0.748	FALSE	FALSE	FALSE	0	Light-harvesting protein
Naga_100665g 2	gi|553194809|ref|XP_005856032.1|	0.362	0.655	0.746	FALSE	FALSE	TRUE	2	coproporphyrinogen III oxidase
Naga_100710g 1	tr|W7TQV0|Naga_100710g1	2.3	0.274	0.406	FALSE	FALSE	TRUE	1	Elongation factor tu
Naga_100855g 2	tr|W7TN59|Naga_100855g2	1.94	0.0165	0.087	FALSE	FALSE	FALSE	0	Malate dehydrogenase
Naga_100881g 1	tr|W7TMV0|Naga_100881g1	1.4	0.049	0.161	FALSE	FALSE	FALSE	0	Heat shock protein 101
Naga_100928g 1	tr|W7TQR4|Naga_100928g1	2.87	0.00753	0.0663	FALSE	FALSE	FALSE	0	Atp-dependent rna helicase uap56
Naga_100967g 1	tr|W7TSC3|Naga_100967g1	0.534	0.344	0.47	FALSE	FALSE	FALSE	0	Beta-ig-h3 fasciclin
Naga_101137g 1	tr|W7UCV3|Naga_101137g1	-0.0808	0.943	0.96	FALSE	FALSE	TRUE	1	Polyadenylate-binding protein

Naga_101273g 1	tr|W7THF6|Naga_101273g1	1.29	0.271	0.406	FALSE	FALSE	TRUE	1	3-isopropylmalate dehydrogenase
Naga_102104g 1	tr|W7T7S0|Naga_100245g2	1.06	0.0847	0.206	FALSE	FALSE	FALSE	0	Nucleotide-binding, alpha-beta plait
Naga_102104g 1 .1	tr|W7T7X5|Naga_102104g1	1.24	0.163	0.292	FALSE	FALSE	TRUE	4	Soluble pyridine nucleotide transhydrogenase
NAG-PR	tr|W7TID0|NAG-PR	0.337	0.727	0.79	FALSE	FALSE	TRUE	1	N-acetyl-gamma-glutamyl-phosphate reductase
nd9	tr|K9ZXV6|nd9	0.957	0.116	0.235	FALSE	FALSE	FALSE	0	NADH dehydrogenase subunit 9
NGA_0045802	tr|K8Z8Q4|NGA_0045802	1.92	0.0118	0.0812	FALSE	FALSE	FALSE	0	Chaperonin 10
NGA_0094200	tr|K8YQS9|NGA_0094200	-1.3	0.0496	0.161	FALSE	FALSE	FALSE	0	4-nitrophenyl phosphatase
NGA_0096400	tr|K8YQP9|NGA_0096400	-2.25	0.00494	0.0503	FALSE	FALSE	TRUE	2	Nad-dependent epimerase dehydratase
NGA_0130510	tr|K8YT62|NGA_0130510	1.83	0.073	0.187	FALSE	FALSE	TRUE	2	Carrier protein
NGA_0189801	tr|K8YTS8|NGA_0189801	0.16	0.772	0.83	FALSE	FALSE	FALSE	0	Light-harvesting protein

NGA_0190001	tr|K8YWQ7|NGA_0190001	-0.735	0.531	0.634	FALSE	FALSE	TRUE	1	Uncharacterized protein
NGA_0271420	tr|K8YVV3|NGA_0271420	2.13	0.0207	0.0951	FALSE	FALSE	TRUE	2	Uncharacterized protein
NGA_0366400	tr|K8YRV9|NGA_0366400	-0.739	0.207	0.336	FALSE	FALSE	FALSE	0	Light-harvesting protein
NGA_0391400	gi|585100011|gb|EWM20418.1|	0.536	0.389	0.512	FALSE	FALSE	FALSE	0	oxidoreductase
NGA_0448400	tr|K8Z7F5|NGA_0448400	2.49	0.178	0.303	FALSE	FALSE	TRUE	3	Soul heme-binding protein
NGA_0504200	tr|K8YVY4|NGA_0504200	1.5	0.0275	0.108	FALSE	FALSE	FALSE	0	Glycine-rich rna-binding protein 4
NGA_0599100	tr|K8YQB4|NGA_0599100	1.15	0.175	0.303	FALSE	FALSE	TRUE	3	H+-transporting ATPase
NGA_0612301	tr|K8YZ78|NGA_0612301	1.98	0.0165	0.087	FALSE	FALSE	FALSE	0	Mitochondrial protein translocase family
NGA_0699400	tr|K8YWB4|NGA_0699400	1.17	0.137	0.257	FALSE	FALSE	FALSE	0	Light harvesting complex protein
NGATSA_3001900	tr|I2CQN8|NGATSA_3001900	1.97	0.0787	0.199	FALSE	FALSE	TRUE	3	Eukaryotic translation initiation factor isoform 1

NGATSA_3003 100	tr|I2CQP8|NGATSA_3003100	2.21	0.0518	0.163	FALSE	FALSE	TRUE	1	Glycine dehydrogenase
NGATSA_3003 200 .1	tr|I2CQQ0|NGATSA_3003200	2.46	0.0944	0.206	FALSE	FALSE	FALSE	0	Uncharacterized protein
NGATSA_3004 500	tr|I2CQR0|NGATSA_3004500	0.00948	0.987	0.987	FALSE	FALSE	FALSE	0	Glutamate-1-semialdehyde aminotransferase/glutamate-1-semialdehyde 21-aminomutase
NGATSA_3016 900	tr|I2CR44|NGATSA_3016900	-0.813	0.291	0.418	FALSE	FALSE	FALSE	0	Uncharacterized protein
NGATSA_3021 000	tr|I2CP43|NGATSA_3021000	1.37	0.155	0.28	FALSE	FALSE	TRUE	4	T-complex protein 1 subunit beta
NGATSA_3026 900	gi|585105860|gb|EWM24426.1|	1.84	0.0172	0.087	FALSE	FALSE	FALSE	0	asf sf2-like pre-mrna splicing factor srp31
NGATSA_3041 300	tr|I2CPH3|NGATSA_3041300	1.42	0.0408	0.139	FALSE	FALSE	FALSE	0	Large subunit ribosomal protein L8e
OsNAAT1	tr|W7TAZ1|OsNAAT1	1.68	0.0283	0.109	FALSE	FALSE	TRUE	3	Alanine-2-oxoglutarate aminotransferase 2
PAO2	tr|W7TRP5|PAO2	2.7	0.0229	0.102	FALSE	FALSE	TRUE	3	Pheophorbide a oxygenase
PDGH	tr|W7U408|PDGH	1.57	0.101	0.213	FALSE	FALSE	TRUE	1	Phosphoserine aminotransferase

PDH	tr|W7TYT3|PDH	-1.37	0.108	0.222	FALSE	FALSE	FALSE	0	Transketolase
Pdi3	tr|W7TES6|Pdi3	2.71	0.0368	0.13	FALSE	FALSE	TRUE	2	Protein disulfide isomerase
PDK1	tr|W7TC29|PDK1	1.07	0.272	0.406	FALSE	FALSE	TRUE	4	Signal transduction histidine kinase, core
PEFG	tr|W7THY0|PEFG	-1.81	0.146	0.269	FALSE	FALSE	TRUE	1	Elongation factor G, mitochondrial
petA	tr|T1RJB3|petA	0.419	0.478	0.596	FALSE	FALSE	FALSE	0	Apocytochrome f
petB	tr|K9ZX12|petB	0.971	0.147	0.27	FALSE	FALSE	FALSE	0	Cytochrome b6
PETC	tr|W7TXJ0|PETC	1.25	0.473	0.594	FALSE	FALSE	TRUE	2	Cytochrome b6-f complex iron-sulfur subunit
petF	tr|K9ZV85|petF	-0.246	0.798	0.853	FALSE	FALSE	FALSE	0	Ferredoxin
PETH	tr|K8YXT7|PETH	0.0372	0.955	0.966	FALSE	FALSE	FALSE	0	Ferredoxin--NADP+ reductase
petJ	tr|T1RJX4|petJ	0.104	0.847	0.893	FALSE	FALSE	FALSE	0	Cytochrome c6
PPX	tr|W7TSK2|PPX	0.603	0.275	0.406	FALSE	FALSE	FALSE	0	Protoporphyrinogen oxidase
PRK	tr|W7TMN7|PRK	0.465	0.533	0.634	FALSE	FALSE	FALSE	0	Phosphoribulokinase
psaB	tr|T1RJ53|psaB	0.877	0.159	0.286	FALSE	FALSE	FALSE	0	Photosystem I P700 chlorophyll a apoprotein A2
psaC	tr|T1RJY1|psaC	-1.03	0.165	0.294	FALSE	FALSE	FALSE	0	Photosystem I iron-sulfur center

psaD	tr|T1RJN5|psaD	0.683	0.37	0.493	FALSE	FALSE	FALSE	0	Photosystem I reaction center subunit II
psaE	tr|T1RJ36|psaE	1.58	0.033	0.121	FALSE	FALSE	FALSE	0	Photosystem I reaction center subunit IV
psaF	tr|T1RIP4|psaF	0.522	0.376	0.5	FALSE	FALSE	FALSE	0	Photosystem I subunit III
psaL	tr|T1RJZ1|psaL	0.76	0.185	0.313	FALSE	FALSE	FALSE	0	Photosystem I reaction center subunit XI
psbA	tr|T1RJV8|psbA	0.432	0.479	0.596	FALSE	FALSE	FALSE	0	Photosystem II protein D1
psbB	tr|T1RJL7|psbB	1.09	0.101	0.213	FALSE	FALSE	FALSE	0	Photosystem II CP47 reaction center protein
psbC	tr|T1RJG1|psbC	0.131	0.829	0.881	FALSE	FALSE	FALSE	0	Photosystem II CP43 reaction center protein
psbD	tr|T1RIM2|psbD	-0.273	0.656	0.746	FALSE	FALSE	FALSE	0	Photosystem II D2 protein
psbE	tr|T1RHR2|psbE	-1.37	0.12	0.239	FALSE	FALSE	FALSE	0	Cytochrome b559 subunit alpha
psbO	tr|W7TVN8|psbO	-0.293	0.583	0.68	FALSE	FALSE	FALSE	0	Oxygen-evolving enhancer protein
psbV	tr|T1RJ27|psbV	0.0983	0.859	0.896	FALSE	FALSE	FALSE	0	Cytochrome c-550
PYK	tr|W7TRP2|PYK	1.42	0.0586	0.169	FALSE	FALSE	FALSE	0	Pyruvate kinase
Rab1B	tr|W7TST3|Rab1B	1.87	0.0947	0.206	FALSE	FALSE	TRUE	1	Rab family gtpase

rbcL	tr|T1RJ90|rbcL	-0.626	0.294	0.42	FALSE	FALSE	FALSE	0	Ribulose bisphosphate carboxylase large chain
rbcS	tr|A0A023PMA5|rbcS	-0.114	0.854	0.896	FALSE	FALSE	FALSE	0	Ribulose bisphosphate carboxlyase small chain
RPE	tr|W7TD18|RPE	1.64	0.0251	0.105	FALSE	FALSE	FALSE	0	Ribulose-phosphate 3-epimerase
RPIA	tr|K8Z9G7|RPIA	0.619	0.334	0.462	FALSE	FALSE	FALSE	0	Ribose 5-phosphate isomerase A
rpl12	tr|T1RJ33|rpl12	-1.86	0.031	0.116	FALSE	FALSE	TRUE	1	50S ribosomal protein L12, chloroplastic
RP-L6E	tr|K8YU30|HADH	1.88	0.0977	0.209	FALSE	FALSE	TRUE	4	3-hydroxyacyl-CoA dehydrogenase
RP-L6E.1	tr|K8YU91|ACSS	-0.548	0.551	0.652	FALSE	FALSE	TRUE	1	Acetyl-coenzyme A synthetase
rps13	tr|T1RJU6|rps13	-1.52	0.0922	0.206	FALSE	FALSE	TRUE	3	30S ribosomal protein S13, chloroplastic
RP-S2E	tr|K8Z375|RP-S2E	1.04	0.0788	0.199	FALSE	FALSE	FALSE	0	Small subunit ribosomal protein S2e
RP-S9E	tr|K8Z8Z7|RP-S9E	1.4	0.173	0.301	FALSE	FALSE	TRUE	1	Small subunit ribosomal protein S9e

SBP	tr|W7TFY2|SBP	0.106	0.945	0.96	FALSE	FALSE	TRUE	1	Chloroplast sedoheptulose--bisphosphatase
SEC11	tr|K8ZB45|SEC11	1.81	0.0535	0.163	FALSE	FALSE	TRUE	3	Signal peptidase I
SHMT2.1	tr|W7U077|SHMT2	1.21	0.0885	0.206	FALSE	FALSE	FALSE	0	Serine hydroxymethyltransferase
Snu114	tr|W7TM02|Snu114	0.796	0.346	0.47	FALSE	FALSE	TRUE	2	Small nuclear ribonucleoprotein component
SNU13	tr|K8YVC8|SNU13	1.3	0.122	0.24	FALSE	FALSE	TRUE	3	U4/U6 small nuclear ribonucleoprotein SNU13
tufA	tr|T1RJ79|tufA	0.479	0.466	0.592	FALSE	FALSE	FALSE	0	Elongation factor Tu, chloroplastic
UGD	AQR53217.1	1.78	0.0136	0.0852	FALSE	FALSE	FALSE	0	UDP-glucose 6-dehydrogenase
VDAC	tr|W7U0F0|VDAC	1.2	0.0654	0.177	FALSE	FALSE	FALSE	0	Voltage-dependent anion-selective channel
XBP3	tr|W7TP29|XBP3	0.0151	0.987	0.987	FALSE	FALSE	TRUE	2	Fructose--bisphosphatase

Supplementary Table 3. Label-free quantification (LFQ) analyst results for M1 mutant *N. oculata* Day 2 vs Day 12 samples.

Gene Name	*Protein IDs*	*M1Day12_vs_M1Day2_log2 fold change*	*M1Day12_vs_M1Day2_p.val*	*M1Day12_vs_M1Day2_p.adj*	*significant*	*M1Day12_vs_M1Day2_significant*	*imputed*	*num_NAs*	*Protein names*
AFB75402.1	AFB75402.1	5.15	0.000467	0.0438	TRUE	TRUE	TRUE	1	lipid droplet surface protein
DKC1, Naga_100028g51	tr|K8Z676|DKC1	4.31	0.00086	0.0438	TRUE	TRUE	TRUE	3	H/ACA ribonucleoprotein complex subunit 4
gi|585102433|gb|EWM22023.1|	gi|585102433|gb|EWM22023.1|	5.2	0.000159	0.0311	TRUE	TRUE	TRUE	2	heat shock protein 101
HADH, Naga_100113g7	tr|K8YU30|HADH	4.62	0.000406	0.0438	TRUE	TRUE	TRUE	3	3-hydroxyacyl-CoA dehydrogenase
Naga_100013g52	tr|W7UCJ8|Naga_100013g52	4.87	0.000992	0.0438	TRUE	TRUE	TRUE	2	Fatty acid desaturase type 2
Naga_100033g36	tr|W7TWV9|Naga_100033g36	4.95	0.000677	0.0438	TRUE	TRUE	TRUE	2	Peptidyl-prolyl cis-trans isomerase
Naga_100034g25	tr|W7U446|Naga_100034g25	4.23	0.000664	0.0438	TRUE	TRUE	TRUE	3	Voltage-dependent anion-selective channel protein 2
Naga_100040g37	tr|W7TUN8|Naga_100040g37	5.7	3.78E-05	0.0148	TRUE	TRUE	FALSE	0	Rieske (2fe-2s) region protein

PRX2	tr|W7TZN7|PRX2	4.14	0.00101	0.0438	TRUE	TRUE	TRUE	3	Thioredoxin-dependent peroxide reductase
AAB94637.1	AAB94637.1	1.48	0.18	0.324	FALSE	FALSE	FALSE	0	violaxanthin/chlorophyll a binding protein precursor
AAT	tr|W7U3D6|AAT	1.53	0.201	0.344	FALSE	FALSE	TRUE	1	Aspartate aminotransferase
ACSS, Naga_100028g54, Naga_100405g2, Naga_100405g2, NGATSA_3002000	tr|K8YU91|ACSS	1.05	0.343	0.473	FALSE	FALSE	TRUE	1	Acetyl-coenzyme A synthetase
AFJ69311.1	AFJ69311.1	2.51	0.0863	0.242	FALSE	FALSE	TRUE	1	2-O-methyltransferase fibrillarin
AIU44072.1	AIU44072.1	3.3	0.00796	0.103	FALSE	FALSE	TRUE	3	methylmalonyl CoA mutase
Arf1	tr|W7TL63|Arf1	0.392	0.702	0.758	FALSE	FALSE	FALSE	0	Adp-ribosylation factor
ASS	tr|W7U626|ASS	2.92	0.0556	0.213	FALSE	FALSE	TRUE	1	Argininosuccinate synthase
atp1	tr|T1R8F9|atp1	1.36	0.203	0.347	FALSE	FALSE	FALSE	0	ATP synthase subunit alpha
ATP1	tr|W7TTR4|ATP1	1.05	0.326	0.458	FALSE	FALSE	TRUE	4	p-type atpase

atp8	tr|T1R7J2|atp8	2.6	0.0664	0.222	FALSE	FALSE	TRUE	1	ATP synthase F0 subunit 8
atpA	tr|T1RIM9|atpA	0.792	0.433	0.549	FALSE	FALSE	FALSE	0	ATP synthase subunit alpha, chloroplastic
atpB	tr|T1RHE4|atpB	0.972	0.344	0.474	FALSE	FALSE	FALSE	0	ATP synthase subunit beta, chloroplastic
atpD	tr|T1RJB4|atpD	1.23	0.227	0.369	FALSE	FALSE	FALSE	0	ATP synthase CF1 delta chain
atpE	tr|T1RJM9|atpE	1.29	0.215	0.36	FALSE	FALSE	FALSE	0	ATP synthase epsilon chain, chloroplastic
ATPEF1O, Naga_100066g9	tr|K8YVX1|ATPEF1O	2.11	0.0718	0.224	FALSE	FALSE	FALSE	0	F-type H+-transporting ATPase oligomycin sensitivity conferral protein
atpF	tr|T1RIU1|atpF	0.648	0.522	0.63	FALSE	FALSE	FALSE	0	CF0 subunit I of ATP synthase
atpG	tr|T1RIS0|atpG	1.32	0.264	0.406	FALSE	FALSE	FALSE	0	F0F1 ATP synthase subunit B
ATS	tr|W7TEH2|ATS	0.774	0.5	0.613	FALSE	FALSE	FALSE	0	Sulfate adenylyltransferase
CAT	tr|W7T2K9|CAT	3.14	0.0148	0.122	FALSE	FALSE	TRUE	2	Catalase
cbbX	tr|W7TPW8|cbbX	2.53	0.0703	0.224	FALSE	FALSE	FALSE	0	Rubisco expression protein

cbbX.1	tr|T1RJ59|cbbX	1.25	0.243	0.384	FALSE	FALSE	FALSE	0	Putative rubisco expression protein
chlI	tr|T1RJ86|chlI	0.851	0.573	0.68	FALSE	FALSE	TRUE	1	Magnesium chelatase subunit
clpC-I	tr|T1RJA6|clpC-I	1.26	0.237	0.377	FALSE	FALSE	FALSE	0	ATP-dependent Clp protease ATPase subunit
clpC-II	tr|T1RJR9|clpC-II	2.01	0.0787	0.234	FALSE	FALSE	TRUE	2	ATP-dependent Clp protease
clpC-II, clpC	tr|T1RJA1|clpC-II	2.16	0.106	0.274	FALSE	FALSE	TRUE	1	ATP-dependent Clp protease
clpP	tr|W7TMY3|clpP	1.18	0.253	0.395	FALSE	FALSE	FALSE	0	ATP-dependent Clp protease proteolytic subunit
cox2	tr|T1R854|cox2	2.36	0.0744	0.228	FALSE	FALSE	TRUE	1	Cytochrome c oxidase subunit 2
CPS	tr|W7U5E4|CPS	3.06	0.0161	0.127	FALSE	FALSE	FALSE	0	Carbamoyl-phosphate synthase
CYN	tr|W7TQJ8|CYN	2.88	0.00931	0.107	FALSE	FALSE	FALSE	0	Peptidyl-prolyl cis-trans isomerase
dbj	gi|224809175|dbj|BAH28795.1|	1.01	0.319	0.453	FALSE	FALSE	FALSE	0	glyceraldehyde-3-phosphate dehydrogenase
dnaK	tr|T1RHM8|dnaK	0.811	0.424	0.543	FALSE	FALSE	FALSE	0	Chaperone protein DnaK
EF2	tr|W7UBY1|EF2	0.654	0.563	0.67	FALSE	FALSE	FALSE	0	Elongation factor 2

FBA1	tr|W7TFC8|FBA1	1.13	0.3	0.446	FALSE	FALSE	FALSE	0	Fructose-bisphosphate aldolase
FBP	tr|W7SYZ9|FBP	0.992	0.339	0.471	FALSE	FALSE	FALSE	0	Fructose--bisphosphatase
FBP.1	tr|W7TEP4|FBP	2.41	0.0577	0.213	FALSE	FALSE	TRUE	1	Fructose--bisphosphatase
ftsH	tr|T1RHJ1|ftsH	1.27	0.253	0.395	FALSE	FALSE	FALSE	0	ATP-dependent zinc metalloprotease FtsH
GapC1	tr|W7T2R0|GapC1	1.54	0.228	0.369	FALSE	FALSE	FALSE	0	Glyceraldehyde-3-phosphate dehydrogenase
GCSL	tr|W7U9J4|GCSL	4.01	0.00363	0.0933	FALSE	FALSE	TRUE	2	Dihydrolipoyl dehydrogenase
GDH1	tr|W7TAN4|GDH1	3.65	0.00455	0.0939	FALSE	FALSE	TRUE	3	Glutamate dehydrogenase
gi|553175936|ref|XP_005852345.1|	gi|553175936|ref|XP_005852345.1|	1.07	0.311	0.453	FALSE	FALSE	FALSE	0	actin beta/gamma 1
gi|578896498|gb|AHI17199.1|	gi|578896498|gb|AHI17199.1|	2.29	0.0413	0.199	FALSE	FALSE	FALSE	0	acetyl-CoA carboxylase
gi|585099093|gb|EWM19969.1|	gi|585099093|gb|EWM19969.1|	2.04	0.153	0.305	FALSE	FALSE	TRUE	1	helicase at 25e
gi|585111458|gb|EWM28970.1|	gi|585111458|gb|EWM28970.1|	2.9	0.0104	0.112	FALSE	FALSE	TRUE	1	pyruvate dehydrogenase

GLMS, Naga_100246 g4	tr|K8YQF5|GLMS	2.93	0.0149	0.122	FALSE	FALSE	TRUE	2	Glucosamine--fructose-6-phosphate aminotransferase (Isomerizing)
GOX	tr|W7UBQ6|GOX	2.59	0.0286	0.163	FALSE	FALSE	FALSE	0	Peroxisomal glycolate oxidase
groEL	tr|A0A023PLN1|groEL	0.104	0.926	0.953	FALSE	FALSE	TRUE	3	chaperonin, chloroplastic
GSR	tr|W7U997|GSR	2.53	0.046	0.202	FALSE	FALSE	TRUE	4	Glutathione reductase
GST	tr|W7TMQ1|GST	2.03	0.0931	0.249	FALSE	FALSE	TRUE	1	Glutathione s-transferase
HDAC1_2, Naga_100270 g5	tr|K8YR89|HDAC1_2	0.397	0.693	0.756	FALSE	FALSE	TRUE	4	Histone deacetylase
hemB	tr|W7TUE6|hemB	1.31	0.225	0.369	FALSE	FALSE	FALSE	0	Delta-aminolevulinic acid dehydratase
HSDH	tr|W7TUB9|HSDH	2.13	0.0525	0.209	FALSE	FALSE	FALSE	0	Homoserine dehydrogenase
Hsp	tr|W7TS47|Hsp	2.06	0.128	0.282	FALSE	FALSE	TRUE	1	Heat shock protein hsp90
HSP	tr|W7T7N9|HSP	0.428	0.699	0.757	FALSE	FALSE	FALSE	0	Luminal binding protein
Hsp.1	tr|W7TNF9|Hsp	0.496	0.673	0.749	FALSE	FALSE	FALSE	0	Heat shock protein 90
HSP1	tr|W7TLX6|HSP1	1.2	0.322	0.455	FALSE	FALSE	TRUE	1	Heat shock protein 90
ilvB	tr|T1RI34|ilvB	0.816	0.476	0.591	FALSE	FALSE	TRUE	4	Acetolactate synthase large subunit

inorganic, emb, Naga_100030 g25	tr|W7TN19|inorganic	2.85	0.113	0.278	FALSE	FALSE	TRUE	1	H+-translocating pyrophosphatase family
LACS	tr|G9BBC7|LACS	1.42	0.177	0.324	FALSE	FALSE	FALSE	0	Long-chain acyl-coenzyme A synthetase
LHC26	tr|W7UAI7|LHC26	0.961	0.372	0.501	FALSE	FALSE	FALSE	0	Light-harvesting protein
LHCA1	tr|W7T8I0|Naga_100641g3	1.03	0.299	0.446	FALSE	FALSE	FALSE	0	Light-harvesting protein
LHCP28	tr|W7TZB5|LHCP28	0.494	0.662	0.741	FALSE	FALSE	FALSE	0	Light-harvesting protein
LHCP5	tr|W7TCK1|LHCP5	2.47	0.0231	0.148	FALSE	FALSE	FALSE	0	Chloroplast light harvesting protein isoform 4
Lhcv3	tr|W7TRI0|Lhcv3	1.32	0.2	0.344	FALSE	FALSE	FALSE	0	Light-harvesting protein
MCAT	tr|S5VRZ9|MCAT	0.934	0.373	0.501	FALSE	FALSE	FALSE	0	Malonyl-:acp transacylase
nad11	tr|A0A023PJZ1|nad11	3.25	0.0114	0.112	FALSE	FALSE	TRUE	1	NADH dehydrogenase subunit 11
nad7	tr|A0A023PL86|nad7	2.3	0.0615	0.22	FALSE	FALSE	TRUE	1	NADH dehydrogenase subunit 7
nad9	tr|T1R893|nad9	2.42	0.0319	0.171	FALSE	FALSE	TRUE	1	NADH dehydrogenase subunit 9
Naga_100001 g128	tr|W7TKJ5|Naga_100001g128	0.528	0.636	0.721	FALSE	FALSE	FALSE	0	Transketolase

Naga_100001 g147	tr|W7TKL4|Naga_100001g147	1.81	0.127	0.282	FALSE	FALSE	FALSE	0	Cytochrome c oxidase subunit vb
Naga_100001 g183	tr|W7U1D3|Naga_100001g183	2.43	0.0618	0.22	FALSE	FALSE	TRUE	2	Protein arginine serine-rich 45
Naga_100001 g189	tr|W7U259|Naga_100001g189	2.36	0.0309	0.168	FALSE	FALSE	FALSE	0	Nucleoside diphosphate kinase
Naga_100001 g208	tr|W7U229|Naga_100001g208	1.95	0.0808	0.235	FALSE	FALSE	TRUE	4	Peptidyl-prolyl cis-trans isomerase
Naga_100001 g41	tr|W7U263|Naga_100001g41	1.62	0.169	0.312	FALSE	FALSE	FALSE	0	Heat shock protein 70
Naga_100001 g58	tr|W7U208|Naga_100001g58	3.14	0.0295	0.163	FALSE	FALSE	TRUE	1	Glyceraldehyde-3-phosphate dehydrogenase
Naga_100002 g111	tr|W7U788|Naga_100002g111	1.59	0.12	0.281	FALSE	FALSE	FALSE	0	Mitochondrial trna import complex
Naga_100002 g147	tr|W7TNH0|Naga_100002g147	-0.442	0.779	0.837	FALSE	FALSE	TRUE	3	Pyruvate carboxylase
Naga_100002 g172	tr|W7TNX9|Naga_100002g172	3.52	0.0263	0.159	FALSE	FALSE	TRUE	1	Acyl-dehydrogenase
Naga_100002 g173	tr|W7TRD5|Naga_100002g173	1.89	0.0707	0.224	FALSE	FALSE	FALSE	0	3-oxoacyl-[acyl-carrier-protein] synthase

Naga_100002 g55	tr|W7TYV8|Naga_100002g55	1.86	0.126	0.282	FALSE	FALSE	TRUE	1	Vacuolar (H+)-ATPase G subunit
Naga_100003 g103	tr|W7UBF9|Naga_100003g103	2.15	0.0702	0.224	FALSE	FALSE	FALSE	0	Vacuolar h+-atpase a subunit
Naga_100003 g126	tr|W7TSL6|Naga_100003g126	-0.639	0.678	0.751	FALSE	FALSE	TRUE	3	60s ribosomal protein l13a
Naga_100003 g133	tr|W7UBV8|Naga_100003g133	1.89	0.133	0.286	FALSE	FALSE	TRUE	1	Inosine-5-monophosphate dehydrogenase
Naga_100003 g157	tr|W7TVY6|Naga_100003g157	3.22	0.00781	0.103	FALSE	FALSE	FALSE	0	Glucose-6-phosphate isomerase
Naga_100003 g173	tr|W7U360|Naga_100003g173	1.63	0.161	0.312	FALSE	FALSE	FALSE	0	Atp-dependent metalloprotease
Naga_100003 g177	tr|W7TVZ5|Naga_100003g177	2.55	0.0686	0.224	FALSE	FALSE	TRUE	1	Heat shock protein 70
Naga_100003 g62	tr|W7TSQ3|Naga_100003g62	1.25	0.227	0.369	FALSE	FALSE	TRUE	4	Fumarate hydratase
Naga_100003 g67	tr|W7TSY3|Naga_100003g67	3.15	0.012	0.112	FALSE	FALSE	FALSE	0	Aconitate mitochondrial
Naga_100003 g69	tr|W7UC18|Naga_100003g69	2.78	0.0209	0.147	FALSE	FALSE	TRUE	2	Phosphoribosylformylglycinamidine synthase

Naga_100003g83	tr|W7UC09|Naga_100003g83	1.2	0.266	0.407	FALSE	FALSE	FALSE	0	Porphobilinogen deaminase
Naga_100004g111	tr|W7U649|Naga_100004g111	1.33	0.214	0.36	FALSE	FALSE	FALSE	0	Thioredoxin f
Naga_100004g135	tr|W7TM71|Naga_100004g135	1.85	0.124	0.282	FALSE	FALSE	TRUE	3	Ferredoxin
Naga_100004g79	tr|W7TQ45|Naga_100004g79	1.51	0.195	0.336	FALSE	FALSE	TRUE	4	Glutaredoxin 2
Naga_100005g139	tr|W7THB6|Naga_100005g139	1.96	0.0804	0.235	FALSE	FALSE	TRUE	4	Vacuolar transporter chaperone 4
Naga_100005g25	tr|W7TYA6|Naga_100005g25	3.2	0.0291	0.163	FALSE	FALSE	TRUE	1	Photosystem ii 11 kd protein
Naga_100005g46	tr|W7TEP7|Naga_100005g46	1.17	0.278	0.42	FALSE	FALSE	FALSE	0	Histone h2b
Naga_100005g52	tr|W7TPN7|Naga_100005g52	1.06	0.317	0.453	FALSE	FALSE	FALSE	0	Adp atp
Naga_100005g68	tr|W7TYC1|Naga_100005g68	1.27	0.219	0.366	FALSE	FALSE	FALSE	0	Short-chain dehydrogenase reductase acting with nad or nadp as acceptor
Naga_100005g83	tr|W7TXT5|Naga_100005g83	2.15	0.0635	0.221	FALSE	FALSE	TRUE	1	ATP-dependent Clp protease

									proteolytic subunit
Naga_100006g57	tr|W7TTR6|Naga_100006g57	-0.482	0.643	0.727	FALSE	FALSE	TRUE	2	Dnaj-like sec63
Naga_100006g64	tr|W7U194|Naga_100006g64	1.53	0.164	0.312	FALSE	FALSE	FALSE	0	Delta-1-pyrroline-5-carboxylate synthetase
Naga_100006g89, HTPG	tr|W7U9H6|Naga_100006g89	1.13	0.314	0.453	FALSE	FALSE	FALSE	0	Heat shock protein 90
Naga_100006g94	gi|553191250|ref|XP_005855348.1|	1.48	0.157	0.31	FALSE	FALSE	TRUE	3	spermidine synthase
Naga_100007g107	tr|W7U882|Naga_100007g107	0.442	0.698	0.757	FALSE	FALSE	FALSE	0	Rna binding s1 domain protein
Naga_100007g70	tr|W7U8K2|Naga_100007g70	2.56	0.0484	0.205	FALSE	FALSE	FALSE	0	Clathrin heavy chain
Naga_100008g4	tr|W7TPQ0|Naga_100008g4	2.78	0.0509	0.208	FALSE	FALSE	TRUE	1	Atp synthase gamma
Naga_100009g12	tr|W7TN92|Naga_100009g12	3.42	0.00405	0.0933	FALSE	FALSE	TRUE	2	6-phosphogluconate dehydrogenase, decarboxylating
Naga_100009g3	tr|W7TYG7|Naga_100009g3	-1.02	0.455	0.572	FALSE	FALSE	TRUE	3	Beta-tubulin

Naga_100009g63	tr|W7U6Y6|Naga_100009g63	0.248	0.802	0.852	FALSE	FALSE	FALSE	0	Eukaryotic translation initiation factor 5A
Naga_100009g67	tr|W7TR29|Naga_100009g67	2	0.0846	0.239	FALSE	FALSE	FALSE	0	Enolase
Naga_100009g84	tr|W7U6S0|Naga_100009g84	1.72	0.231	0.371	FALSE	FALSE	TRUE	1	60s acidic ribosomal protein p0
Naga_100010g11	gi|553190821|ref|XP_005855269.1|	1.88	0.12	0.281	FALSE	FALSE	TRUE	2	phosphoribosylaminoimidazolecarboxamide formyltransferase / IMP cyclohydrolase
Naga_100010g22	tr|W7TJF5|Naga_100010g22	0.855	0.399	0.525	FALSE	FALSE	FALSE	0	Nucleoredoxin
Naga_100010g75	tr|W7U3G1|Naga_100010g75	1.74	0.168	0.312	FALSE	FALSE	TRUE	3	Peptide methionine sulfoxide reductase b5
Naga_100010g85	tr|W7TMD6|Naga_100010g85	3.23	0.0162	0.127	FALSE	FALSE	TRUE	3	Mitochondrial phosphate transporter
Naga_100010g88	tr|W7U2S1|Naga_100010g88	0.251	0.819	0.865	FALSE	FALSE	FALSE	0	Ribosomal protein s16
Naga_100011g18	tr|W7T7M9|Naga_100011g18	1.83	0.139	0.293	FALSE	FALSE	TRUE	3	T-complex protein 1 subunit delta

Naga_100011g26	tr|W7TPX6|Naga_100011g26	2.36	0.0428	0.199	FALSE	FALSE	TRUE	1	Phosphoglycerate mutase
Naga_100011g29	tr|W7TG29|Naga_100011g29	2.66	0.028	0.163	FALSE	FALSE	TRUE	1	Tic22-like protein
Naga_100011g39	tr|W7TG62|Naga_100011g39	3.85	0.00609	0.103	FALSE	FALSE	TRUE	1	H-or na-translocating f-v-type and a-type atpase (F-atpase) superfamily
Naga_100012g22	gi|553183167|ref|XP_005853851.1|	1.68	0.138	0.293	FALSE	FALSE	TRUE	2	small nuclear ribonucleoprotein D1
Naga_100012g76	tr|W7TDH0|Naga_100012g76	1.92	0.183	0.327	FALSE	FALSE	TRUE	3	40s ribosomal protein
Naga_100013g72	tr|W7U3Y9|Naga_100013g72	3.3	0.00762	0.103	FALSE	FALSE	TRUE	2	Nicotinamide nucleotide transhydrogenase
Naga_100013g96, NGA_0373902	tr|W7UCP4|Naga_100013g96	2.54	0.0348	0.179	FALSE	FALSE	TRUE	2	Malate synthase a
Naga_100014g18, Naga_100014g18	tr|W7TRI5|Naga_100014g18	2.92	0.0447	0.199	FALSE	FALSE	TRUE	1	Phosphoglycerate kinase
Naga_100014g51	tr|W7U0I2|Naga_100014g51	0.528	0.631	0.719	FALSE	FALSE	FALSE	0	Ribosomal protein l12

Naga_100014g55	tr|W7TRK7|Naga_100014g55	2.4	0.0383	0.19	FALSE	FALSE	TRUE	3	Coatomer subunit alpha
Naga_100014g7	tr|W7TGE1|Naga_100014g7	-0.161	0.872	0.909	FALSE	FALSE	TRUE	1	Cupin 4 family protein
Naga_100015g11	tr|W7U4C6|Naga_100015g11	1.91	0.0897	0.246	FALSE	FALSE	FALSE	0	Citrate synthase
Naga_100015g48	tr|W7U4G7|Naga_100015g48	0.0648	0.951	0.964	FALSE	FALSE	FALSE	0	60S ribosomal protein L18
Naga_100016g24	tr|W7TG91|Naga_100016g24	1.25	0.227	0.369	FALSE	FALSE	FALSE	0	ATP synthase subunit beta
Naga_100016g30	tr|I2CPE6|NGATSA_2003000	2.15	0.0656	0.222	FALSE	FALSE	TRUE	2	Uncharacterized protein
Naga_100016g46	tr|W7TXP8|Naga_100016g46	1.13	0.319	0.453	FALSE	FALSE	FALSE	0	40S ribosomal protein S8
Naga_100016g48	tr|W7TDQ4|Naga_100016g48	-1.46	0.286	0.429	FALSE	FALSE	TRUE	4	Calreticulin
Naga_100016g59	tr|W7TNT0|Naga_100016g59	1.5	0.146	0.299	FALSE	FALSE	FALSE	0	ATPase, F0 complex, subunit B, mitochondrial
Naga_100016g72	tr|W7TXQ6|Naga_100016g72	2.73	0.0199	0.146	FALSE	FALSE	FALSE	0	Peptidyl-prolyl cis-trans isomerase

Naga_100017g18	tr|W7U1W1|Naga_100017g18	1.84	0.19	0.333	FALSE	FALSE	TRUE	1	60s ribosomal protein l11
Naga_100017g20	tr|W7U1X5|Naga_100017g20	2.01	0.121	0.281	FALSE	FALSE	TRUE	1	Transaldolase
Naga_100017g24	tr|W7U1V7|Naga_100017g24	0.0457	0.974	0.978	FALSE	FALSE	TRUE	3	Stress-inducible protein sti1
Naga_100017g36	tr|W7UAJ2|Naga_100017g36	-0.578	0.676	0.75	FALSE	FALSE	TRUE	2	Small GTP-binding protein domain protein
Naga_100019g53	tr|W7T4L1|Naga_100019g53	1.04	0.303	0.446	FALSE	FALSE	FALSE	0	Histone H2A
Naga_100019g59	tr|W7TM34|Naga_100019g59	0.635	0.583	0.688	FALSE	FALSE	TRUE	2	Hypersensitive-induced response protein
Naga_100019g64	tr|W7T3J7|Naga_100019g64	0.638	0.554	0.662	FALSE	FALSE	FALSE	0	Elongation factor 1-alpha
Naga_100020g11	tr|W7TLA3|Naga_100020g11	1.28	0.228	0.369	FALSE	FALSE	FALSE	0	Prohibitin
Naga_100020g47	tr|W7TD69|Naga_100020g47	1.55	0.142	0.295	FALSE	FALSE	FALSE	0	Myo-inositol 2-dehydrogenase
Naga_100020g62	tr|W7TD66|Naga_100020g62	3.74	0.00279	0.0913	FALSE	FALSE	TRUE	4	Peptidyl-prolyl cis-trans isomerase

Naga_100020g64	tr|W7TUE3|Naga_100020g64	3.13	0.0111	0.112	FALSE	FALSE	TRUE	3	Urease accessory protein ureg
Naga_100021g54	tr|W7TZI5|Naga_100021g54	0.291	0.79	0.842	FALSE	FALSE	TRUE	3	Armadillo-like helical
Naga_100021g68	tr|W7TI88|Naga_100021g68	3.16	0.00869	0.103	FALSE	FALSE	TRUE	3	Phosphoadenosine phosphosulfate reductase
Naga_100021g70	tr|W7TFK4|Naga_100021g70	0.885	0.54	0.647	FALSE	FALSE	TRUE	1	Alpha tubulin 1
Naga_100021g72	tr|W7TYX5|Naga_100021g72	4.47	0.00154	0.0571	FALSE	FALSE	TRUE	1	Glycine cleavage system regulatory protein
Naga_100022g39	tr|W7TUA2|Naga_100022g39	1.88	0.0903	0.246	FALSE	FALSE	FALSE	0	Enolase
Naga_100022g53	tr|W7U1U2|Naga_100022g53	2.35	0.0348	0.179	FALSE	FALSE	FALSE	0	Isocitrate dehydrogenase [NADP]
Naga_100023g44	tr|W7TQJ5|Naga_100023g44	1.6	0.114	0.279	FALSE	FALSE	TRUE	4	Chaperonin containing tcp1 theta subunit
Naga_100024g24, NGA_0404400	tr|W7U8G3|Naga_100024g24	1.67	0.132	0.286	FALSE	FALSE	FALSE	0	Atp-citrate synthase
Naga_100024g37, Naga_100024	tr|W7U814|Naga_100024g37	1.69	0.145	0.299	FALSE	FALSE	TRUE	3	Succinate dehydrogenase

g37, Naga_100024 g37									flavoprotein subunit
Naga_100024 g60	tr|W7TSA3|Naga_100024g60	0.6	0.61	0.71	FALSE	FALSE	TRUE	3	Rab11 family gtpase
Naga_100024 g61	tr|W7TSC4|Naga_100024g61	0.855	0.451	0.568	FALSE	FALSE	TRUE	4	Inositol-3-phosphate synthase
Naga_100025 g47	tr|W7U0R4|Naga_100025g47	0.63	0.536	0.644	FALSE	FALSE	FALSE	0	Ubiquitin ribosomal protein s27ae fusion protein
Naga_100026 g22	tr|W7TW72|Naga_100026g22	0.651	0.595	0.698	FALSE	FALSE	TRUE	3	60s ribosomal protein l24
Naga_100026 g40	tr|W7TWA9|Naga_100026g40	1.9	0.122	0.281	FALSE	FALSE	FALSE	0	14-3-3-like protein
Naga_100027 g34	tr|W7TUK8|Naga_100027g34	0.867	0.468	0.584	FALSE	FALSE	FALSE	0	Ribosomal protein l14
Naga_100029 g32	tr|W7TSX0|Naga_100029g32	1.83	0.122	0.281	FALSE	FALSE	FALSE	0	Plastidic atp adp transporter
Naga_100030 g5	tr|W7TTN9|Naga_100030g5	3.47	0.0291	0.163	FALSE	FALSE	TRUE	1	Chlorophyll A-B binding protein
Naga_100031 g28	tr|W7U0D4|Naga_100031g28	2.58	0.0477	0.205	FALSE	FALSE	TRUE	1	Succinyl-ligase subunit mitochondrial

Naga_100031g39	tr|W7TS51|Naga_100031g39	-0.67	0.689	0.755	FALSE	FALSE	TRUE	1	40s ribosomal protein s3-3
Naga_100032g3	tr|W7TXZ6|Naga_100032g3	-0.0492	0.975	0.978	FALSE	FALSE	TRUE	2	40S ribosomal protein S3a
Naga_100032g30	tr|W7TY00|Naga_100032g30	0.821	0.447	0.565	FALSE	FALSE	TRUE	2	Coatomer subunit beta
Naga_100032g40	tr|W7THF5|Naga_100032g40	1.11	0.313	0.453	FALSE	FALSE	FALSE	0	Ribosomal protein L4 domain protein
Naga_100034g18	tr|W7TKT1|Naga_100034g18	0.703	0.511	0.62	FALSE	FALSE	FALSE	0	60s ribosomal protein l18a
Naga_100035g25	tr|W7UAU3|Naga_100035g25	2.18	0.0708	0.224	FALSE	FALSE	TRUE	3	Homoserine kinase
Naga_100037g12	tr|W7TF53|Naga_100037g12	3.09	0.0447	0.199	FALSE	FALSE	TRUE	1	3-oxoacyl-(Acyl-carrier-protein) reductase
Naga_100037g14	tr|W7THS5|Naga_100037g14	2.84	0.0559	0.213	FALSE	FALSE	TRUE	1	Cyclophilin-like peptidyl-prolyl cis-trans isomerase
Naga_100038g29	tr|W7TUP5|Naga_100038g29	1.5	0.149	0.303	FALSE	FALSE	FALSE	0	Atp synthase subunit delta
Naga_100038g3	tr|W7U315|Naga_100038g3	0.928	0.417	0.542	FALSE	FALSE	TRUE	1	Dead-box atp-dependent rna helicase

Naga_100038g9	tr|W7TMH8|Naga_100038g9	2.97	0.021	0.147	FALSE	FALSE	FALSE	0	Udp-sulfoquinovose synthase
Naga_100040g16	tr|W7TUN3|Naga_100040g16	1.31	0.272	0.415	FALSE	FALSE	TRUE	3	Sulfite ferredoxin dependent
Naga_100040g42	tr|W7TTU9|Naga_100040g42	0.272	0.824	0.869	FALSE	FALSE	TRUE	2	Atp-dependent chaperone
Naga_100041g2	tr|W7TL25|Naga_100041g2	0.82	0.423	0.543	FALSE	FALSE	FALSE	0	Elongation factor 1
Naga_100041g31	tr|W7T232|Naga_100041g31	1.02	0.319	0.453	FALSE	FALSE	FALSE	0	Adenosylhomocysteinase
Naga_100041g46	ANT70525.1	2.97	0.0141	0.121	FALSE	FALSE	TRUE	2	violaxanthin de-epoxidase
Naga_100042g43	tr|W7U8W3|Naga_100042g43	1.89	0.0846	0.239	FALSE	FALSE	FALSE	0	Nadp-dependent glyceraldehyde-3-phosphate dehydrogenase
Naga_100044g11	tr|W7U5I6|Naga_100044g11	1.95	0.127	0.282	FALSE	FALSE	TRUE	1	40s ribosomal protein s15a
Naga_100045g24	tr|W7TK84|Naga_100045g24	1.5	0.192	0.333	FALSE	FALSE	FALSE	0	ATPase, AAA-type, core
Naga_100047g7	tr|W7TB36|Naga_100047g7	2.56	0.0657	0.222	FALSE	FALSE	TRUE	3	Succinyl-CoA ligase subunit beta

Naga_100048 g7	tr|W7TZM5|Naga_100048g7	3.19	0.00613	0.103	FALSE	FALSE	TRUE	3	Aldehyde dehydrogenase
Naga_100048 g8	tr|W7TZ20|Naga_100048g8	1.11	0.301	0.446	FALSE	FALSE	TRUE	1	Homoaconitate hydratase family protein
Naga_100050 g29, Naga_100123 g11	tr|W7TR01|Naga_100050g29	1.49	0.302	0.446	FALSE	FALSE	FALSE	0	Histone H4
Naga_100050 g33, Naga_100722 g1, NGA_0455000, Naga_100605 g3	tr|W7UA52|Naga_100050g33	-2.58	0.178	0.324	FALSE	FALSE	TRUE	1	Histone H3
Naga_100050 g34, Naga_100012 g58	tr|W7U9K9|Naga_100050g34	2.3	0.0681	0.224	FALSE	FALSE	FALSE	0	Histone H2A
Naga_100050 g39	tr|W7U1E9|Naga_100050g39	2.84	0.0463	0.202	FALSE	FALSE	TRUE	1	Uncharacterized protein
Naga_100051 g29	tr|W7TM73|Naga_100051g29	4.51	0.00572	0.103	FALSE	FALSE	TRUE	1	Superoxide dismutase
Naga_100054 g19	tr|K8YNR1|AP1B1	1.39	0.26	0.405	FALSE	FALSE	TRUE	4	AP complex subunit beta

Naga_100054g8	tr|W7TYZ7|Naga_100054g8	-1.1	0.366	0.495	FALSE	FALSE	TRUE	3	Atp dependent rna helicase
Naga_100056g12, Naga_100056g12	tr|W7TU57|Naga_100056g12	1.24	0.35	0.478	FALSE	FALSE	FALSE	0	Phosphoenolpyruvate carboxykinase
Naga_100056g15	tr|W7TJ16|Naga_100056g15	1.47	0.16	0.312	FALSE	FALSE	FALSE	0	Light-harvesting protein
Naga_100056g25	tr|W7U2J3|Naga_100056g25	3.49	0.0233	0.148	FALSE	FALSE	TRUE	1	Glutamine synthetase
Naga_100059g16	tr|W7TNL3|Naga_100059g16	-0.831	0.625	0.716	FALSE	FALSE	TRUE	3	40s ribosomal protein s26
Naga_100059g26	tr|W7TVV4|Naga_100059g26	1.78	0.158	0.31	FALSE	FALSE	FALSE	0	Guanine nucleotide binding protein
Naga_100061g16	tr|W7T2I2|Naga_100061g16	1.75	0.112	0.278	FALSE	FALSE	TRUE	3	Carboxyl transferase
Naga_100061g24, Naga_100003g61	tr|W7T2C3|Naga_100061g24	3.29	0.0109	0.112	FALSE	FALSE	TRUE	3	26s proteasome regulatory atpase rpt4
Naga_100061g9	tr|W7TKK2|Naga_100061g9	0.209	0.884	0.92	FALSE	FALSE	TRUE	2	40s ribosomal protein s23
Naga_100064g3	tr|W7T6I6|Naga_100064g3	1.66	0.125	0.282	FALSE	FALSE	TRUE	4	Methylenetetrahydrofolate dehydrogenase

Naga_100065 g10	tr|W7T5H8|Naga_100065g10	3.45	0.0142	0.121	FALSE	FALSE	TRUE	1	Phosphoglucomutase
Naga_100065 g23	tr|W7TE43|Naga_100065g23	1.65	0.139	0.294	FALSE	FALSE	FALSE	0	Leucyl aminopeptidase
Naga_100067 g28	tr|W7TFX4|Naga_100067g28	2.95	0.01	0.112	FALSE	FALSE	TRUE	3	Proteasome subunit alpha
Naga_100070 g2	tr|W7TMG4|Naga_100070g2	1.19	0.419	0.543	FALSE	FALSE	TRUE	3	30s ribosomal protein s15
Naga_100070 g24	tr|W7T4K9|Naga_100070g24	0.63	0.58	0.687	FALSE	FALSE	TRUE	3	Aminopeptidase puromycin sensitive
Naga_100076 g10, Naga_100078 g16, NGA_0671820	tr|W7TVR6|Naga_100076g10	-0.393	0.79	0.842	FALSE	FALSE	TRUE	2	Elongation factor ef-3
Naga_100076 g3	tr|W7TEW9|Naga_100076g3	3.2	0.0569	0.213	FALSE	FALSE	TRUE	1	Photosystem ii 12 kDa extrinsic protein
Naga_100078 g15, Naga_100076 g9	tr|W7TS20|Naga_100078g15	1.07	0.412	0.536	FALSE	FALSE	TRUE	2	Elongation factor 3
Naga_100079 g12	tr|W7TLT5|Naga_100079g12	2.82	0.0576	0.213	FALSE	FALSE	TRUE	1	Cysteine synthase

Naga_100079g2	tr|W7TIZ8|Naga_100079g2	1.62	0.163	0.312	FALSE	FALSE	TRUE	4	Propionyl-alpha subunit
Naga_100081g17	tr|W7T2Q2|Naga_100081g17	1.19	0.264	0.406	FALSE	FALSE	FALSE	0	Glyceraldehyde-3-phosphate dehydrogenase
Naga_100084g4	tr|W7TSQ4|Naga_100084g4	0.737	0.502	0.613	FALSE	FALSE	FALSE	0	S-adenosylmethionine synthase
Naga_100086g16	tr|W7TDA6|Naga_100086g16	1.57	0.129	0.282	FALSE	FALSE	FALSE	0	Pentapeptide repeat protein
Naga_100097g1	tr|I2CNZ7|NGATSA_3003400	2.51	0.0234	0.148	FALSE	FALSE	TRUE	3	Glutamate synthase
Naga_100097g2	tr|W7U2E4|Naga_100097g2	0.612	0.587	0.691	FALSE	FALSE	TRUE	3	Uncharacterized protein
Naga_100098g5	tr|W7TNK1|Naga_100098g5	2.84	0.0119	0.112	FALSE	FALSE	TRUE	3	Cell division protein
Naga_100099g18	tr|W7U484|Naga_100099g18	-1.08	0.31	0.453	FALSE	FALSE	FALSE	0	Ribosomal protein L15
Naga_100099g23	tr|W7TVG2|Naga_100099g23	1.32	0.208	0.353	FALSE	FALSE	FALSE	0	Uncharacterized protein
Naga_100100g13	tr|W7TTE5|Naga_100100g13	0.948	0.392	0.523	FALSE	FALSE	FALSE	0	Gtp-binding nuclear protein ran

Naga_100102g18, Naga_100214g8	tr|W7TRP0|Naga_100102g18	1.11	0.326	0.458	FALSE	FALSE	FALSE	0	Eukaryotic initiation factor 4a
Naga_100102g2, NGA_0449600	tr|W7TRP6|Naga_100102g2	1.16	0.275	0.418	FALSE	FALSE	FALSE	0	Heat shock protein 70
Naga_100103g9	tr|W7TLH5|Naga_100103g9	1.99	0.185	0.327	FALSE	FALSE	TRUE	1	Ribosomal protein s20
Naga_100106g2	tr|W7TUQ1|Naga_100106g2	0.0488	0.973	0.978	FALSE	FALSE	TRUE	1	40S ribosomal protein S4
Naga_100108g3	tr|W7TKA6|Naga_100108g3	1.79	0.263	0.406	FALSE	FALSE	TRUE	1	40S ribosomal protein SA
Naga_100108g6	tr|W7TL56|Naga_100108g6	2.03	0.0614	0.22	FALSE	FALSE	TRUE	1	Ribonucleoprotein LSM
Naga_100113g12	tr|W7TCB7|Naga_100113g12	0.622	0.596	0.698	FALSE	FALSE	FALSE	0	40s ribosomal protein s18
Naga_100113g20	tr|W7TUD0|Naga_100113g20	1.83	0.0935	0.249	FALSE	FALSE	FALSE	0	Vacuolar h+ atpase b subunit
Naga_100114g2	tr|W7TR43|Naga_100114g2	1.51	0.132	0.286	FALSE	FALSE	FALSE	0	Photosystem II stability/assembly factor
Naga_100117g8	tr|W7TVJ9|Naga_100117g8	2.94	0.0132	0.117	FALSE	FALSE	TRUE	2	2-oxoglutarate dehydrogenase e1 component

Naga_100118 g22	tr|W7T749|Naga_100118g22	2	0.0548	0.213	FALSE	FALSE	FALSE	0	Bacterioferritin comigratory protein
Naga_100119 g3, NGA_0477710	tr|W7TQ47|Naga_100119g3	3.47	0.00863	0.103	FALSE	FALSE	TRUE	4	Fructose-bisphosphate aldolase
Naga_100120 g1	tr|W7TPV3|Naga_100120g1	1.89	0.0975	0.256	FALSE	FALSE	FALSE	0	Cell division protein
Naga_100122 g10	tr|W7TFU2|Naga_100122g10	0.425	0.681	0.752	FALSE	FALSE	FALSE	0	Chaperonin
Naga_100124 g18	tr|W7TU78|Naga_100124g18	3.82	0.00601	0.103	FALSE	FALSE	TRUE	1	Phosphoribosylpyrophosphate synthetase
Naga_100125 g16	gi|553180480|ref|XP_005853411.1|	0.19	0.852	0.893	FALSE	FALSE	FALSE	0	large subunit ribosomal protein L27Ae
Naga_100129 g1	tr|W7UCU5|Naga_100129g1	1.03	0.34	0.471	FALSE	FALSE	FALSE	0	Poly binding protein 8
Naga_100131 g7	tr|W7U6U0|Naga_100131g7	1.64	0.184	0.327	FALSE	FALSE	TRUE	4	Apoptosis inducing factor
Naga_100149 g4	tr|W7U4E6|Naga_100149g4	-1	0.399	0.525	FALSE	FALSE	TRUE	2	Abc transporter g family member 7
Naga_100156 g9	tr|W7TKH1|Naga_100156g9	0.433	0.694	0.756	FALSE	FALSE	FALSE	0	Ribosomal protein L7A/L8

Naga_100164 g14	tr|W7TIS3|Naga_100164g14	2.78	0.0602	0.22	FALSE	FALSE	TRUE	1	Peroxiredoxin 1
Naga_100171 g15	tr|W7TC86|Naga_100171g15	0.996	0.375	0.501	FALSE	FALSE	FALSE	0	Protein transport protein sec61 subunit alpha
Naga_100171 g8	tr|W7TWB2|Naga_100171g8	1.94	0.0934	0.249	FALSE	FALSE	FALSE	0	Chaperonin Cpn60/TCP-1
Naga_100175 g2	tr|W7TED5|Naga_100175g2	2.51	0.0832	0.239	FALSE	FALSE	TRUE	1	RNA binding protein
Naga_100186 g6	tr|W7TXA2|Naga_100186g6	2.55	0.0201	0.146	FALSE	FALSE	TRUE	2	Proteasome subunit beta type
Naga_100187 g5	tr|W7TW63|Naga_100187g5	2.11	0.0728	0.225	FALSE	FALSE	TRUE	1	Trimeric LpxA
Naga_100187 g8	tr|W7TW67|Naga_100187g8	1.41	0.192	0.333	FALSE	FALSE	FALSE	0	Transitional endoplasmic reticulum atpase
Naga_100189 g5	tr|W7TSS0|Naga_100189g5	1.03	0.347	0.475	FALSE	FALSE	TRUE	2	Aspartate-semialdehyde dehydrogenase
Naga_100194 g2	ANT70526.1	3.75	0.00319	0.0933	FALSE	FALSE	TRUE	3	zeaxanthin epoxidase 1
Naga_100197 g3	tr|W7TLB7|Naga_100197g3	3.68	0.00701	0.103	FALSE	FALSE	TRUE	2	Pyruvate kinase
Naga_100207 g6	tr|W7THD6|Naga_100207g6	1.15	0.398	0.525	FALSE	FALSE	TRUE	1	Geranylgeranyl reductase

Naga_100208g4	tr|W7TPK9|Naga_100208g4	1.01	0.366	0.495	FALSE	FALSE	FALSE	0	Acetohydroxy acid isomeroreductase, catalytic
Naga_100228g7	tr|W7T0L9|Naga_100228g7	0.842	0.404	0.53	FALSE	FALSE	FALSE	0	RNA helicase,ATP-dependent, DEAD-box type
Naga_100244g5	tr|W7TNV1|Naga_100244g5	3.93	0.00399	0.0933	FALSE	FALSE	TRUE	1	Uncharacterized protein
Naga_100245g2	tr|W7T7S0|Naga_100245g2	2.24	0.0556	0.213	FALSE	FALSE	TRUE	1	Nucleotide-binding, alpha-beta plait
Naga_100257g1	tr|W7U445|Naga_100257g1	1.86	0.0751	0.228	FALSE	FALSE	FALSE	0	Glyceraldehyde-3-phosphate dehydrogenase
Naga_100268g2	gi|553180870|ref|XP_005853474.1|	3.05	0.0197	0.146	FALSE	FALSE	TRUE	3	6-phosphofructo-2-kinase / fructose-2,6-bisphosphatase
Naga_100273g6, NGA_0210100	tr|W7TJZ9|Naga_100273g6	1.86	0.0765	0.231	FALSE	FALSE	FALSE	0	Extrinsic protein in photosystem ii
Naga_100273g9	tr|W7TTD1|Naga_100273g9	1.79	0.0973	0.256	FALSE	FALSE	FALSE	0	Nucleoside diphosphate kinase
Naga_100307g4, NGA_0434010	tr|W7TLX9|Naga_100307g4	2.35	0.0497	0.205	FALSE	FALSE	TRUE	3	Mitochondrial inner membrane protein Mitofilin

Naga_100308g2, NGA_0607500	tr|W7TS11|Naga_100308g2	-0.093	0.932	0.957	FALSE	FALSE	TRUE	1	Ribosomal protein l5
Naga_100348g1	tr|W7TXQ4|Naga_100348g1	0.552	0.634	0.721	FALSE	FALSE	FALSE	0	Ribosomal protein S7
Naga_100356g1	tr|I2CNY3|NGATSA_3057400	0.259	0.838	0.88	FALSE	FALSE	TRUE	1	Hypoxia up-regulated 1
Naga_100385g2	tr|W7TK08|Naga_100385g2	1.51	0.153	0.305	FALSE	FALSE	FALSE	0	Acyl carrier protein
Naga_100410g3	tr|W7TJY6|Naga_100410g3	1.64	0.135	0.29	FALSE	FALSE	FALSE	0	Phosphoglycerate kinase
Naga_100419g4	tr|W7U2N9|Naga_100419g4	1.25	0.237	0.377	FALSE	FALSE	TRUE	2	Ornithine aminotransferase
Naga_100424g3	tr|W7TCV4|Naga_100424g3	0.122	0.921	0.95	FALSE	FALSE	TRUE	3	3-dehydroquinate synthase
Naga_100430g3	tr|W7T8M4|Naga_100430g3	0.0796	0.935	0.957	FALSE	FALSE	FALSE	0	Translationally controlled tumor protein
Naga_100466g3	tr|W7TLR9|Naga_100466g3	0.186	0.862	0.901	FALSE	FALSE	TRUE	2	Trifunctional enzyme subunit mitochondrial
Naga_100475g1	tr|W7TJS6|Naga_100475g1	0.842	0.399	0.525	FALSE	FALSE	FALSE	0	Uncharacterized protein

Naga_100529 g1	tr|W7TQA6|Naga_100529g1	2.21	0.111	0.278	FALSE	FALSE	TRUE	1	Fructokinase
Naga_100540 g2, RP-L13E	tr|W7TP63|Naga_100540g2	-0.259	0.814	0.863	FALSE	FALSE	FALSE	0	60s ribosomal protein l13
Naga_100594 g3	tr|W7TSZ8|Naga_100594g3	1.34	0.228	0.369	FALSE	FALSE	FALSE	0	Branched-chain alpha-keto acid dehydrogenase subunit
Naga_100638 g3	tr|W7TSR6|Naga_100638g3	0.885	0.48	0.593	FALSE	FALSE	TRUE	1	60s ribosomal protein l21-a
Naga_100638 g4	tr|W7T973|Naga_100638g4	0.85	0.425	0.543	FALSE	FALSE	FALSE	0	40s ribosomal protein s13
Naga_100665 g2	gi|553194809|ref|XP_005856032.1|	1.96	0.119	0.281	FALSE	FALSE	TRUE	1	coproporphyrinogen III oxidase
Naga_100667 g1	tr|W7T3X6|Naga_100667g1	0.0706	0.946	0.964	FALSE	FALSE	FALSE	0	60s ribosomal protein l3
Naga_100710 g1	tr|W7TQV0|Naga_100710g1	2.01	0.0717	0.224	FALSE	FALSE	TRUE	1	Elongation factor tu
Naga_100729 g1	gi|553185252|ref|XP_005854222.1|	1.46	0.168	0.312	FALSE	FALSE	FALSE	0	glutamate decarboxylase
Naga_100744 g1	gi|553190853|ref|XP_005855276.1|	0.794	0.622	0.715	FALSE	FALSE	TRUE	2	wos2 protein

Naga_100855g2	tr|W7TN59|Naga_100855g2	2.07	0.0653	0.222	FALSE	FALSE	FALSE	0	Malate dehydrogenase
Naga_100881g1	tr|W7TMV0|Naga_100881g1	2.33	0.0446	0.199	FALSE	FALSE	FALSE	0	Heat shock protein 101
Naga_100928g1	tr|W7TQR4|Naga_100928g1	1.13	0.339	0.471	FALSE	FALSE	FALSE	0	Atp-dependent rna helicase uap56
Naga_100967g1	tr|W7TSC3|Naga_100967g1	-0.063	0.949	0.964	FALSE	FALSE	FALSE	0	Beta-ig-h3 fasciclin
Naga_101003g2	tr|W7TBB8|Naga_101003g2	0.481	0.621	0.715	FALSE	FALSE	TRUE	4	Uncharacterized protein
Naga_101053g1	tr|I2CQQ0|NGATSA_3003200	1.43	0.169	0.312	FALSE	FALSE	FALSE	0	Uncharacterized protein
Naga_101070g1	tr|I2CQQ4|NGATSA_3003700	1.62	0.119	0.281	FALSE	FALSE	TRUE	3	Ras-related C3 botulinum toxin substrate 1
Naga_101137g1	tr|W7UCV3|Naga_101137g1	0.818	0.476	0.591	FALSE	FALSE	FALSE	0	Polyadenylate-binding protein
Naga_101273g1	tr|W7THF6|Naga_101273g1	1.82	0.166	0.312	FALSE	FALSE	TRUE	1	3-isopropylmalate dehydrogenase
Naga_101276g1	tr|W7T0G1|Naga_101276g1	-2.07	0.116	0.281	FALSE	FALSE	TRUE	4	Ribosomal protein l30
NAG-PR, ARGC	tr|W7TID0|NAG-PR	1.94	0.0839	0.239	FALSE	FALSE	TRUE	2	N-acetyl-gamma-

									glutamyl-phosphate reductase
NDA	tr|W7TLJ8|NDA	1.42	0.168	0.312	FALSE	FALSE	TRUE	4	Alternative nadh-dehydrogenase
NGA_0045802, Naga_100004g110	tr|K8Z8Q4|NGA_0045802	1.75	0.104	0.27	FALSE	FALSE	FALSE	0	Chaperonin 10
NGA_0094200, Naga_100006g87	tr|K8YQS9|NGA_0094200	1.8	0.108	0.276	FALSE	FALSE	FALSE	0	4-nitrophenyl phosphatase
NGA_0096400	tr|K8YQP9|NGA_0096400	2.49	0.117	0.281	FALSE	FALSE	TRUE	1	Nad-dependent epimerase dehydratase
NGA_0170500,oec	tr|K8Z905|NGA_0170500	3.57	0.0035	0.0933	FALSE	FALSE	TRUE	3	Uncharacterized protein
NGA_0189801, LHCP31	tr|K8YTS8|NGA_0189801	1.5	0.143	0.297	FALSE	FALSE	FALSE	0	Light-harvesting protein
NGA_0190001, Naga_100018g43	tr|K8YWQ7|NGA_0190001	2.77	0.0437	0.199	FALSE	FALSE	TRUE	3	Uncharacterized protein
NGA_0271420, Naga_100041g44, Naga_100051g23	tr|K8YVV3|NGA_0271420	1.7	0.11	0.278	FALSE	FALSE	TRUE	3	Uncharacterized protein
NGA_0361402, CHI	tr|K8YRK7|NGA_0361402	3.34	0.0118	0.112	FALSE	FALSE	TRUE	1	Uncharacterized protein

NGA_0366400, LHCP33	tr|K8YRV9|NGA_0366400	1.3	0.215	0.36	FALSE	FALSE	FALSE	0	Light-harvesting protein
NGA_0391400	gi|585100011|gb|EWM20418.1|	2.04	0.0719	0.224	FALSE	FALSE	FALSE	0	oxidoreductase
NGA_0448400, Naga_100004g11	tr|K8Z7F5|NGA_0448400	1.5	0.188	0.331	FALSE	FALSE	FALSE	0	Soul heme-binding protein
NGA_0501500, CLP	tr|K8Z7E0|NGA_0501500	2.28	0.0528	0.209	FALSE	FALSE	TRUE	1	ATP-dependent Clp protease proteolytic subunit
NGA_0504200, Naga_100612g2	tr|K8YVY4|NGA_0504200	0.425	0.671	0.749	FALSE	FALSE	FALSE	0	Glycine-rich rna-binding protein 4
NGA_0612301, Tim13	tr|K8YZ78|NGA_0612301	2.77	0.0227	0.148	FALSE	FALSE	TRUE	2	Mitochondrial protein translocase family
NGA_0635410, Naga_100151g7, NGA_0635700, Naga_100151g3	tr|K8YQ29|NGA_0635410	1.94	0.116	0.281	FALSE	FALSE	FALSE	0	H+-transporting ATPase
NGA_0699400, LHCP21	tr|K8YWB4|NGA_0699400	1.89	0.0771	0.231	FALSE	FALSE	FALSE	0	Light harvesting complex protein

NGA_2097200, Naga_100099g15	tr|K8Z221|NGA_2097200	2.66	0.0401	0.196	FALSE	FALSE	TRUE	1	Ammonium transporter
NGATSA_3001900	tr|I2CQN8|NGATSA_3001900	1.66	0.141	0.295	FALSE	FALSE	TRUE	4	Eukaryotic translation initiation factor isoform 1
NGATSA_3002800	gi|578896496|gb|AHI17198.1|	1.93	0.111	0.278	FALSE	FALSE	FALSE	0	acetyl-CoA carboxylase
NGATSA_3003100	tr|I2CQP8|NGATSA_3003100	3.12	0.0259	0.159	FALSE	FALSE	TRUE	1	Glycine dehydrogenase
NGATSA_3004500	tr|I2CQR0|NGATSA_3004500	1.15	0.316	0.453	FALSE	FALSE	FALSE	0	Glutamate-1-semialdehyde aminotransferase /glutamate-1-semialdehyde 21-aminomutase
NGATSA_3005800	tr|I2CQR9|NGATSA_3005800	2.52	0.0374	0.188	FALSE	FALSE	TRUE	2	GMP synthase (Glutamine-hydrolysing)
NGATSA_3016900	tr|I2CR44|NGATSA_3016900	3.28	0.0498	0.205	FALSE	FALSE	TRUE	1	Uncharacterized protein
NGATSA_3021000	tr|I2CP43|NGATSA_3021000	0.991	0.339	0.471	FALSE	FALSE	TRUE	4	T-complex protein 1 subunit beta
NGATSA_3021500	gi|585103939|gb|EWM23085.1|	0.692	0.51	0.62	FALSE	FALSE	TRUE	4	malic enzyme

NGATSA_3023700	tr|I2CP67|NGATSA_3023700	2.31	0.0521	0.209	FALSE	FALSE	TRUE	3	RuvB-like protein 1 (Pontin 52)
NGATSA_3026900	gi|585105860|gb|EWM24426.1|	1.92	0.168	0.312	FALSE	FALSE	TRUE	1	asf sf2-like pre-mrna splicing factor srp31
NGATSA_3041300	tr|I2CPH3|NGATSA_3041300	-0.308	0.782	0.838	FALSE	FALSE	FALSE	0	Large subunit ribosomal protein L8e
NUO10	tr|W7UBY9|NUO10	1.47	0.167	0.312	FALSE	FALSE	TRUE	3	Nadh ubiquinone
PAO2	tr|W7TRP5|PAO2	1.69	0.109	0.278	FALSE	FALSE	TRUE	4	Pheophorbide a oxygenase
PDH	tr|W7TYT3|PDH	0.478	0.645	0.727	FALSE	FALSE	FALSE	0	Transketolase
PDS	tr|W7TCB4|PDS	1.31	0.285	0.429	FALSE	FALSE	TRUE	2	Phytoene desaturase
PEFG	tr|W7THY0|PEFG	0.00956	0.993	0.993	FALSE	FALSE	FALSE	0	Elongation factor G, mitochondrial
petA	tr|T1RJB3|petA	2.74	0.022	0.148	FALSE	FALSE	FALSE	0	Apocytochrome f
petB	tr|K9ZX12|petB	2.52	0.0227	0.148	FALSE	FALSE	FALSE	0	Cytochrome b6
PETC	tr|W7TXJ0|PETC	3.18	0.0415	0.199	FALSE	FALSE	TRUE	1	Cytochrome b6-f complex iron-sulfur subunit
petD	tr|T1RJJ4|petD	3.89	0.00691	0.103	FALSE	FALSE	TRUE	2	Cytochrome b6-f complex subunit 4
petF	tr|K9ZV85|petF	4.8	0.00866	0.103	FALSE	FALSE	TRUE	1	Ferredoxin

PETH, Naga_100732g2	tr|K8YXT7|PETH	1.95	0.0904	0.246	FALSE	FALSE	FALSE	0	Ferredoxin--NADP+ reductase
petJ	tr|T1RJX4|petJ	1.94	0.0637	0.221	FALSE	FALSE	FALSE	0	Cytochrome c6
PPC	tr|W7TGA1|PPC	3.81	0.00429	0.0934	FALSE	FALSE	TRUE	2	Phosphoenolpyruvate carboxylase
PPX	tr|W7TSK2|PPX	0.84	0.481	0.593	FALSE	FALSE	TRUE	1	Protoporphyrinogen oxidase
PRK	tr|W7TMN7|PRK	0.856	0.41	0.535	FALSE	FALSE	FALSE	0	Phosphoribulokinase
psaA	tr|T1RJP7|psaA	1.39	0.18	0.324	FALSE	FALSE	FALSE	0	Photosystem I P700 chlorophyll a apoprotein A1
psaB	tr|T1RJ53|psaB	1.6	0.128	0.282	FALSE	FALSE	FALSE	0	P700 chlorophyll a apoprotein A2
psaC	tr|T1RJY1|psaC	4.38	0.00848	0.103	FALSE	FALSE	TRUE	1	Photosystem I iron-sulfur center
psaD	tr|T1RJN5|psaD	2.75	0.0288	0.163	FALSE	FALSE	FALSE	0	Photosystem I reaction center subunit II
psaE	tr|T1RJ36|psaE	2.39	0.0324	0.171	FALSE	FALSE	FALSE	0	Photosystem I reaction center subunit IV
psaF	tr|T1RIP4|psaF	4.24	0.0127	0.116	FALSE	FALSE	TRUE	1	Photosystem I subunit III
psaL	tr|T1RJZ1|psaL	2.21	0.0488	0.205	FALSE	FALSE	FALSE	0	Photosystem I reaction center subunit XI
psbA	tr|T1RJV8|psbA	1.52	0.151	0.305	FALSE	FALSE	FALSE	0	Photosystem II protein D1

psbB	tr|T1RJL7|psbB	1.92	0.0808	0.235	FALSE	FALSE	FALSE	0	Photosystem II CP47 reaction center protein
psbC	tr|T1RJG1|psbC	2.48	0.0361	0.184	FALSE	FALSE	FALSE	0	Photosystem II CP43 reaction center protein
psbD	tr|T1RIM2|psbD	1.54	0.156	0.309	FALSE	FALSE	FALSE	0	Photosystem II D2 protein
psbE	tr|T1RHR2|psbE	3.17	0.0638	0.221	FALSE	FALSE	TRUE	1	Cytochrome b559 subunit alpha
psbO	tr|W7TVN8|psbO	1.49	0.167	0.312	FALSE	FALSE	FALSE	0	Oxygen-evolving enhancer protein
psbV	tr|T1RJ27|psbV	1.77	0.087	0.242	FALSE	FALSE	FALSE	0	Cytochrome c-550
PTSI	tr|W7U015|Naga_100469g1	0.431	0.725	0.781	FALSE	FALSE	TRUE	3	Phosphate dikinase
PYK	tr|W7TRP2|PYK	1.59	0.146	0.299	FALSE	FALSE	FALSE	0	Pyruvate kinase
Rab1B, Naga_100010g23	tr|W7TST3|Rab1B	0.428	0.689	0.755	FALSE	FALSE	TRUE	3	Rab family gtpase
rbcL, dbj	tr|T1RJ90|rbcL	0.762	0.467	0.584	FALSE	FALSE	FALSE	0	Ribulose bisphosphate carboxylase large chain
rbcS	tr|A0A023PMA5|rbcS	0.675	0.521	0.63	FALSE	FALSE	FALSE	0	Ribulose bisphosphate carboxlyase small chain

RPE	tr|W7TD18|RPE	3.22	0.00723	0.103	FALSE	FALSE	FALSE	0	Ribulose-phosphate 3-epimerase
RPI	tr|K8Z9G7|RPIA	1.41	0.184	0.327	FALSE	FALSE	FALSE	0	Ribose 5-phosphate isomerase A
rpl1	tr|T1RJV1|rpl1	1.53	0.229	0.37	FALSE	FALSE	TRUE	1	50S ribosomal protein L1, chloroplastic
rpl12	tr|T1RJ33|rpl12	-0.0597	0.952	0.964	FALSE	FALSE	FALSE	0	50S ribosomal protein L12, chloroplastic
rpl13	tr|T1RJ81|rpl13	1.18	0.314	0.453	FALSE	FALSE	FALSE	0	50S ribosomal protein L13, chloroplastic
rpl22	tr|T1RJ80|rpl22	0.504	0.63	0.719	FALSE	FALSE	FALSE	0	50S ribosomal protein L22, chloroplastic
rpl23	tr|T1RJK6|rpl23	-0.438	0.688	0.755	FALSE	FALSE	TRUE	2	50S ribosomal protein L23, chloroplastic
rpl4	tr|T1RJ94|rpl4	0.166	0.905	0.939	FALSE	FALSE	TRUE	2	50S ribosomal protein L4
rpl5	tr|T1RJ87|rpl5	1.45	0.24	0.381	FALSE	FALSE	TRUE	1	50S ribosomal protein L5, chloroplastic
rpl6	tr|T1RJ48|rpl6	1.26	0.364	0.495	FALSE	FALSE	TRUE	1	50S ribosomal protein L6, chloroplastic
rps1	tr|W7TJ69|rps1	2.38	0.154	0.305	FALSE	FALSE	TRUE	1	30s ribosomal protein s1

rps10	tr|T1RHU1|rps10	-0.114	0.918	0.949	FALSE	FALSE	FALSE	0	30S ribosomal protein S10, chloroplastic
rps13	tr|T1RJU6|rps13	0.573	0.61	0.71	FALSE	FALSE	FALSE	0	30S ribosomal protein S13, chloroplastic
rps2	tr|T1RJU9|rps2	-1.67	0.179	0.324	FALSE	FALSE	TRUE	4	30S ribosomal protein S2, chloroplastic
RP-S2E, Naga_100062g29	tr|K8Z375|RP-S2E	0.516	0.619	0.715	FALSE	FALSE	FALSE	0	Small subunit ribosomal protein S2e
rps3	tr|A0A023PMI0|rps3	-0.491	0.658	0.739	FALSE	FALSE	TRUE	4	30S ribosomal protein S3, chloroplastic
rps5	tr|A0A023PLR6|rps5	0.706	0.618	0.715	FALSE	FALSE	TRUE	2	30S ribosomal protein S5, chloroplastic
rps6	tr|T1RIC4|rps6	-0.0502	0.967	0.977	FALSE	FALSE	TRUE	3	30S ribosomal protein S6, chloroplastic
rps9	tr|T1RJK0|rps9	1.03	0.428	0.544	FALSE	FALSE	TRUE	3	30S ribosomal protein S9, chloroplastic
RP-S9E, Naga_100229g9	tr|K8Z8Z7|RP-S9E	1.98	0.248	0.39	FALSE	FALSE	TRUE	1	Small subunit ribosomal protein S9e
SBP	tr|W7TFY2|SBP	3.46	0.0447	0.199	FALSE	FALSE	TRUE	1	Chloroplast sedoheptulose--bisphosphatase
SEC11, Naga_100086	tr|K8ZB45|SEC11	1.81	0.0983	0.257	FALSE	FALSE	TRUE	4	Signal peptidase I

g7, Naga_100086 g8									
SHMT2	tr|W7TN09|SHMT2	4.22	0.0016	0.0571	FALSE	FALSE	TRUE	2	Serine hydroxymethyltransferase
SHMT2.1	tr|W7U077|SHMT2	0.76	0.485	0.596	FALSE	FALSE	FALSE	0	Serine hydroxymethyltransferase
TPI	gi|553184237|ref|XP_005854056.1|	1.68	0.124	0.282	FALSE	FALSE	FALSE	0	triosephosphate isomerase
tufA	tr|T1RJ79|tufA	0.862	0.425	0.543	FALSE	FALSE	FALSE	0	Elongation factor Tu, chloroplastic
UGD	AQR53217.1	2.5	0.0244	0.152	FALSE	FALSE	FALSE	0	UDP-glucose 6-dehydrogenase
XBP3	tr|W7TP29|XBP3	3.08	0.0194	0.146	FALSE	FALSE	TRUE	2	Fructose--bisphosphatase
